# A hypoallergenic peptide mix containing T cell epitopes of the clinically relevant house dust mite allergens

**DOI:** 10.1111/all.13956

**Published:** 2019-10-03

**Authors:** Huey‐Jy Huang, Mirela Curin, Srinita Banerjee, Kuan‐Wei Chen, Tetiana Garmatiuk, Yvonne Resch‐Marat, Claudia Carvalho‐Queiroz, Katharina Blatt, Guro Gafvelin, Hans Grönlund, Peter Valent, Raffaela Campana, Margarete Focke‐Tejkl, Rudolf Valenta, Susanne Vrtala

**Affiliations:** ^1^ Division of Immunopathology, Department of Pathophysiology and Allergy Research, Center for Pathophysiology, Infectiology and Immunology Medical University of Vienna Vienna Austria; ^2^ Department of Clinical Neuroscience, Therapeutic Immune Design Unit Karolinska Institutet Stockholm Sweden; ^3^ Division of Hematology&Hemostaseology, Department of Internal Medicine I Medical University of Vienna Vienna Austria; ^4^ NRC Institute of Immunology FMBA of Russia Moscow Russia; ^5^ Department of Clinical Immunology and Allergy, Laboratory for Immunopathology Sechenov First Moscow State Medical University Moscow Russia

**Keywords:** allergen, house dust mite allergy, recombinant allergen, synthetic peptide, T cell epitope

## Abstract

**Background:**

In the house dust mite (HDM) *Dermatophagoides pteronyssinus,* Der p 1, 2, 5, 7, 21, and 23 have been identified as the most important allergens. The aim of this study was to define hypoallergenic peptides derived from the sequences of the six allergens and to use the peptides and the complete allergens to study antibody, T cell, and cytokine responses in sensitized and nonsensitized subjects.

**Methods:**

IgE reactivity of HDM‐allergic and non‐HDM‐sensitized individuals to 15 HDM allergens was established using ImmunoCAP ISAC technology. Thirty‐three peptides covering the sequences of the six HDM allergens were synthesized. Allergens and peptides were tested for IgE and IgG reactivity by ELISA and ImmunoCAP, respectively. Allergenic activity was determined by basophil activation. CD4+ T cell and cytokine responses were determined in PBMC cultures by CFSE dilution and Luminex technology, respectively.

**Results:**

House dust mite allergics showed IgE reactivity only to complete allergens, whereas 31 of the 33 peptides lacked relevant IgE reactivity and allergenic activity. IgG antibodies of HDM‐allergic and nonsensitized subjects were directed against peptide epitopes and higher allergen‐specific IgG levels were found in HDM allergics. PBMC from HDM‐allergics produced higher levels of IL‐5 whereas non‐HDM‐sensitized individuals mounted higher levels of IFN‐gamma, IL‐17, pro‐inflammatory cytokines, and IL‐10.

**Conclusion:**

IgG antibodies in HDM‐allergic patients recognize peptide epitopes which are different from the epitopes recognized by IgE. This may explain why naturally occurring allergen‐specific IgG antibodies do not protect against IgE‐mediated allergic inflammation. A mix of hypoallergenic peptides containing T cell epitopes of the most important HDM allergens was identified.

## Graphical abstract

A mix of thirty‐one hypoallergenic peptides containing T cell epitopes of the six most important house dust mite (HDM) allergens was identified. Natural IgG antibodies of HDM‐allergic patients recognize different epitopes than IgE antibodies. Hypoallergenic T cell epitope‐containing peptides comprising the important HDM allergens may be used for therapy and prevention of HDM allergy.

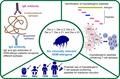

## 1 INTRODUCTION

House dust mites (HDMs) are one of the most important elicitors of immunoglobulin E (IgE)‐associated allergies worldwide.^1^ HDM‐allergic patients suffer from severe and chronic respiratory symptoms such as rhinitis and asthma as well as from skin symptoms, mainly atopic dermatitis.^2^, ^3^, ^4^ *Dermatophagoides pteronyssinus* (Der p) and *D. farinae* (Der f) are the most important mite species and contain more than 30 different allergen molecules.^5^, ^6^, ^7^ Der p and Der f allergens show extensive cross‐reactivity and several allergens seem to have intrinsic properties promoting their ability to induce allergic sensitization.^8^ Assessment of IgE to individual Der p allergens in cross‐sectional population studies as well as in longitudinal birth cohort studies have shown that Der p 1, Der p 2, Der p 4, Der p 5, Der p 7, Der p 21, and Der p 23 are the most frequently recognized allergens.^9^, ^10^, ^11^, ^12^, ^13^ Among those, Der p 1, 2, 5, 7, 21, and 23 seem to be clinically most relevant, as they were shown to comprise the majority of IgE epitopes of the HDM proteome.^14^

At present, several molecular approaches for allergen‐specific immunotherapy (AIT) and prevention are under development.^15^, ^16^, ^17^ They include recombinant hypoallergens targeting B cell as well as T cell responses, B‐cell epitope‐targeting vaccines based on fusion proteins, which consist of allergen‐derived peptides fused to a nonallergenic carrier protein^18^, ^19^, ^20^ and, nonallergenic peptides comprising the T cell epitope repertoire for the induction of therapeutic as well as preventive immune tolerance.^16^, ^21^ Approaches that target allergen‐specific T cells with synthetic peptides are mainly based on small peptides of less than 20 amino acids to avoid IgE sensitization, IgE recognition, and associated side effects.^21^, ^22^ With such an approach it is difficult, or even impossible, to cover the majority of T cell epitopes of several allergens with a reasonable number of synthetic peptides. Therefore, we have synthesized a panel of 33 peptides ranging from 27 to 43 amino acids, which allowed us to cover the sequences of the most important HDM allergens, that is Der p 1, 2, 5, 7, 21, and 23. These peptides and the complete allergen molecules were used to study allergen/peptide‐specific IgE and IgG responses, as well as T cell and cytokine responses to the allergens and peptides in HDM‐allergic patients and subjects without HDM sensitization. Our study identified a cocktail of 31 defined synthetic hypoallergenic peptides containing T cell epitopes of the six important HDM allergens. This peptide mix might be suitable for T cell‐directed approaches to induce immune tolerance for therapy and prevention of HDM allergy. It also revealed interesting differences regarding allergen‐specific antibody responses in allergic and nonallergic subjects that explain why natural allergen‐specific IgG antibodies do not protect against HDM allergy.

## 2 METHODS

### 2.1 Allergens and synthetic allergen‐derived peptides

**Figure 1:**
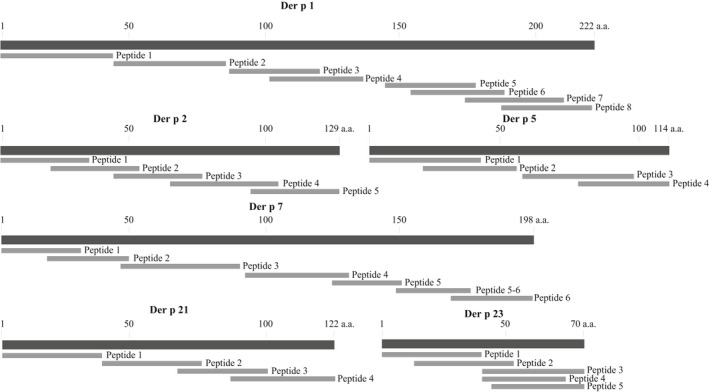
Overview of peptides spanning the sequences of Der p 1, 2, 5, 7, 21, and 23. Allergens are shown as black bars from the N (left) to the C terminus (right) in the same scale indicating the first, the last and every 50^th^ amino acid. Peptides are indicated as grey bars and are numbered for each allergen

Natural Der p 1 was obtained from Prof. WR Thomas (Telethon Kids Institute, The University of Western Australia, Perth, WA Australia). It was purified by affinity chromatography as described.^23^ Recombinant Der p 2, Der p 5, Der p 7, Der p 21, and Der p 23 were expressed and purified as described.^14^ Endotoxin was removed from purified allergens using a polymyxin resin (Affi‐Prep Polymyxin Matrix, Bio‐Rad) and/or by using an Endotoxin Removal Kit (ProteoSpin™, Endotoxin Removal Maxi Kit; Norgen Biotek). Remaining endotoxin was measured with the Limulus‐Amebocyte‐Lysate assay (Lonza). The endotoxin contents of Der p 2, 5, 7, 21, and 23 were <250 Endotoxin Units (EU)/mL and the endotoxin content of Der p 1 was 1300 EU/mL. Thirty‐three peptides, spanning the sequences of Der p 1, 2, 5, 7, 21, and 23 (Figure 1, Table S1), were synthesized on a peptide synthesizer (Liberty, CEM Corporation and Apex 396, aapptec) using Fmoc chemistry to protect amino acids (Merck Chemicals and Life Science GmbH) and HBTU (Carl Roth) for peptide coupling. The length of the peptides ranges from 27 to 43 amino acids and their characteristics are shown in Table S1. Peptides were purified by HPLC (Dionex UltiMate 3000; Thermo Fisher Scientific) and their molecular weights confirmed by MALDI‐TOF (Microflex, Bruker). Purified peptides (>85%) with correct molecular weight were dissolved in endotoxin‐free water and stored at −20°C.

### 2.2 HDM‐allergic patients and control subjects

Twenty‐seven HDM‐allergic patients, five non‐HDM‐sensitized allergic patients, and five nonallergic individuals were analyzed. The subjects were recruited by advertisement with approval of the Ethics Committee of the Medical University of Vienna (EK 1641/2014). Symptoms of allergy were recorded using ISAAC‐based questionnaires.^24^ Blood samples (serum, heparinized blood) were obtained from the subjects after written informed consent was obtained. The demographic and clinical characteristics of the subjects are summarized in Table S2. The 27 HDM‐allergic patients had a positive case history and perennial airway symptoms indicative for HDM allergy (ie, rhinitis, conjunctivitis, asthma, and/or atopic dermatitis; Table S2). Patients' treatments included measures for allergen avoidance, systemic or topical antihistamines and/or topical steroids but none of the patients had received AIT during the last 5 years or had systemic intake of steroids. The presence of specific IgE antibodies to *D. pteronyssinus* was determined by ImmunoCAP (d1 ImmunoCAP, Thermo Fisher Scientific/Phadia, Uppsala, Sweden) and was ≥0.7 kUA/L. All HDM‐allergic patients showed IgE reactivity to at least one of the 13 *D. pteronyssinus* allergens which were present on the MeDALL allergen chip^13^, ^25^ as determined by ImmunoCAP ISAC technology (Thermo Fisher Scientific/Phadia). The five non‐HDM‐sensitized subjects and five nonallergic subjects were negative to HDM allergen molecules in ImmunoCAP ISAC (Table S3). The five non‐HDM‐sensitized and five nonallergic subjects were combined to obtain a group of 10 non‐HDM‐sensitized subjects. Table S3 provides the complete IgE sensitization profiles to 163 microarrayed allergens of all patients and control subjects analyzed in this study.

### 2.3 ELISA and ImmunoCAP assays

Microplates (MaxiSorp; Thermo Fisher Scientific) were coated with 0.5 µg/well (in 100 µL PBS) of the individual HDM allergens or peptides overnight at 4°C. Microplates were washed three times with phosphate‐buffered saline with Tween‐20 (PBST) and nonspecific binding sites were blocked for 3 hours at RT with PBST containing 1% BSA (Carl Roth). Sera and antibodies were diluted with PBST containing 0.5% BSA. Microplates were incubated with HDM‐allergic patients' sera or non‐HDM‐sensitized subjects' sera, diluted 1:5 for IgE measurement and 1:10 for IgG measurement, overnight at 4°C. For the detection of IgE reactivity, plates were incubated with horseradish peroxidase (HRP)‐coupled goat anti‐human IgE (SeraCare/KPL) at a 1:2500 dilution overnight at 4°C. For the detection of bound IgG antibodies, microplates were incubated with mouse anti‐human IgG (Clone G18‐145 (RUO), BD biosciences) at a 1:1000 dilution overnight at 4°C followed by HRP‐coupled sheep anti‐mouse IgG antibodies (GE Healthcare Life Sciences) at a 1:2000 dilution for 1 hour at 37°C. The color reaction was performed by adding ABTS substrate solution, containing 30 mg ABTS (2,2′‐azino‐bis(3‐ethylbenzthiazoline‐6‐sulfonic acid) diammonium salt; Sigma‐Aldrich, St. Louis, Missouri), 3 µL H_2_O_2_, 0.38 g citric acid, 0.41 g sodium phosphate dibasic dihydrate in 30 mL ddH_2_O, and optical densities (OD) were measured at a wavelength of 405 nm in an ELISA reader (SpectraMax Plus; Molecular Devices). Results are expressed as means of duplicates with a deviation of less than 10%. Cutoff values of IgE reactivity were defined as mean OD plus three standard deviations of ten sera from non‐HDM‐sensitized individuals and were as follows: Der p 1: 0.14, Der p 2: 0.13, Der p 5: 0.22, Der p 7: 0.39, Der p 21: 0.17, Der p 23: 0.26. The 2‐fold means of the buffer control (ie, buffer without addition of serum) served as a cutoff for specific IgG.

Streptavidin immunoCAPs (o212 immunoCAP; Thermo Fisher Scientific/Phadia) were used to measure serum IgE levels specific for a mixture of Der p 1, 2, 5, 7, 21, and 23, and a mixture of 31 hypoallergenic peptides derived from these allergens as described.^14^

A customized version of the ImmunoCAP ISAC microarray chip (MeDALL‐chip) was produced by Phadia Austria GmbH.^25^, ^26^ Detection of IgE binding was performed as described^25^ and levels of allergen‐specific IgE antibodies are shown in ISAC Standardized Units (ISU) with a cutoff of 0.3 ISU.

### 2.4 Basophil activation tests

Heparinized blood samples from 7 HDM‐allergic patients and a nonallergic individual were collected by venipuncture. Ninety microliters of peripheral blood were incubated with 10 µL of different concentrations of the mixture of Der p 1, 2, 5, 7, 21, and 23 (mixture: 0.6‐600 ng/mL; each allergen 0.1‐100 ng/mL) or with an approximately equimolar mixture of the 31 hypoallergenic peptides (mixture: 0.76‐760 ng/mL) for 15 minutes at 37°C. The individual peptides were added to the mix according to the ratio molecular weight of the peptide/molecular weight of the corresponding allergen (Table S4). A monoclonal anti‐IgE antibody (1 µg/mL) was used as a positive control. Upregulation of CD203c expression on basophils was measured by flow cytometry as reported.^27^ The stimulation index (SI) was calculated as a ratio of mean fluorescence intensity of allergen or peptide mixture‐treated basophils to mean fluorescence intensity of unstimulated basophils.

### 2.5 PBMC stimulation assays

PBMCs were isolated by Ficoll density gradient centrifugation (Ficoll‐Paque PLUS, GE Healthcare) and labeled with 1 µmol/L carboxyfluorescein diacetate succinimidyl ester (CFSE; Thermo Fisher Scientific/Invitrogen).^28^ Aliquots of 200 000 CFSE‐labeled PBMCs were cultured in 200 µL of Ultra Culture serum‐free medium (Lonza) which was supplemented with 2 mmol/L l‐Glutamine, 20 µg/mL Gentamycin, 50 mol/L 2‐Mercaptoethanol, and 10% fetal bovine serum (Thermo Fisher Scientific/Invitrogen). CFSE‐labeled PBMCs were stimulated in triplicates with the individual allergens (12 mol/L; Der p 1, Der p 2, Der p 5, Der p 7, Der p 21, and Der p 23; ie, 0.3 µg/mL Der p 1, 0.17 µg/mL Der p 2, 0.17 µg/mL Der p 5, 0.26 µg/mL Der p 7, 0.17 µg/mL Der p 21, and 0.1 µg/mL Der p 23), with equimolar concentrations of the individual peptides (ie, Der p 1 peptide 1: 51.42 ng/mL; peptide 2: 57.21 ng/mL; peptide 3: 44.23 ng/mL; peptide 4: 52.32 ng/mL; peptide 5: 44.28 ng/mL; peptide 6: 45.4 ng/mL; peptide 7: 46.49 ng/mL; peptide 8: 49.57 ng/mL, Der p 2 peptide 1: 44.03 ng/mL; peptide 2: 41.78 ng/mL; peptide 3: 40.74 ng/mL; peptide 4: 58.16 ng/mL; peptide 5: 40.17 ng/mL, Der p 5 peptide 1: 45.36 ng/mL; peptide 2: 53.42 ng/mL; peptide 3: 45.28 ng/mL; peptide 4: 46.07 ng/mL, Der p 7 peptide 1: 41.30 ng/mL; peptide 2: 43.04 ng/mL; peptide 3: 42.42 ng/mL; peptide 4: 50.0 ng/mL; peptide 5: 35.84 ng/mL; peptide 5‐6: 36.31 ng/mL; peptide 6: 40.81 ng/mL, Der p 21 peptide 1: 51.41 ng/mL; peptide 2: 54.39 ng/mL; peptide 3: 44.9 ng/mL; peptide 4: 49.21 ng/mL, Der p 23 peptide 1: 44.78 ng/mL; peptide 2: 49.78 ng/mL; peptide 3: 57.56 ng/mL; peptide 4: 41.76 ng/mL and peptide 5: 43.46 ng/mL), T cell activator (Dynabeads Human T‐Activator CD3/CD28; Thermo Fisher Scientific/Invitrogen) as positive control or remained unstimulated (medium control). After 7 days of incubation at 37°C, cells were collected and nonspecific binding sites were blocked with 10% normal mouse serum (Thermo Fisher Scientific/Invitrogen) prior to staining with phycoerythrin/cyanine 7‐labeled mouse IgG1 anti‐human CD3, 7‐amino‐actinomycin D (7‐AAD) and phycoerythrin‐labeled anti‐human CD4. To evaluate fluorochrome unspecific staining, isotype controls conjugated to corresponding fluorochromes were used (all fluorophore conjugated antibodies, isotype controls, and viability dyes were purchased from BioLegend, San Diego, California). Live lymphocytes were gated as 7‐AAD^neg^, and CD4+ T lymphocytes were selected as a CD3^pos^CD4^pos^ population. The percentage of proliferating CD4+ T cells was calculated as the percentage of allergen‐ or peptide‐treated proliferating cells minus the percentage of unstimulated proliferating cells.

### 2.6 Cytokine measurements

The levels of G‐CSF, GM‐CSF, IFN‐gamma, IL‐1 beta, IL‐2, IL‐4, IL‐5, IL‐6, IL‐7, IL‐8, IL‐10, IL‐12 (p70), IL‐13, IL‐17, MCP‐1, MIP‐1beta, and TNF‐alpha were measured in supernatants of 7 day PBMC cultures by Luminex multiplex assay (Bio‐Plex Pro™ Human Cytokine 17‐plex Assay; Bio‐Rad) according to the manufacturer's instructions as described.^29^ The Bio‐Plex^®^ 200 System (Bio‐Rad) was used for analysis.

### 2.7 Statistical analysis

Mann‐Whitney U test was performed to analyze the differences between HDM‐allergic patients and non‐HDM‐sensitized individuals. A *P* value of less than 0.05 was considered as statistically significant.

## 3 RESULTS

### 3.1 Identification of non‐IgE‐reactive peptides comprising the sequences of the clinically important HDM allergens

**Figure 2:**
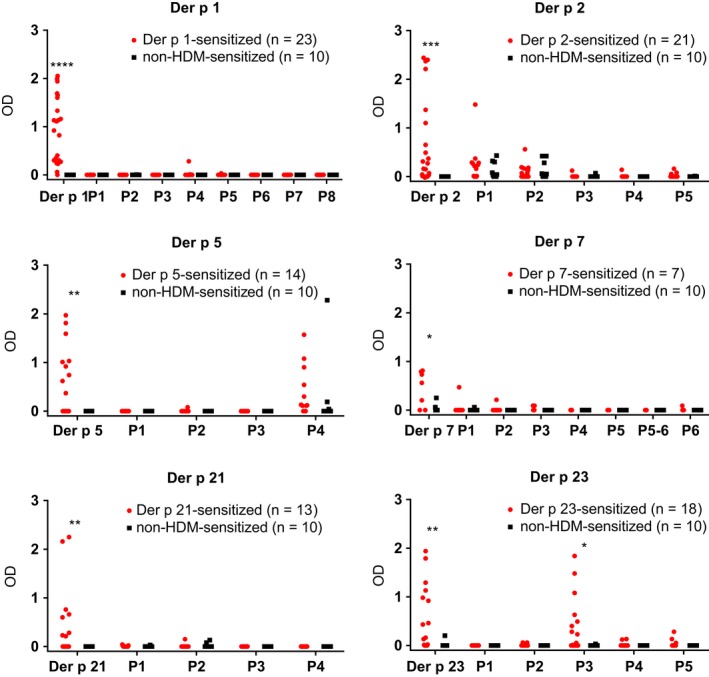
IgE reactivity of Der p allergens and allergen‐derived peptides. Allergens (Der p 1, Der p 2, Der p 5, Der p 7, Der p 21, and Der p 23) and allergen‐derived peptides (*x*‐axes) were tested by ELISA for IgE reactivity (*y*‐axes: OD values correspond to specific IgE levels) with sera from individuals sensitized to the respective allergen (red) and individuals without HDM sensitization (black). Statistically significant differences (****P < 0.0001; ***P < 0.001; **P < 0.01; *P < 0.05) are indicated

**Figure 3:**
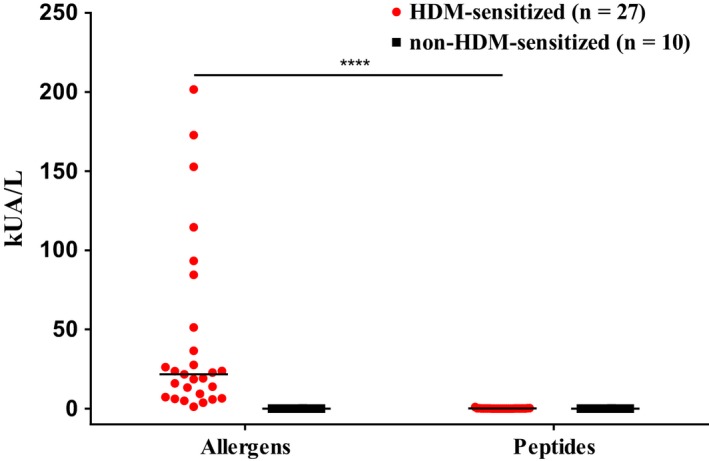
Quantification of allergen‐ and peptide‐specific IgE. IgE levels (y‐axis: kUA/L) specific for six Der p allergens (Der p 1, 2, 5, 7, 21, and 23) or allergen‐derived peptides (*x*‐axis) were measured by ImmunoCAP in sera from HDM‐allergic patients (red) and non‐HDM‐sensitized individuals (black). Statistically significant differences (****P < 0.0001) are indicated

Figure 2 shows the comparison of the six important HDM allergens with the allergen‐derived peptides regarding IgE reactivity with sera from HDM‐allergic patients (Table S2). According to ELISA and ImmunoCAP ISAC testing (Table S3), we found the following frequencies of allergen‐specific IgE recognition in the group of HDM‐sensitized patients: Der p 1:85.2%; Der p 2:77.8%; Der p 5:51.9%; Der p 7:25.9%; Der p 21:48.1%; Der p 23:66.7%. None of the non‐HDM‐sensitized allergic patients and none of the nonallergic individuals showed IgE reactivity to any of the six HDM allergens in the ELISA and micro‐array (Table S3). Thus patients mounted IgE reactivity to the complete allergen molecules but only two of the 33 allergen‐derived peptides (ie, peptide 4 from Der p 5, peptide 3 from Der p 23) showed IgE reactivity (Figure 2). Accordingly, 31 peptides comprising almost the complete sequences of Der p 1, 2, 5, 7, 21, and 23 lacked IgE reactivity (Table S1). The lack of IgE reactivity of the mix of these 31 allergen‐derived peptides was confirmed by quantitative IgE measurements using ImmunoCAP technology (Figure 3). For this purpose, a biotinylated Der p allergen mix comprising Der p 1, 2, 5, 7, 21, and 23 and a biotinylated mix of the 31 peptides were tested for IgE reactivity with sera from HDM‐sensitized and non‐HDM‐sensitized individuals (Figure 3). All 27 HDM‐allergic patients showed IgE reactivity (IgE levels >0.35 kUA/L) to the Der p allergen mix, whereas no IgE reactivity (IgE levels <0.35 kUA/L) was found to the peptide mix for 26 of the 27 patients (Figure 3). Only one patient (patient PA23) with very high IgE levels to the Der p allergen mix (201.6 kUA/L) showed IgE reactivity (0.92 kUA/L) to the peptide mix. None of the non‐HDM‐sensitized subjects showed any IgE reactivity to the allergen or peptide mix (Figure 3).

### 3.2 The peptide mix is hypoallergenic as determined by basophil activation testing

**Figure 4:**
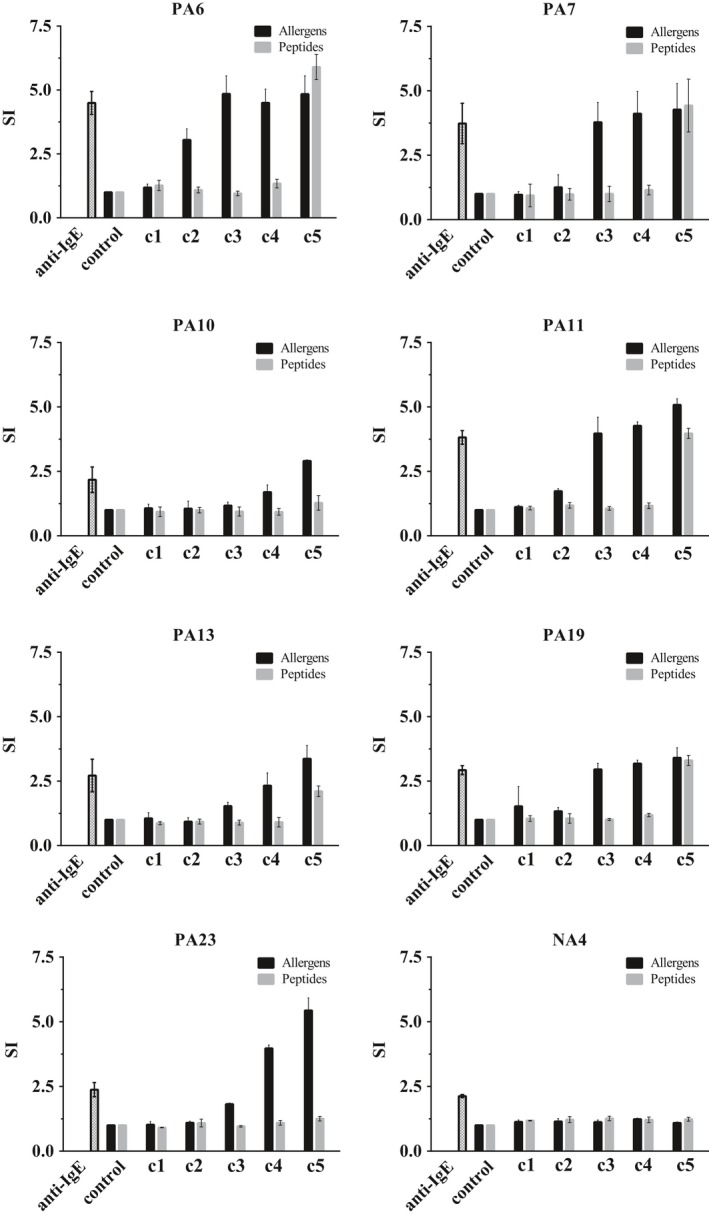
Comparison of the allergenic activity of Der p allergens and allergen‐derived peptides. Blood samples from seven HDM‐allergic patients (PA 6, 7, 10, 11, 13, 19, and 23) and one nonallergic individual (NA4) were incubated with anti‐IgE (1 µg/mL), medium buffer alone (control), different concentrations c1‐c5 (*x*‐axes: c1: 0.06 ng/mL, c2: 0.6 ng/mL, c3: 6 ng/mL, c4: 60 ng/mL, c5: 600 ng/mL) of mixtures containing the six Der p allergens (Der p 1, 2, 5, 7, 21, 23) (black) or approximately equimolar mixtures of the allergen‐derived peptides (c1: 0.076 ng/mL, c2: 0.76 ng/mL, c3: 7.6 ng/mL, c4: 76 ng/mL, c5: 760 ng/mL) (gray). Up‐regulations of CD203c expression on basophils are displayed as mean stimulation indices (SIs) ± SD (*y*‐axes)

The allergenic activity of the HDM allergens and the peptide mix was studied in basophil activation experiments by measuring the upregulation of CD203c expression. Basophils from seven HDM‐allergic patients and one nonallergic control (NA4) were incubated with five concentrations (0.06‐600 ng/mL) of the allergen mix containing Der p 1, 2, 5, 7, 21, and 23 or with an approximately equimolar mix (0.076‐760 ng/mL) of the 31 peptides (Figure 4). The concentrations of the peptides used in the basophil activation assays were thus indicative of the allergen mixture (see methods basophil activation, Table S4). The allergen mix induced dose‐dependent upregulation of CD203c expression in each of the tested HDM‐allergic patients (Figure 4). In contrast, no upregulation was observed with the peptide mix up to a concentration of 76 ng/mL (Figure 4). An upregulation of CD203c expression was only observed with the highest concentration (ie, 760 ng/mL) of the peptide mix in five of the seven patients (ie, patients 6, 7, 11, 13, and 19). This concentration was 100‐1000‐fold higher than the concentration of the allergen mix needed to induce CD203c upregulation. No upregulation of CD203c expression was obtained with the allergen mix or peptide mix in the nonallergic individual (NA4) up to a concentration of 600 ng/mL of allergens and 760 ng/mL of the peptide mix (Figure 4).

### 3.3 Allergen‐specific IgG levels are highest in patients who are sensitized to the same allergen and directed to sequential peptide epitopes

**Figure 5:**
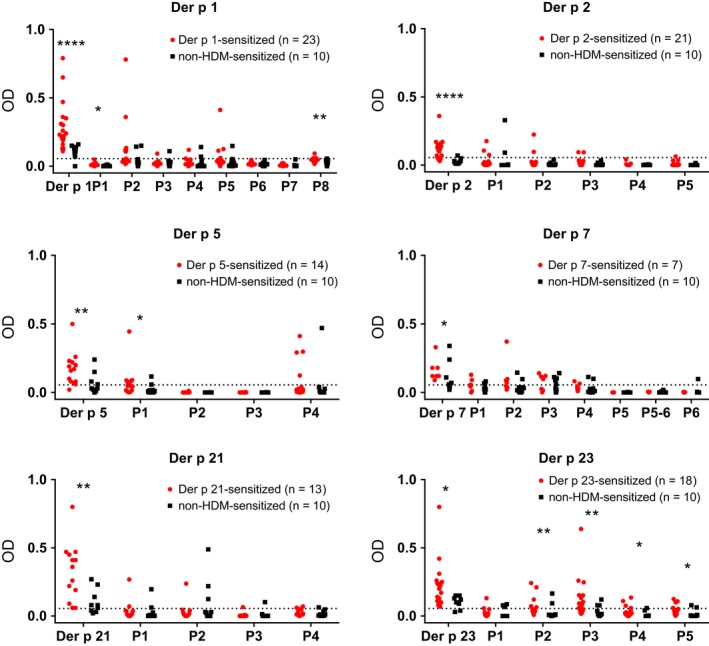
IgG reactivity of Der p allergens and allergen‐derived peptides. Allergens (Der p 1, Der p 2, Der p 5, Der p 7, Der p 21, and Der p 23) and allergen‐derived peptides (*x*‐axes) were tested for IgG reactivity (*y*‐axes: OD values correspond to specific IgG levels) with sera from individuals sensitized to the respective allergen (red) and individuals without HDM sensitization (black). The buffer control was subtracted from the data and cutoffs were represented by dashed lines. Statistically significant differences (****P < 0.0001; ***P < 0.001; **P < 0.01; *P < 0.05) are indicated

Figure 5 shows the IgG reactivity among patients who are also sensitized to the same allergen and of non‐HDM‐sensitized individuals to each of the six HDM allergens and the allergen‐derived peptides. Both, HDM‐allergic patients as well as non‐HDM‐sensitized subjects, showed IgG reactivity to the six HDM allergens (ie, Der p 1, 2, 5, 7, 21, and 23). However, allergen‐specific IgG levels were always significantly higher in the patients who were also sensitized to the same allergen (Der p 1: n = 23; Der p 2: n = 21; Der p 5: n = 14; Der p 7: n = 7; Der p 21: n = 13; Der p 23: n = 18) compared to non‐HDM‐sensitized subjects (Figure 5).

In contrast to IgE antibodies (Figures 2 and 3), IgG antibodies of allergic patients were directed to sequential peptide epitopes (Figure 5, Table S5). Also, non‐HDM‐sensitized subjects showed IgG reactivity to the allergen‐derived peptides (Figure 5, Table S5).

### 3.4 T cell reactivity to complete Der p allergens and allergen‐derived peptides in sensitized and nonsensitized individuals

**Figure 6:**
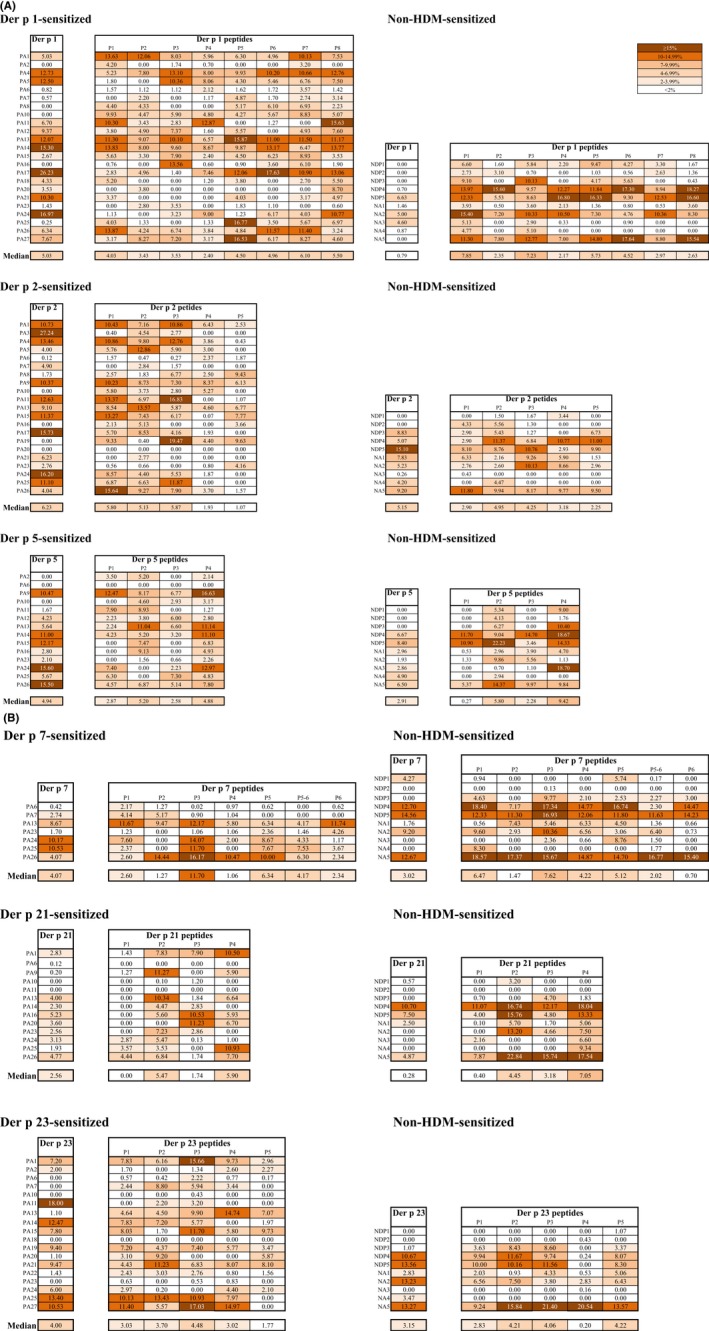
T cell reactivity of Der p allergens and allergen‐derived peptides for each subject. Shown are percentages of proliferated CD4+ T cell in responses to the allergens and allergen‐derived peptides (A, Der p 1, Der p 2, Der p 5, B, Der p 7, Der p 21, Der p 23; Means of triplicates; color codes for different percentages of proliferated cells are shown with a cut off of 2%‐white) in PBMC cultures from each allergen‐sensitized patient (PA), non‐HDM‐sensitized allergic individuals (NDP) and nonallergic individuals (NA). Median percentages are shown for each allergen and peptide for sensitized and nonsensitized individuals at the bottom of each Table

Figure S1 shows the percentages of proliferated CD4+ T cells in PBMCs cultured from HDM‐allergic and non‐HDM‐sensitized subjects when stimulated with six HDM allergens or the allergen‐derived peptides. The concentrations of the peptides used were indicative of the allergens (see Methods). HDM‐allergic patients as well as non‐HDM‐sensitized subjects showed quite comparable CD4+ T cell proliferation to each of the six Der p allergens (Figure S1). Responses to allergens seemed to be higher in allergic subjects, but only T cell proliferation to Der p 1 was significantly higher in Der p 1‐sensitized patients compared to non‐HDM‐sensitized subjects. Allergen‐derived peptides induced variable CD4+ T cell proliferation. For example, Der p 1‐derived peptide 1 or Der p 5‐derived peptide 4 induced stronger T cell responses in non‐HDM‐sensitized subjects than in patients who were sensitized to the corresponding allergen (Figure S1). Interestingly, certain peptides (eg, P3 from Der p 7, P2 from Der p 21) induced stronger T cell activation than the complete allergen molecule. Moreover, a detailed analysis of T cell responses to the allergens and peptides (Figure 6) showed that some HDM‐allergic and non‐HDM‐sensitized subjects showed little or no CD4+ T cell reactivity with the complete allergens but proliferated in response to allergen‐derived peptides (eg, Figure 6A, Der p 1: patients PA2, PA6‐8, PA10, PA16, PA23, PA25; non‐HDM‐sensitized subjects NDP1‐4; nonallergic subjects: NA1, NA4‐5; Der p 2: patients PA6, PA8, PA10, PA16, PA19; non‐HDM‐sensitized subjects NDP1‐2; Der p 5: patients PA2, PA10‐11; non‐HDM‐sensitized subjects NDP1‐3; nonallergic subject: NA2; Figure 6B, Der p 7: patients PA6, PA23; non‐HDM‐sensitized subject NDP3; nonallergic subjects: NA1; NA3‐4; Der p 21: patients PA9, PA25; non‐HDM‐sensitized subjects NDP1, NDP3; nonallergic subjects: NA2‐4; Der p 23: patients PA6‐7, PA13, PA20, PA22; non‐HDM‐sensitized subject: NDP3; Figure 6). A few patients showed no detectable T cell response to the peptides (eg, Der p 2: patient PA20; Der p 5: patient PA6; Der p 21: patients PA6, PA10, and PA11; Der p 23: patients PA10, PA18, and PA23) but these patients also showed no detectable T cell response to the complete allergens (Figure 6).

### 3.5 PBMC from HDM‐allergic patients produce higher levels of Th2 cytokines whereas non‐HDM‐sensitized individuals produce higher levels of IFN‐gamma, pro‐inflammatory cytokines and IL‐10 in response to allergens

**Figure 7:**
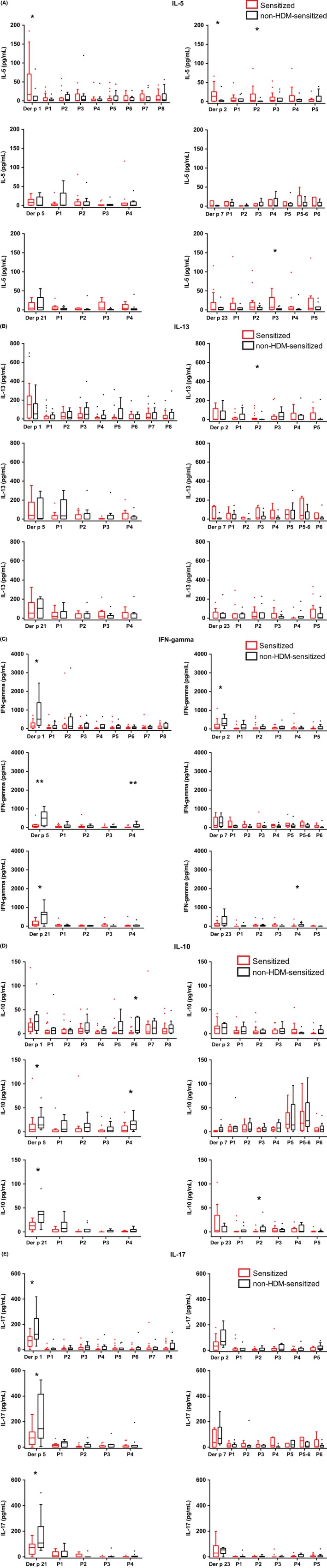
Cytokine and chemokine responses to Der p allergens and allergen‐derived peptides. Cultured PBMCs from individuals sensitized to the respective allergen (red) and individuals without HDM sensitization (black: n = 10) were incubated with allergens (Der p 1, Der p 2, Der p 5, Der p 7, Der p 21, Der p 23) and the corresponding allergen‐derived peptides (*x*‐axes) and cytokine and chemokine levels were measured in the supernatants (*y*‐axis: pg/mL). A, IL‐5, B, IL‐13, C, IFN‐gamma, D, IL‐10, E, IL‐17. Shown are box plots representing the first and third quartile. Horizontal bars denote medians and outliers are presented as circles. Statistically significant differences (**P < 0.01; *P < 0.05) are indicated

In parallel to the assessment of T cell proliferation, multiple cytokine responses to the allergens and peptides were determined in cultured PBMCs from HDM‐allergic patients and non‐HDM‐sensitized individuals. The results are presented in Figure 7A‐E and Figure S2A‐G for those cytokines where the levels were within the detection limits. Higher levels of the Th2 cytokines IL‐5 and IL‐13 were found in supernatants from PBMCs of HDM‐allergic subjects when stimulated with most of the complete allergens (ie, Der p 1, Der p 2, Der p 5, Der p 7, Der p 23) compared to supernatants from PBMCs of nonsensitized individuals (Figure 7A, 7B), reaching significance for IL‐5 in Der p 1 and Der p 2 (Figure 7A). Regarding Th2 cytokine responses to the peptides, we did not observe a uniform picture. Certain peptides induced higher levels of Th2 cytokines in allergic patients (eg, IL‐5: peptide 2 from Der p 2, peptides 3 and 4 from Der p 21, peptides 3 and 5 from Der p 23; IL‐13: peptides 5 and 5‐6 from Der p 7) whereas others induced higher levels of Th2 cytokines in nonsensitized subjects (eg, IL‐5: peptide 1 from Der p 5; IL‐13: peptide 3 from Der p 1, peptide 1 from Der p 5; Figure 7A, 7B).

The levels of the Th1 cytokine IFN‐gamma were higher in supernatants from allergen‐stimulated PBMCs of non‐HDM‐sensitized subjects than in those of allergic patients (Figure 7C). These differences were significant for Der p 1, Der p 2, Der p 5, and Der p 21. Regarding the counter‐regulatory/tolerogenic cytokine IL‐10 (Figure 7D), all allergens and most of the allergen‐derived peptides induced higher IL‐10 levels in cultured PBMCs from non‐HDM‐sensitized subjects compared to allergic subjects, significant for peptide 6 from Der p 1, peptide 4 from Der p 5 and peptide 2 from Der p 23 (Figure 7D). Allergen‐induced levels of IL‐17 and of the pro‐inflammatory cytokines IL‐1 beta, IL‐6 and TNF‐alpha were higher in supernatants of cultured PBMCs from non‐HDM‐sensitized subjects as compared to HDM‐allergic subjects (Figure 7E and Figure S2A‐C), significant for Der p 1 (IL‐17, IL‐6 and TNF‐alpha), Der p 5 (IL‐17, IL‐6, and TNF‐alpha) and Der p 21 (IL‐17, IL‐1 beta, and IL‐6). Similarly, as for the Th2 cytokines the picture was variable for peptide‐stimulated PBMCs regarding IFN‐gamma, IL‐10 and the pro‐inflammatory cytokines.

No apparent differences were found for chemokines GM‐CSF (Figure S2D), MCP‐1 (Figure S2E) and MIP‐1beta (Figure S2F) between allergic and nonsensitized subjects for allergens and peptides, except for G‐CSF (Figure S2G) which was elevated in non‐HDM‐sensitized subjects in response to full allergens (significant for Der p 21) and allergen‐derived peptides (significant for peptide 3, peptide 5, peptide 6 peptide 8 from Der p 1, peptide 1 from Der p 2 and peptide 3 from Der p 21).

The levels of the other cytokines determined by the Luminex technology were either very low (eg, IL‐2, IL‐4, IL‐7 and IL‐12 (p70)) or above the detection limit (IL‐8; data not shown).

## 4 DISCUSSION

Der p 1, 2, 5, 7, 21, and 23 have been identified as the most frequently recognized HDM allergens comprising the majority of HDM‐specific IgE epitopes.^14^ Therefore, they should be considered as components included in a therapeutic or prophylactic vaccine for HDM allergy. A major goal of our study was to identify a panel of synthetic hypoallergenic allergen‐derived peptides containing T cell epitopes of these allergens for therapeutic and preventive strategies which target T cells. Studies on T cell epitopes have so far only been conducted for Der p 1, 2, and 23 but not for the other clinically relevant HDM allergens, Der p 5, 7, and 21.^30^, ^31^, ^32^ This is the first study to compare the T cell responses to the panel of clinically relevant HDM allergens (Der p 1, 2, 5, 7, 21, and 23). In order to reduce the number of peptides needed for a tolerogenic peptide cocktail, we synthesized peptides which were longer (ie, 27‐43 aa) than the peptides used in previous T cell epitope‐based approaches (<20 aa).^21^, ^22^ Peptides were designed to span hydrophilic regions and the exact location and length of the peptides were adjusted to avoid difficulties during peptide synthesis. Some of the peptides have overlaps and it may therefore be possible to reduce the number of peptides needed for tolerance induction.

We then compared these peptides with the complete allergen molecules regarding specific IgE and IgG responses as well as T cell and cytokine responses to the allergens and peptides in HDM‐allergic patients and subjects without HDM sensitization. The testing for IgE reactivity and allergenic activity in basophil activation tests allowed us to identify a cocktail of 31 hypoallergenic peptides which contained T cell epitopes of the six allergens. These peptides had no or a strongly reduced allergenic activity (100‐1000‐fold) as determined by basophil activation assays and should be safe when administered but clinical dose‐finding studies will be needed to confirm the lack of in vivo allergenicity. Since some basophil activation was observed in certain donors at the highest concentration of the peptide mix, it cannot be excluded that the administration of the peptides may induce allergic reactions in highly sensitive patients. However, such patients may be identified by skin testing with the peptide mix.

When we analyzed IgG antibody responses toward the peptides and the complete allergens, we noticed that both HDM‐allergic as well as non‐HDM‐sensitized subjects showed IgG reactivity to the sequential peptide epitopes, whereas IgE antibodies of allergic patients recognized only the intact allergen molecules. The finding that IgE and IgG antibodies of HDM‐allergic patients recognize different epitopes is of clinical relevance because it may explain why naturally occurring allergen‐specific IgG antibodies do not protect against IgE‐mediated allergic inflammation. This is also of interest because it indicates that B cell populations with different antigen specificity are engaged in the production of allergen‐specific IgE and IgG antibodies. Whether class switch recombination to IgE production occurs in a sequential mode by a switching from IgM via IgG to IgE or by direct and independent switching from IgM either to IgG or IgE production is a matter of considerable debate,^33^ but our finding that IgE and IgG antibodies recognize different epitopes speaks for the latter scenario. HDM‐allergic patients with IgE sensitization to a certain allergen always had higher levels of IgG to this allergen than non‐HDM‐sensitized subjects. This finding is in agreement with an earlier study which has performed a similar comparison in HDM‐allergic children.^11^ This study also found that allergen‐specific IgG levels were higher in children who were sensitized to the same allergen than in children who were not sensitized, especially for Der p 1, Der p 2, and Der p 23. A similar result was obtained also in another study which showed that nonhospitalized, nonallergic individuals had low allergen‐specific IgG levels when compared to allergic subjects.^34^ We suggest that this could be due to a genetic restriction of allergen presentation to T cells which provide help to IgE as well as IgG production. HLA‐restricted allergen presentation has been suggested already earlier to be associated with IgE recognition of allergens.^35^, ^36^ A recent study performed in mice transgenic for an allergen‐specific human T cell receptor, for the corresponding human HLA molecule and for both, has also provided support for the existence of a genetic restriction of allergen recognition.^37^

In our study, we have defined a hypoallergenic peptide mix containing T cell epitopes of all clinically relevant HDM allergens including also Der p 5, 7, and 21. T cell epitopes were spread over the whole sequences of Der p 1, 2, 5, 7, 21, and 23, which was in agreement with former studies on T cell epitopes of Der p 1, 2, and 23.^30^, ^31^, ^38^, ^39^ However, the analysis of T cell responses to the allergens and peptides yielded also some interesting results. T cell responses to intact, IgE‐reactive allergens seemed to be higher in patients who were sensitized to the allergens, albeit not reaching statistical significance. Nevertheless, it may indicate that allergic subjects can present the intact, IgE‐reactive allergens more efficiently than nonsensitized subjects by IgE‐facilitated allergen presentation, a process which may be important for maintaining and triggering of allergen‐specific T cell responses in allergic patients.^40^, ^41^ Another interesting observation was that there were several examples that CD4+ T cells proliferated only to allergen‐derived peptides but not to the complete allergen at the concentration tested. This may be due to lack of proliferation to the one allergen concentration which had been tested. However, it may be also explained by the existence of allergen‐specific unconventional “type B” T cells^42^ which respond differently to peptides and complete antigens.

While there were no apparent differences regarding the peptide epitope specificities between allergic patients and nonallergic subjects, we observed that cytokine responses to the allergens in allergic patients were dominated by a Th2 profile characterized by higher levels of IL‐5. By contrast, nonsensitized subjects predominantely produced more IFN‐gamma, pro‐inflammatory cytokines, IL‐17 and the tolerogenic cytokine IL‐10.^43^ A limitation of our study was that we could measure the cytokine levels only at one time point, which may have been not optimal for all of the cytokines due to different kinetics of production and there may be also consumption of cytokines in the cultures. The latter may also explain differences to previous studies which have reported that levels of IFN‐gamma were higher or equal in PBMC from allergic compared to nonallergic subjects when stimulated with purified allergen molecules.^44^, ^45^, ^46^

There are at least two possibilities for how the hypoallergenic cocktail of synthetic peptides containing T cell epitopes of the six important HDM allergens may be used for allergy treatment. First, the peptides may be applied in a therapeutic setting as has been proposed for T cell peptide approaches earlier, for example for the treatment of cat allergy.^21^ However, different results were obtained for the therapeutic Fel d 1 peptide vaccination studies. Initial clinical trials were promising^47^ but the large phase III field study (https://www.circassia.com/media/press-releases/circassia-announces-top-line-results-from-cat-allergy-phase-iii-study/) did not reveal significant differences regarding clinical improvement between active and placebo‐treated patients.^48^ Therefore, we suggest to use the hypoallergenic peptide mix rather in a preventive setting by applying the peptides systemically either via the oral route^16^ or by injection,^49^ with the goal to prevent allergic sensitization early in childhood.

The production of a cocktail of more than 30 peptides may be difficult, but it might be possible to narrow the peptide set for clinical applications. Since some peptides (eg, peptide 4 and peptide 5 from Der p 23) showed weak IgE reactivity and some T cell epitopes may be lacking because not all peptides were overlapping, the exact location and length of some peptides need to be adapted. Modifications of certain peptide sequences will be necessary to improve solubility and stability as well as to prevent aggregation.^50^ For oral treatment, the peptides may need to be protected from degradation in the gastrointestinal tract.^51^ Additionally, one may consider to reduce the number of peptides by identifying dominant T cell epitopes.^50^ Nevertheless, our study may be considered as a first important step toward such an ambitious goal as it provides a set of hypoallergenic peptides containing T cell epitopes of the HDM allergens recognized by humans.

## CONFLICTS OF INTEREST

Dr. Valenta reports grants from Biomay AG, Vienna, Austria, grants from Austrian Science Fund, FWF, during the conduct of the study; grants and personal fees from Biomay AG, Vienna, Austria, grants from Austrian Science Fund, FWF, grants and personal fees from Viravaxx, Vienna, Austria, outside the submitted work. Dr. Vrtala reports grants from Austrian Science Fund, during the conduct of the study. All other authors declare that they have no conflicts of interest.

## AUTHOR CONTRIBUTIONS

HJH contributed to design of the study, performed experiments, interpreted the findings, and wrote the manuscript. SV and RV designed the study and contributed to the interpretation of the findings, writing and revising the manuscript. MC, SB, KWC, TG, and YRM kindly provided the allergens and peptides of HDMs, read and revised the manuscript. CCQ, GG, and HG designed the Luminex assay, participated in the interpretation of the findings, read and revised the manuscript. KB and PV designed and performed basophil activation testing, participated in the interpretation of the findings and revised the manuscript. RC and MFT contributed in interpretation of findings, read and revised the manuscript.
