# Supplementary figures and images for: A hypoallergenic peptide mix containing T cell epitopes of the clinically relevant house dust mite allergens
Source: Allergy. 2019 Oct 3;74(12):2461–78. doi: 10.1111/all.13956 (PMC7078969; doi:10.1111/all.13956)

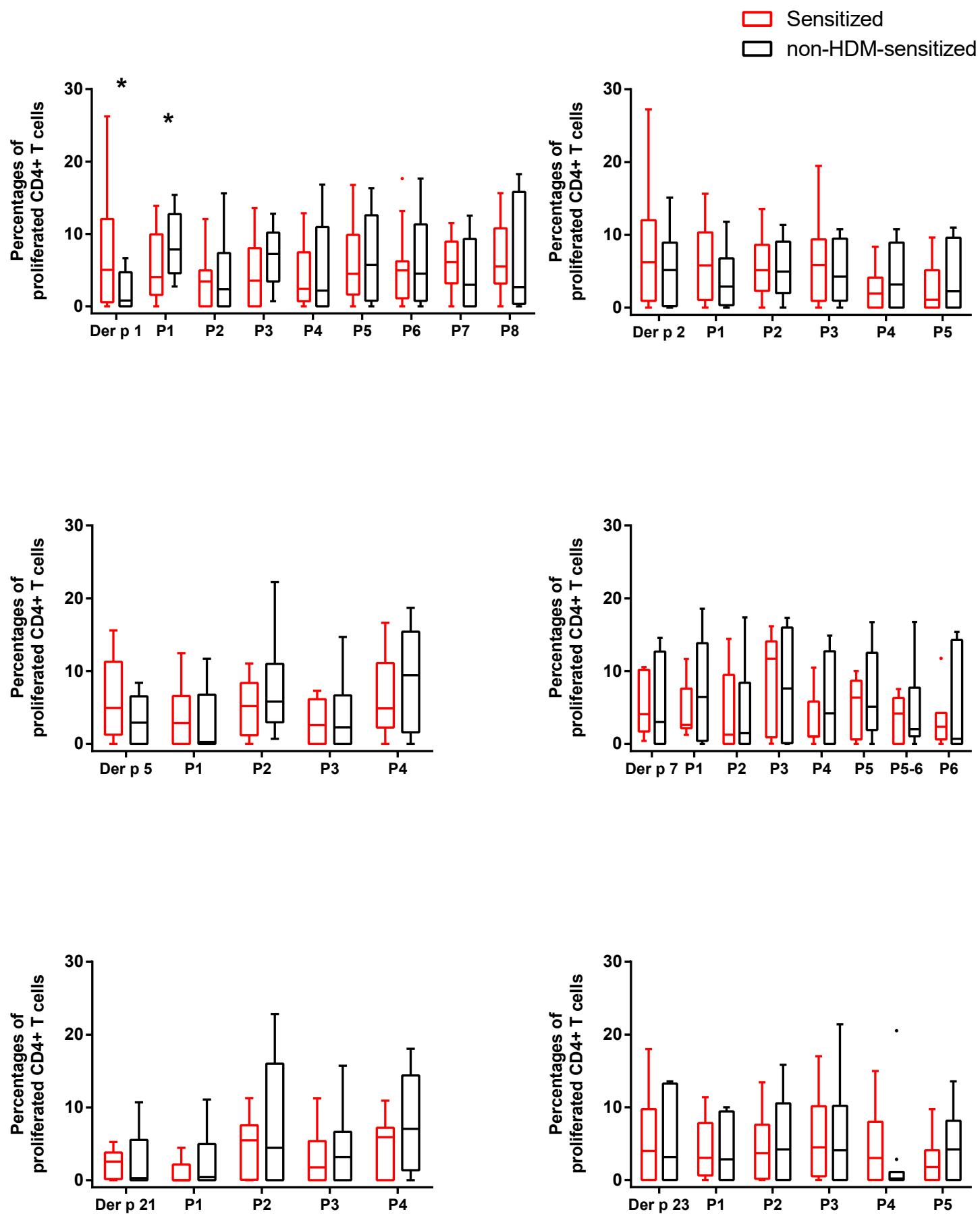

**Figure S1.**

Supplement: Supplementary file 1 [file ALL-74-2461-s001.pdf]
