# Supplementary material for: A hypoallergenic peptide mix containing T cell epitopes of the clinically relevant house dust mite allergens
Source: Allergy. 2019 Oct 3;74(12):2461–78. doi: 10.1111/all.13956 (PMC7078969; doi:10.1111/all.13956)
Supplement: Supplementary file 2 [file ALL-74-2461-s002.pdf]

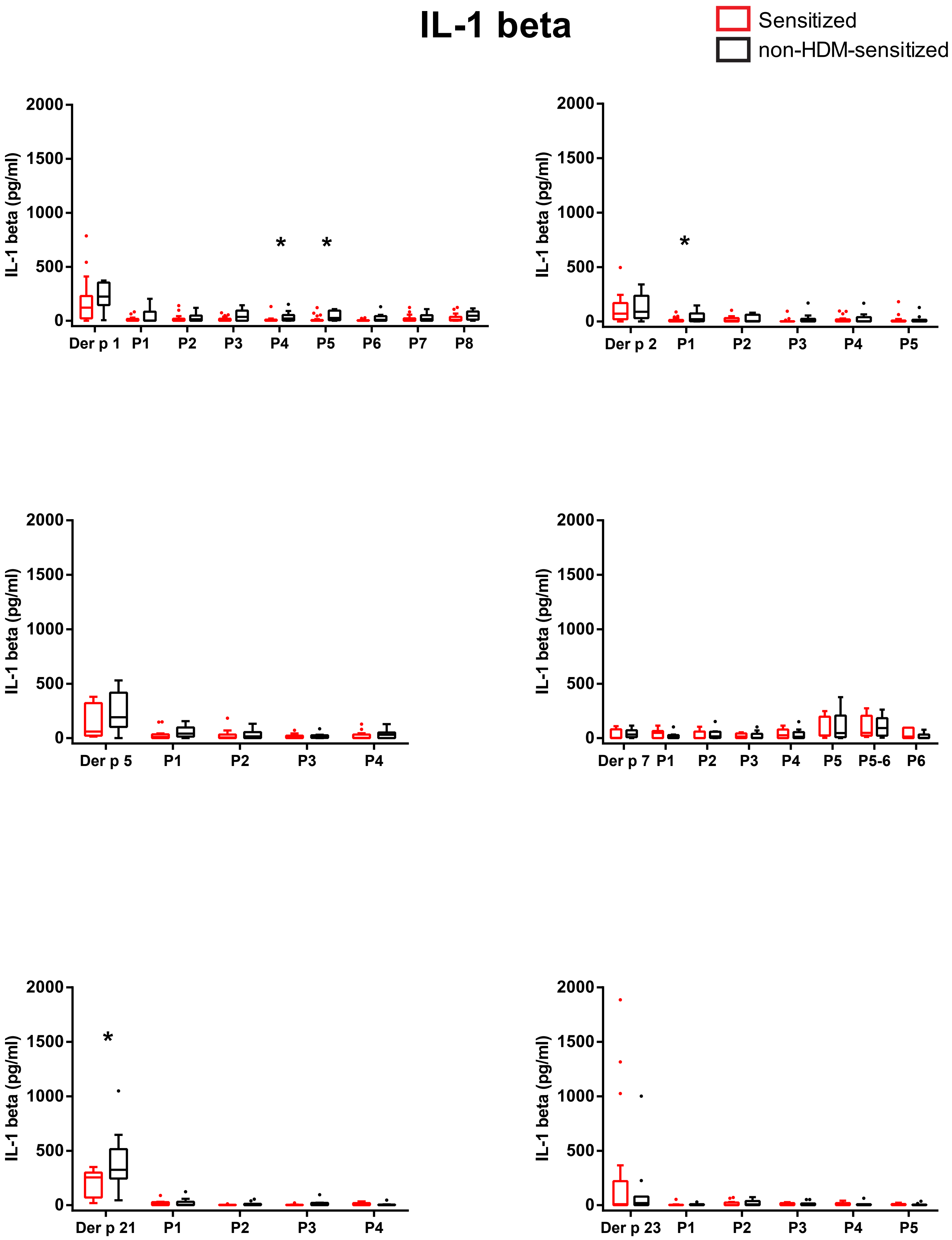

IL-1 beta
Sensitized
non-HDM-sensitized
IL-1 beta (pg/ml)
2000
1500
1000
500
0
Der p 1
P1
P2
P3
P4
P5
P6
P7
P8
*
*
Der p 2
P1
P2
P3
P4
P5
*
Der p 5
P1
P2
P3
P4
Der p 7
P1
P2
P3
P4
P5
P5-6
P6
Der p 21
P1
P2
P3
P4
*
Der p 23
P1
P2
P3
P4
P5

Figure S2A.

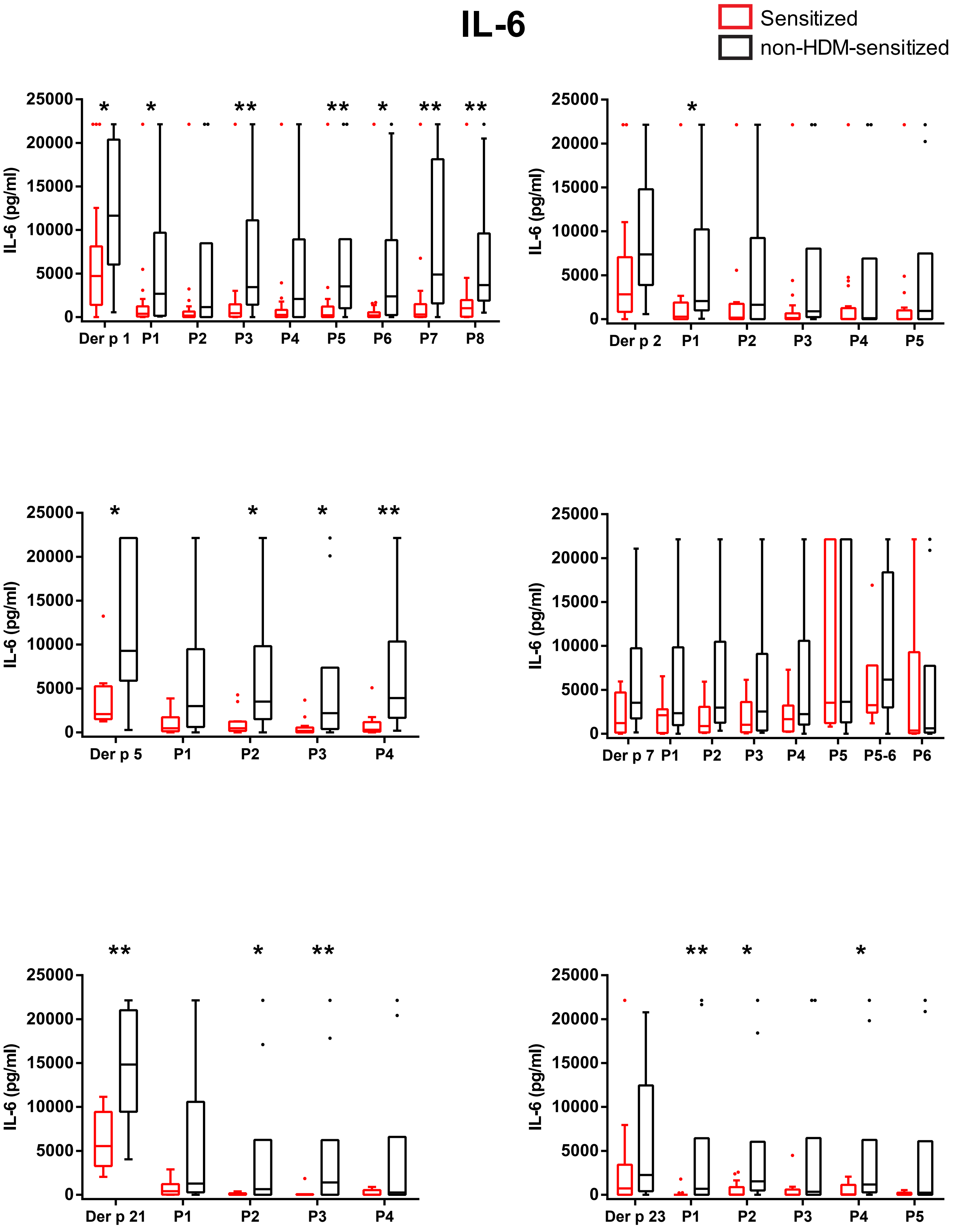
IL-6
Sensitized
non-HDM-sensitized
IL-6 (pg/ml)
25000
20000
15000
10000
5000
0
Der p 1
P1
P2
P3
P4
P5
P6
P7
P8
Der p 2
Der p 5
Der p 7
P5-6
Der p 21
Der p 23
*
**

Figure S2B.

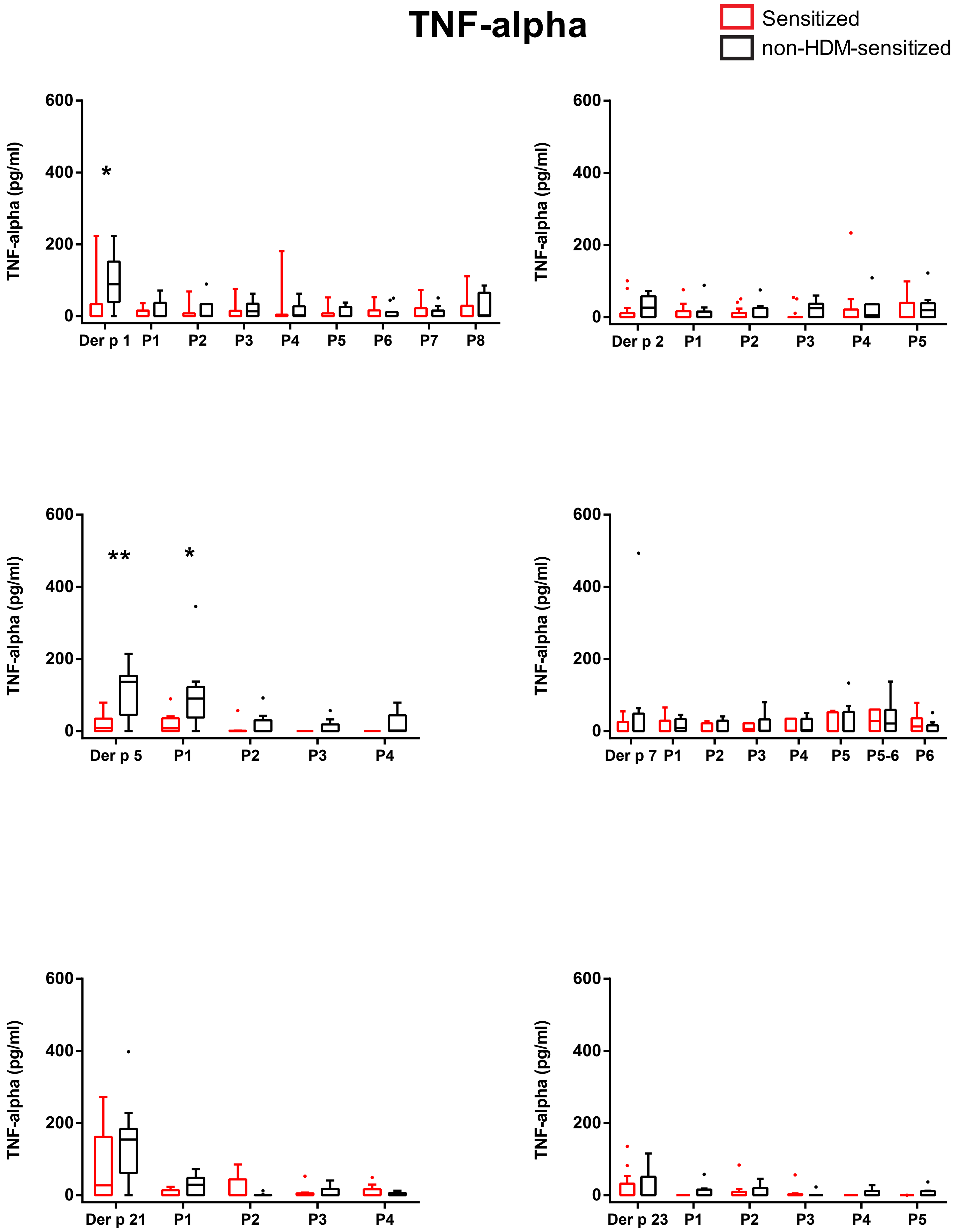
TNF-alpha
Sensitized
non-HDM-sensitized
TNF-alpha (pg/ml)
600
400
200
0
Der p 1
P1
P2
P3
P4
P5
P6
P7
P8
*
Der p 2
Der p 5
**
Der p 7
P5-6
Der p 21
Der p 23

Figure S2C.

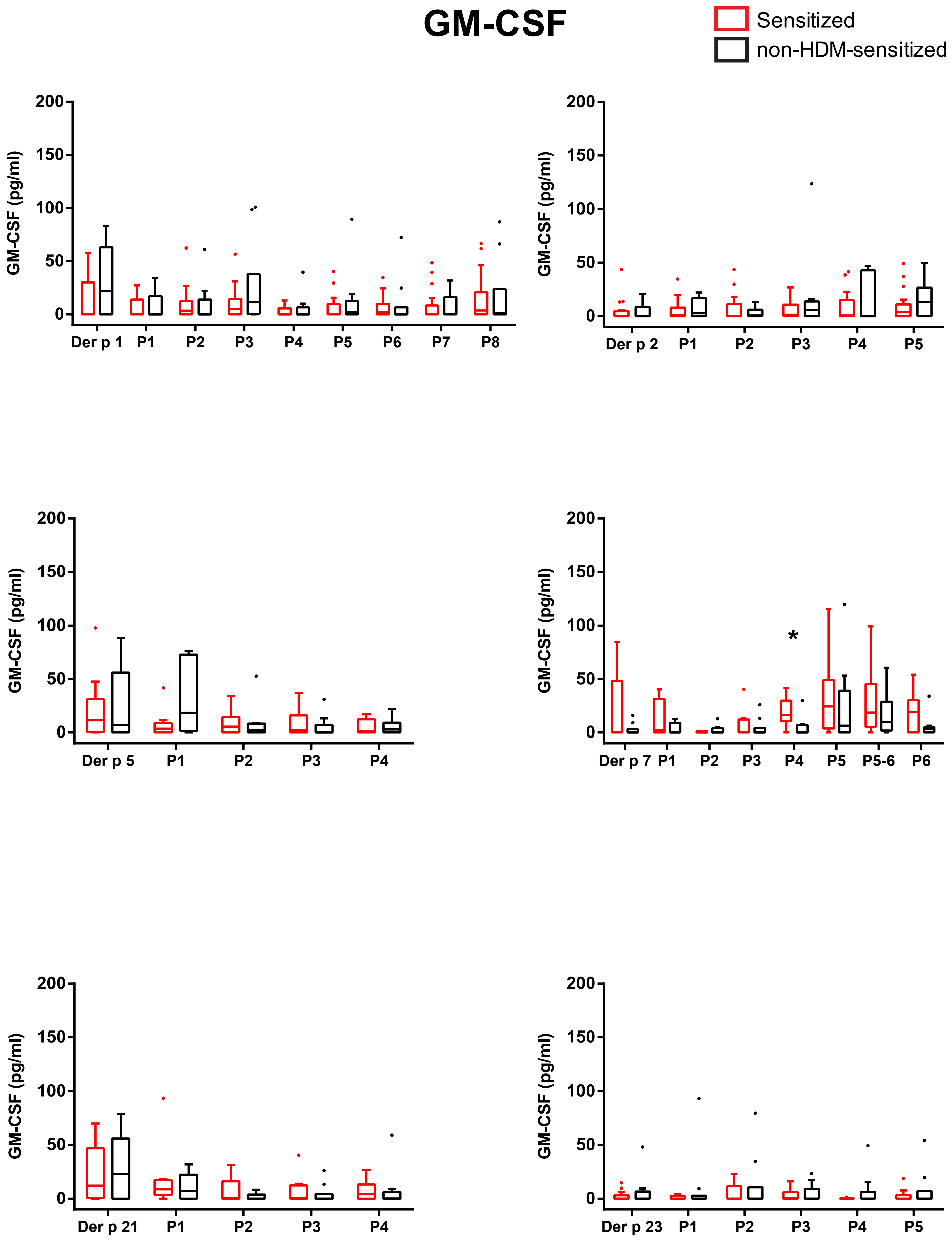
GM-CSF
Sensitized
non-HDM-sensitized
GM-CSF (pg/ml)
200
150
100
50
0
Der p 1
P1
P2
P3
P4
P5
P6
P7
P8
Der p 2
Der p 5
Der p 7
P5-6
Der p 21
Der p 23
*

Figure S2D.

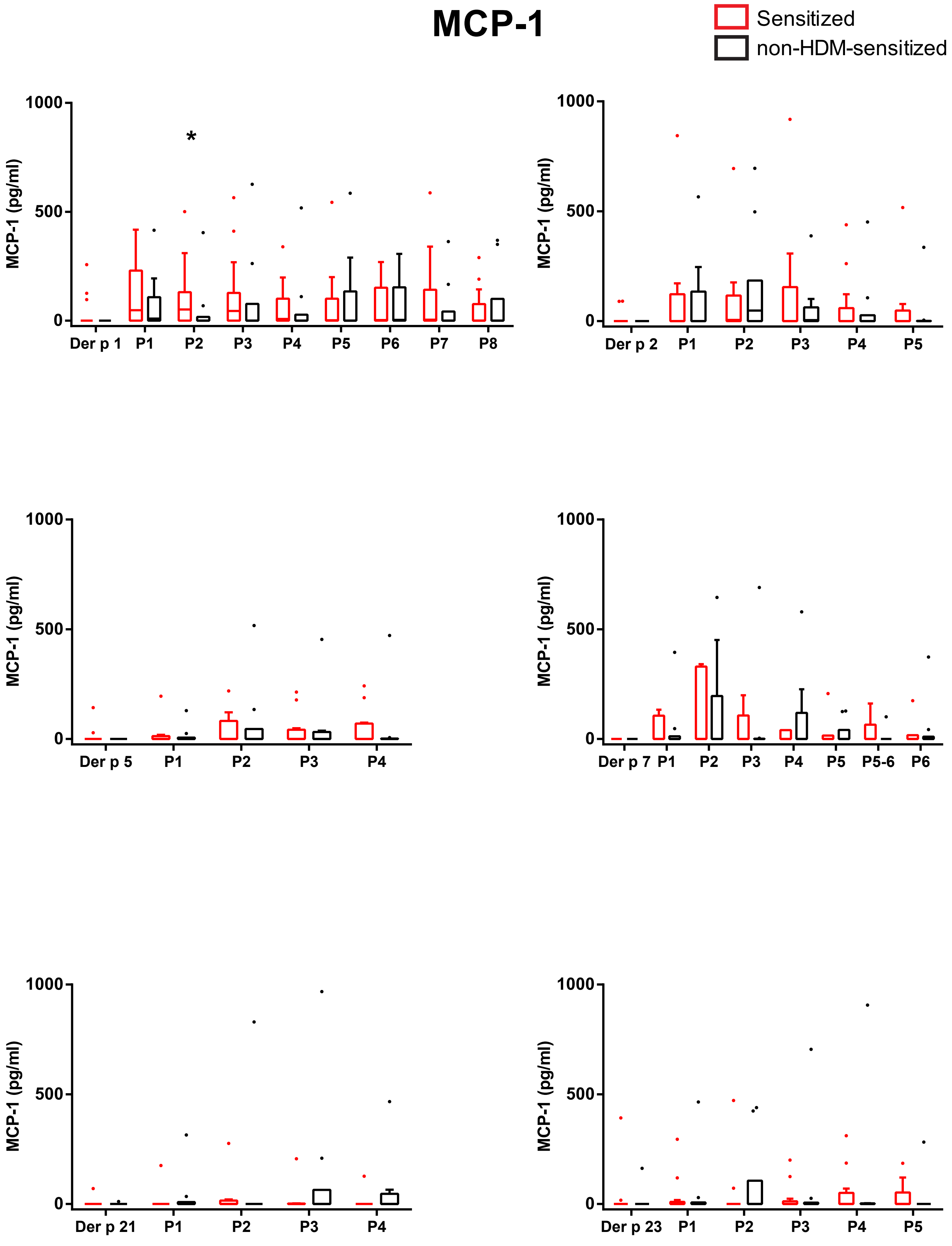

MCP-1
Sensitized
non-HDM-sensitized
MCP-1 (pg/ml)
1000
500
0
*
Der p 1
P1
P2
P3
P4
P5
P6
P7
P8
Der p 2
Der p 5
Der p 7
P5-6
Der p 21
Der p 23

Figure S2E.

MIP 1-beta

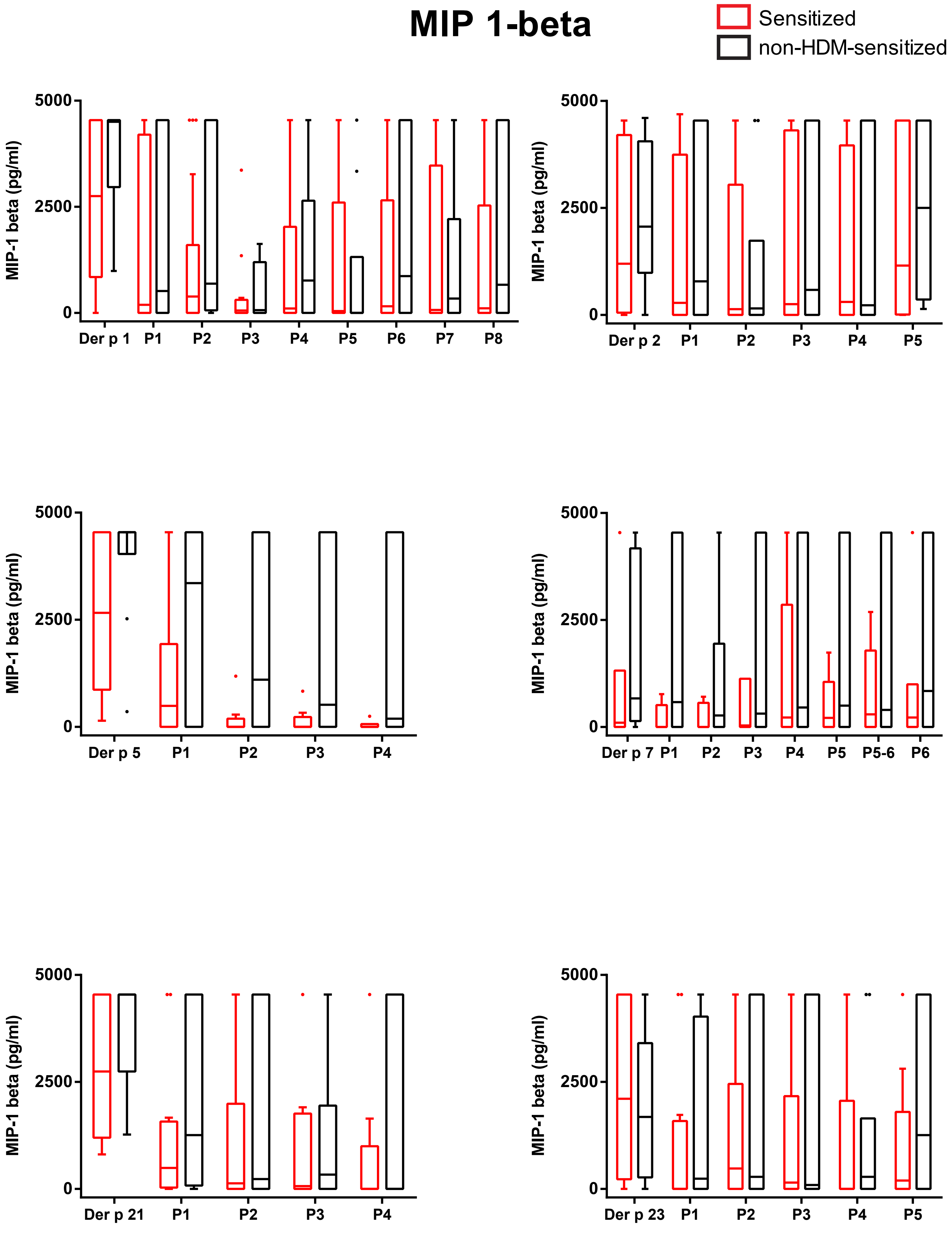

Figure S2F.

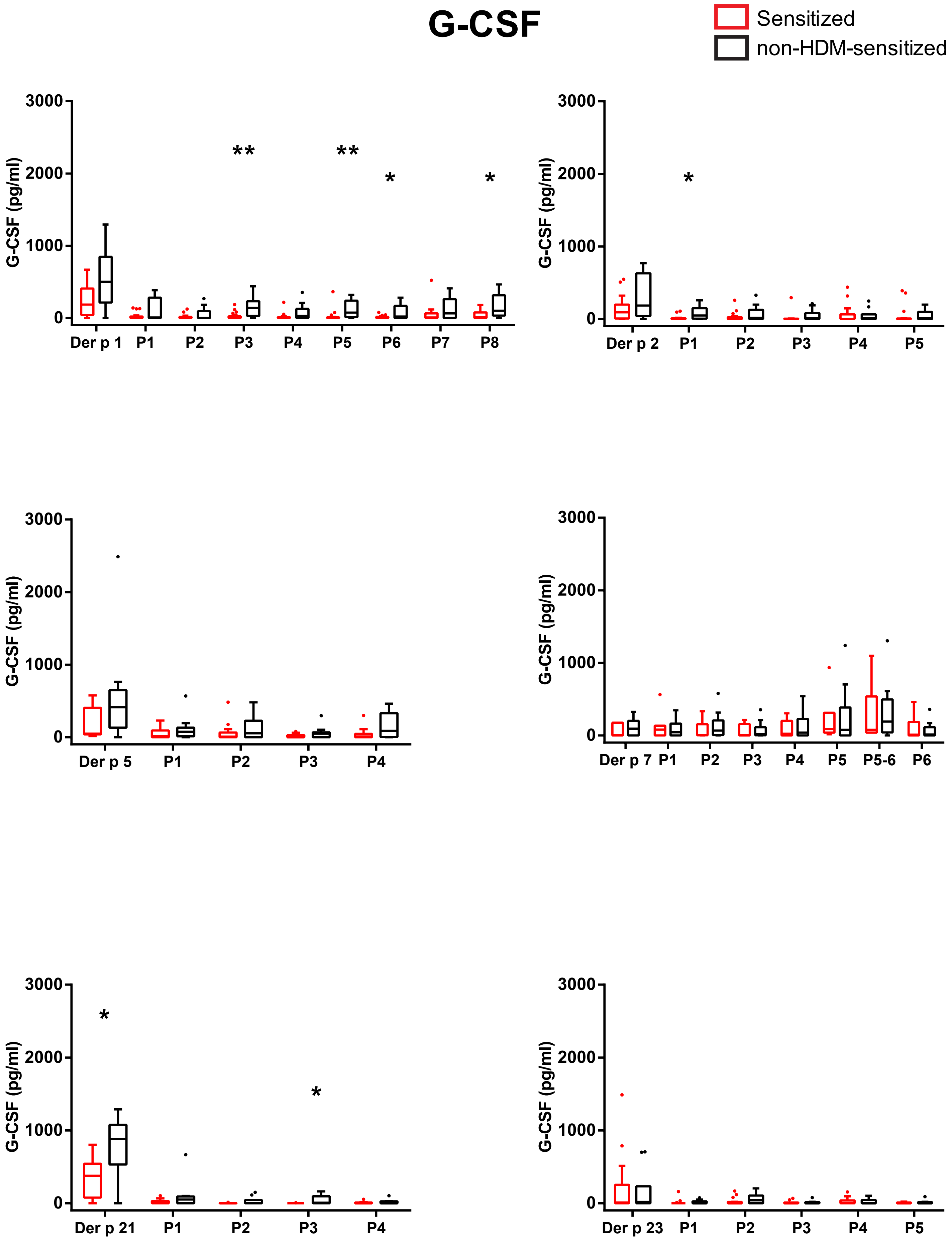
G-CSF
Sensitized
non-HDM-sensitized
G-CSF (pg/ml)
3000
2000
1000
0
**
**
*
*
Der p 1
P1
P2
P3
P4
P5
P6
P7
P8
*
Der p 2
P1
P2
P3
P4
P5
Der p 5
P1
P2
P3
P4
Der p 7
P1
P2
P3
P4
P5
P5-6
P6
*
*
Der p 21
P1
P2
P3
P4
Der p 23
P1
P2
P3
P4
P5

Figure S2G.
