# Supplementary material for: A hypoallergenic peptide mix containing T cell epitopes of the clinically relevant house dust mite allergens
Source: Allergy. 2019 Oct 3;74(12):2461–78. doi: 10.1111/all.13956 (PMC7078969; doi:10.1111/all.13956)
Supplement: Supplementary file 3 [file ALL-74-2461-s003.pdf]

Table S1. Characteristics of allergen-derived peptides

	No.	Sequence (N'-C')	No. of aa	Molecular weight (Da)	Isoelectric point
Der p 1	1	TNACSINGNAPAEIDLRQMRTVTPIRMQGGCGSCWAFSGVA	41	4284.8	7.65
	2	ATESAYLAYRNQSLDLAEQELVDCASQHGCHGDTIPRGIEYIQ	43	4767.1	4.43
	3	HNGVVQESYYRYVAREQSCRRPNAQRFGISN	31	3686.0	9.77
	4	REQSCRRPNAQRFGISNYCQIYPPNVNKIREALAQTH	37	4359.9	9.84
	5	KDLDAFRHYDGRTIIQRDNGYQPNYHAVNIV	31	3690.0	6.92
	6	GRTIIQRDNGYQPNYHAVNIVGYSNAQGVDYWI	33	3783.1	6.74
	7	VGYSNAQGVDYWIVRNSWDTNWGDNGYGYFAANI	34	3874.1	3.93
	8	VRNSWDTNWGDNGYGYFAANIDIMMIEEYPYVVIL	35	4130.6	3.77
Der p 2	1	DQVDVKDCANHEIKKVLVPGCHGSEPCIIHRGK	33	3626.1	7.00
	2	CHGSEPCIIHRGKPFQLEAVFEANQNSKTAK	31	3440.9	8.05
	3	EANQNSKTAKIEIKASIEGLEVDVPGIDPNAC	32	3354.7	4.36
	4	EVDVPGIDPNACHYMKCPLVKGQQYDIKYTWIVPKIAPKSEN	42	4789.5	6.83
	5	APKSENVVVTVKVMGDNGVLACAIATHAKIRD	32	3307.8	8.23
Der p 5	1	CDYQNEFDFLLMERIHEQIKKGELALFYLQ	30	3735.2	4.64
	2	CKKGELALFYLQEQINHFEEKPTKEMKDKIVAEMDTI	37	4399.1	5.16
	3	CDGVRGVLDRLMQRKDLDIFEQYNLEMAKKSG	32	3729.3	6.26
	4	CEQYNLEMAKKSGDILERDLKKEEARVKKIEV	32	3794.4	6.40
Der p 7	1	CDPIHYDKITEEINKAVDEAVAAIEKSETFD	31	3494.8	4.20
	2	CVAAIEKSETFDPMKVPDHSDKFERHIGIIDL	32	3642.1	4.96
	3	CLKGELDMRNIQVRGLKQMKRVGDANVKSEDG	32	3589.1	9.11
	4	CVHDDVVSMEYDLAYKLGDLHPNTHVISDIQDFVVEL	37	4230.7	4.18
	5	CVELSLEVSEEGNMTLTSFEVRQFANV	27	3032.3	3.98
	5-6	CVNHIGGLSILDPIFAVLSDVLTAIFQDT	29	3072.5	3.93
	6	CTAIFQDTVRAEMTKVLAPAFKKELERNNQ	30	3452.9	8.18
Der p 21	1	CFIVGDKKEDEWRMAFDRLMMEELETKIDQVEKGL	35	4233.9	4.50
	2	CLHLSEQYKELEKTKSKELKEQILRELTIGENFMKGAL	38	4479.2	6.78
	3	CMKGALKFFEMEAKRTDLNMFERYNYEFAL	30	3697.3	6.30
	4	CYNYEFALESIKLLIKKLDELAKKVKAVNPDEYY	34	4052.7	6.32
Der p 23	1	MANDNDDDPTTTVHPTTTEQPDDKFECPSRFG	32	3582.7	3.98
	2	PTTTEQPDDKFECPSRFGYFADPKDPHKFYICSN	34	3982.3	4.89
	3	GYFADPKDPHKFYICSNWEAVHKDCPGNTRWNEDEETCT	39	4604.9	4.74
	4	GYFADPKDPHKFYICSNWEAVHKDCPGNT	29	3340.6	6.01
	5	KFYICSNWEAVHKDCPGNTRWNEDEETCT	29	3476.7	4.64

Cysteines added to the N'-terminus to facilitate coupling to carriers are underlined.
