# Supplementary material for: A hypoallergenic peptide mix containing T cell epitopes of the clinically relevant house dust mite allergens
Source: Allergy. 2019 Oct 3;74(12):2461–78. doi: 10.1111/all.13956 (PMC7078969; doi:10.1111/all.13956)
Supplement: Supplementary file 4 [file ALL-74-2461-s004.pdf]

Table S2. Demographic and clinical characteristics of 27 HDM-allergic patients, 5 non-HDM-sensitized allergic patients and 5 non-allergic individuals

Patient no	Age (y)/sex†	HDM-related symptoms‡	Family history of allergy	Current treatment§	Total IgE (kU/l)	Der p-specific IgE (kUA/l)
PA1	33/F	R	No	No	252.0	19.0
PA2	24/F	R	NK	No	75.5	8.5
PA3	31/M	R	Yes	No	44.0	8.2
PA4	24/M	R	NK	No	42.9	8.7
PA5	26/F	A, R	Yes	AH	139.0	27.6
PA6	27/F	R	Yes	No	90.7	11.4
PA7	29/M	R, C	Yes	No	1349.0	56.8
PA8	22/F	R	NK	NK	509.0	31.3
PA9	25/M	R	NK	NK	994.0	27.0
PA10	21/F	R	Yes	tSt	1714.0	50.8
PA11	26/F	A, R	No	tSt	491.0	56.1
PA12	25/F	A, R	No	tSt	702.0	4.7
PA13	22/F	R	Yes	AH	538.0	38.9
PA14	23/F	R	Yes	No	61.3	8.0
PA15	35/F	R	Yes	No	350.0	29.6
PA16	23/M	R	Yes	AH	164.0	14.0
PA17	26/M	R	Yes	AH	77.9	5.9
PA18	19/F	R	Yes	AH, tSt	710.0	12.8
PA19	27/M	R	Yes	No	1595.0	101.0
PA20	27/F	A, R	No	tSt	105.0	30.5
PA21	27/M	R, C	No	tSt	99.5	27.3
PA22	31/F	R	Yes	AH	440.0	0.7
PA23	24/F	AD, R	Yes	AH, tSt	1260.0	117.0
PA24	36/F	R, C	NK	AH	3425.0	62.5
PA25	35/F	R	Yes	No	55.2	5.0
PA26	22/F	R	No	AH	219.0	15.7
PA27	25/M	R	NK	NK	61.6	3.3

Donor no	Age (y)/sex†	Allergic sensitization	Family history of allergy	Current treatment§	Total IgE (kU/l)
¶NDP1	28/M	cashew, honey bee, peanut, chicken, soybean, latex, timothy, london plane tree, wheat	No	No	65.0
NDP2	23/F	kiwi, alder, celery, peanut, birch pollen, dog, hazelnut, sugi, bermuda grass, cypress, horse, cat, apple, mouse, olive, timothy, peach	Yes	AH, tSt	951.0
NDP3	55/F	dog, cat	No	No	118.0
NDP4	29/M	*Alternaria* , cat	Yes	tSt	20.3
NDP5	24/M	alder, short ragweed, cashew, celery, peanut, birch pollen, hazelnut, bermuda grass, soybean, latex, apple, mercury, timothy, pistachio, almond, wasp	No	AH	248.0
††NA1	31/F	No	Yes	No	28.6
NA2	35/M	Honey bee, soybean, wheat	No	No	70.0
NA3	31/F	No	No	No	4.2
NA4	24/F	wasp	Yes	No	26.2
NA5	36/F	No	No	No	14.9

Abbreviation: NK, not known

† M, male; F, female

‡ A, bronchial asthma; AD, atopic dermatitis; R, rhinitis; C, conjunctivitis

§ AH, antihistamine; tSt, topical steroids

¶ NDP1-5: non-HDM-sensitized allergic individuals

†† NA1-5: non-allergic individuals
