# Supplementary material for: A hypoallergenic peptide mix containing T cell epitopes of the clinically relevant house dust mite allergens
Source: Allergy. 2019 Oct 3;74(12):2461–78. doi: 10.1111/all.13956 (PMC7078969; doi:10.1111/all.13956)
Supplement: Supplementary file 5 [file ALL-74-2461-s005.pdf]

Table S3. IgE reactivity of HDM-allergic patients (PA), non-HDM-sensitized allergic patients (NDP) and non-allergic individuals (NA) to micro-arrayed allergens determined by ImmunoCAP ISAC technology.

Patient no. PA1							
Act d 1	0	Bla g 5	0	V Der p 14	0	Phl p 2	0
Act d 2	0	Bla g 7	0	V Der p 15	0	Phl p 4	0
Act d 5	0	Blo t 5	0	V Der p 18	0	Phl p 5	0
Act d 8	0	Bos d 4	0	V Der p 21	0.64	Phl p 6	0
Aln g 1	0	V Bos d 4	0	V Der p 23	1.43	Phl p 7	0
Alt a 1	0	Bos d 5	0	V clone 16	0	Phl p 11	0
Alt a 6	0	V Bos d 5	0	Equ c 1	0	Phl p 12	0
Amb a 1	0	Bos d Lactoferrin	0	Equ c 3	0	V Pis v 3	0
V Ana o 1	0	Bos d 8	0	Fag e 2	0	Pla a 1	0
Ana o 2	0	V Bos d 8	0	Fel d 1	1.89	Pla a 2	0
V Ana o 2	0	aS1-casein	0	Fel d 2	0	Pla a 3	0
V nAna o 2	0	aS2-casein	0	Fel d 4	0	Pla l 1	0
V Ana o 3	0	b-casein	0	Gad c 1	0	Pol d 5	0
Ani s 1	0	K-casein	0	Gal d 1	0	Pru p 1	0
Ani s 3	0	Transferrin	0	Gal d 2	0	Pru p 3	0
Api g 1	0	Bos d 6	0	Gal d 3	0	V Pru du 3	0
Api m 1	0	V BSA	0	Gal d 5	0	V Pru du 4	0
V Api m 1	0	Can f 1	0	Gly m 4	0	V Pru du 6	0
V Api m 2	0	Can f 2	0	Gly m 5	0	V Pru du 6.01	0
Api m 4	0	Can f 3	0	Gly m 6	0	V Pru du 6.02	0
V Api m 4	0	V Can f 4	0	Hev b 1	0	Sal k 1	0
Ara h 1	0	Can f 5	0	Hev b 3	0	Ses i 1	0
V Ara h 1	0	V Can f 5	0	Hev b 5	0	Tri a 14	0
Ara h 2	0	V Can f 6	0	Hev b 6.01	0	Tri a 19.0101	0
Ara h 3	0	Che a 1	0	Hev b 8	0	Tri a aA_TI	0
V Ara h 3	0	Cla h 8	0	Jug r 1	0	V Tri a 36 191	0
Ara h 6	0	Cor a 1.0401	0	Jug r 2	0.07	V Tri a 36	0
V Ara h 6	0	Cor a 8	0	Jug r 3	0	V m43	0
Ara h 8	0	Cor a 9	0	Lep d 2	0	V m82	0
Ara h 9	0	Cry j 1	0	Mal d 1	0	Serine	0
Art v 1	0	Cyn d 1	0.09	Mer a 1	0	Thioredoxin	0
Art v 3	0	Cup a 1	0	Mus m 1	0	Glutathione	0
Asp f 1	0	Der f 1	1.34	MUXF3	0	peroxiredoxin	0
Asp f 3	0	Der f 2	7.12	Ole e 1	0	Profilin	0
Asp f 6	0	Der p 1	2.38	Ole e 7	0	Dehydrin	0
Ber e 1	0	Der p 2	6.47	Ole e 9	0	purothionin	0
Bet v 1	0	V Der p 4	0	Par j 2	0	LTP	0
Bet v 2	0	V Der p 5	0	Pen m 1	0	V Ves v 1	0
Bet v 4	0	V Der p 7	0	Pen m 2	0	Ves v 5	0.08
Bla g 1	0	Der p 10	0.24	Pen m 4	0	V Ves v 5	0
Bla g 2	0	V Der p 11	0	Phl p 1	0.44	Blank	0

IgE levels are shown in ISAC Standardized Units (ISU), cut-off = 0.3 ISU.

Patient no. PA2							
Act d 1	0	Bla g 5	0	V Der p 14	0	Phl p 2	0
Act d 2	0	Bla g 7	0	V Der p 15	0	Phl p 4	0
Act d 5	0	Blo t 5	0	V Der p 18	0	Phl p 5	0
Act d 8	0	Bos d 4	0	V Der p 21	0	Phl p 6	0
Aln g 1	0	V Bos d 4	0	V Der p 23	0.34	Phl p 7	0
Alt a 1	0	Bos d 5	0	V clone 16	0	Phl p 11	0
Alt a 6	0	V Bos d 5	0	Equ c 1	0	Phl p 12	0
Amb a 1	0	Bos d Lactoferrin	0	Equ c 3	0	V Pis v 3	0
V Ana o 1	0	Bos d 8	0	Fag e 2	0	Pla a 1	0
Ana o 2	0	V Bos d 8	0	Fel d 1	0	Pla a 2	0
V Ana o 2	0	aS1-casein	0	Fel d 2	0	Pla a 3	0
V nAna o 2	0	aS2-casein	0	Fel d 4	0	Pla l 1	0
V Ana o 3	0	b-casein	0	Gad c 1	0	Pol d 5	0
Ani s 1	0	K-casein	0	Gal d 1	0	Pru p 1	0
Ani s 3	0	Transferrin	0	Gal d 2	0	Pru p 3	0
Api g 1	0	Bos d 6	0	Gal d 3	0	V Pru du 3	0
Api m 1	0	V BSA	0	Gal d 5	0	V Pru du 4	0
V Api m 1	0	Can f 1	0	Gly m 4	0	V Pru du 6	0
V Api m 2	0	Can f 2	0	Gly m 5	0	V Pru du 6.01	0
Api m 4	0	Can f 3	0	Gly m 6	0	V Pru du 6.02	0
V Api m 4	0	V Can f 4	0	Hev b 1	0	Sal k 1	0
Ara h 1	0	Can f 5	0	Hev b 3	0	Ses i 1	0
V Ara h 1	0	V Can f 5	0	Hev b 5	0	Tri a 14	0
Ara h 2	0	V Can f 6	0	Hev b 6.01	0	Tri a 19.0101	0
Ara h 3	0	Che a 1	0	Hev b 8	0	Tri a aA_TI	0
V Ara h 3	0	Cla h 8	0	Jug r 1	0	V Tri a 36 191	0
Ara h 6	0	Cor a 1.0401	0	Jug r 2	0	V Tri a 36	0
V Ara h 6	0	Cor a 8	0	Jug r 3	0	V m43	0
Ara h 8	0	Cor a 9	0	Lep d 2	0	V m82	0
Ara h 9	0	Cry j 1	0	Mal d 1	0.14	Serine	0
Art v 1	0	Cyn d 1	0	Mer a 1	0	Thioredoxin	0
Art v 3	0	Cup a 1	0	Mus m 1	0	Glutathione	0
Asp f 1	0	Der f 1	0.86	MUXF3	0	peroxiredoxin	0
Asp f 3	0	Der f 2	0.18	Ole e 1	0.08	Profilin	0
Asp f 6	0	Der p 1	1.19	Ole e 7	0	Dehydrin	0
Ber e 1	0	Der p 2	0.26	Ole e 9	0	purothionin	0
Bet v 1	0.25	V Der p 4	0	Par j 2	0	LTP	0
Bet v 2	0	V Der p 5	1.31	Pen m 1	0	V Ves v 1	0
Bet v 4	0	V Der p 7	0	Pen m 2	0	Ves v 5	0.11
Bla g 1	0	Der p 10	0.14	Pen m 4	0	V Ves v 5	0
Bla g 2	0	V Der p 11	0	Phl p 1	0	Blank	0

Patient no. PA3							
Act d 1	0	Bla g 5	0	V Der p 14	0	Phl p 2	0
Act d 2	0	Bla g 7	0	V Der p 15	0	Phl p 4	0
Act d 5	0	Blo t 5	0	V Der p 18	0	Phl p 5	0
Act d 8	0	Bos d 4	0	V Der p 21	0	Phl p 6	0
Aln g 1	0	V Bos d 4	0	V Der p 23	0	Phl p 7	0
Alt a 1	0	Bos d 5	0	V clone 16	0	Phl p 11	0
Alt a 6	0	V Bos d 5	0	Equ c 1	0	Phl p 12	0
Amb a 1	0	Bos d Lactoferrin	0	Equ c 3	0	V Pis v 3	0
V Ana o 1	0	Bos d 8	0	Fag e 2	0	Pla a 1	0
Ana o 2	0.27	V Bos d 8	0	Fel d 1	0.85	Pla a 2	0
V Ana o 2	0	aS1-casein	0	Fel d 2	0	Pla a 3	0
V nAna o 2	0	aS2-casein	0	Fel d 4	0	Pla l 1	0
V Ana o 3	0	b-casein	0	Gad c 1	0	Pol d 5	0
Ani s 1	0	K-casein	0	Gal d 1	0	Pru p 1	0
Ani s 3	0	Transferrin	0	Gal d 2	0	Pru p 3	0
Api g 1	0	Bos d 6	0	Gal d 3	0	V Pru du 3	0
Api m 1	0	V BSA	0	Gal d 5	0	V Pru du 4	0
V Api m 1	0	Can f 1	0	Gly m 4	0	V Pru du 6	0
V Api m 2	0	Can f 2	0	Gly m 5	0.28	V Pru du 6.01	0
Api m 4	0	Can f 3	0	Gly m 6	0	V Pru du 6.02	0
V Api m 4	0	V Can f 4	0	Hev b 1	0.34	Sal k 1	0
Ara h 1	0	Can f 5	0	Hev b 3	0	Ses i 1	0
V Ara h 1	0	V Can f 5	0	Hev b 5	0	Tri a 14	0
Ara h 2	0	V Can f 6	0	Hev b 6.01	0	Tri a 19.0101	0.79
Ara h 3	0	Che a 1	0	Hev b 8	0	Tri a aA_TI	0
V Ara h 3	0	Cla h 8	0	Jug r 1	0	V Tri a 36 191	0
Ara h 6	0	Cor a 1.0401	0	Jug r 2	0	V Tri a 36	0
V Ara h 6	0	Cor a 8	0	Jug r 3	0	V m43	0
Ara h 8	0	Cor a 9	0	Lep d 2	0	V m82	0
Ara h 9	0	Cry j 1	0	Mal d 1	0	Serine	0
Art v 1	0	Cyn d 1	0	Mer a 1	0	Thioredoxin	0
Art v 3	0	Cup a 1	0	Mus m 1	0	Glutathione	0
Asp f 1	0	Der f 1	0	MUXF3	0	peroxiredoxin	0
Asp f 3	0	Der f 2	22.21	Ole e 1	0	Profilin	0
Asp f 6	0	Der p 1	0	Ole e 7	0	Dehydrin	0
Ber e 1	0	Der p 2	9.38	Ole e 9	0	purothionin	0
Bet v 1	0	V Der p 4	0	Par j 2	0	LTP	0
Bet v 2	0	V Der p 5	0	Pen m 1	0	V Ves v 1	0
Bet v 4	0	V Der p 7	0	Pen m 2	0	Ves v 5	0
Bla g 1	0	Der p 10	0	Pen m 4	0	V Ves v 5	0
Bla g 2	0	V Der p 11	0	Phl p 1	0.46	Blank	0

Patient no. PA4							
Act d 1	0	Bla g 5	0	V Der p 14	0	Phl p 2	0
Act d 2	0	Bla g 7	0	V Der p 15	0	Phl p 4	0
Act d 5	0	Blo t 5	0	V Der p 18	0	Phl p 5	0
Act d 8	0	Bos d 4	0	V Der p 21	0	Phl p 6	0
Aln g 1	0	V Bos d 4	0	V Der p 23	0	Phl p 7	0
Alt a 1	0	Bos d 5	0	V clone 16	0	Phl p 11	0
Alt a 6	0	V Bos d 5	0	Equ c 1	0	Phl p 12	0
Amb a 1	0	Bos d Lactoferrin	0	Equ c 3	0	V Pis v 3	0
V Ana o 1	0	Bos d 8	0	Fag e 2	0	Pla a 1	0
Ana o 2	0	V Bos d 8	0	Fel d 1	0	Pla a 2	0
V Ana o 2	0	aS1-casein	0	Fel d 2	0	Pla a 3	0
V nAna o 2	0	aS2-casein	0	Fel d 4	0	Pla l 1	0
V Ana o 3	0	b-casein	0	Gad c 1	0	Pol d 5	0
Ani s 1	0	K-casein	0	Gal d 1	0	Pru p 1	0
Ani s 3	0	Transferrin	0	Gal d 2	0	Pru p 3	0
Api g 1	0	Bos d 6	0	Gal d 3	0	V Pru du 3	0
Api m 1	0	V BSA	0	Gal d 5	0	V Pru du 4	0
V Api m 1	0	Can f 1	0	Gly m 4	0	V Pru du 6	0
V Api m 2	0	Can f 2	0	Gly m 5	0	V Pru du 6.01	0
Api m 4	0	Can f 3	0	Gly m 6	0	V Pru du 6.02	0
V Api m 4	0	V Can f 4	0	Hev b 1	0	Sal k 1	0
Ara h 1	0	Can f 5	0	Hev b 3	0	Ses i 1	0
V Ara h 1	0	V Can f 5	0	Hev b 5	0	Tri a 14	0
Ara h 2	0	V Can f 6	0	Hev b 6.01	0	Tri a 19.0101	0
Ara h 3	0	Che a 1	0	Hev b 8	0	Tri a aA_TI	0
V Ara h 3	0	Cla h 8	0	Jug r 1	0	V Tri a 36 191	0
Ara h 6	0	Cor a 1.0401	0	Jug r 2	0	V Tri a 36	0
V Ara h 6	0	Cor a 8	0	Jug r 3	0	V m43	0
Ara h 8	0	Cor a 9	0	Lep d 2	0	V m82	0
Ara h 9	0	Cry j 1	0	Mal d 1	0	Serine	0
Art v 1	0	Cyn d 1	0	Mer a 1	0	Thioredoxin	0
Art v 3	0	Cup a 1	0	Mus m 1	0	Glutathione	0
Asp f 1	0	Der f 1	0.18	MUXF3	0	peroxiredoxin	0
Asp f 3	0	Der f 2	2.31	Ole e 1	0	Profilin	0
Asp f 6	0	Der p 1	0.29	Ole e 7	0	Dehydrin	0
Ber e 1	0	Der p 2	1.46	Ole e 9	0	purothionin	0
Bet v 1	0	V Der p 4	0	Par j 2	0	LTP	0
Bet v 2	0	V Der p 5	0	Pen m 1	0	V Ves v 1	0
Bet v 4	0	V Der p 7	0	Pen m 2	0	Ves v 5	0
Bla g 1	0	Der p 10	0	Pen m 4	0	V Ves v 5	0
Bla g 2	0	V Der p 11	0	Phl p 1	0	Blank	0

Patient no. PA5							
Act d 1	0	Bla g 5	0	V Der p 14	0	Phl p 2	0
Act d 2	0	Bla g 7	0	V Der p 15	0	Phl p 4	0
Act d 5	0	Blo t 5	0	V Der p 18	0	Phl p 5	0.99
Act d 8	0	Bos d 4	0	V Der p 21	0	Phl p 6	0
Aln g 1	0	V Bos d 4	0	V Der p 23	0	Phl p 7	0
Alt a 1	0.31	Bos d 5	0	V clone 16	0	Phl p 11	0
Alt a 6	0	V Bos d 5	0	Equ c 1	0	Phl p 12	0
Amb a 1	0	Bos d Lactoferrin	0	Equ c 3	0	V Pis v 3	0
V Ana o 1	0	Bos d 8	0	Fag e 2	0	Pla a 1	0
Ana o 2	0	V Bos d 8	0	Fel d 1	0	Pla a 2	0
V Ana o 2	0	aS1-casein	0	Fel d 2	0	Pla a 3	0
V nAna o 2	0	aS2-casein	0	Fel d 4	0	Pla l 1	0
V Ana o 3	0	b-casein	0	Gad c 1	0	Pol d 5	0
Ani s 1	0	K-casein	0	Gal d 1	0	Pru p 1	0
Ani s 3	0	Transferrin	0	Gal d 2	0	Pru p 3	0
Api g 1	0	Bos d 6	0	Gal d 3	0	V Pru du 3	0
Api m 1	0	V BSA	0	Gal d 5	0	V Pru du 4	0
V Api m 1	0	Can f 1	1.37	Gly m 4	0	V Pru du 6	0
V Api m 2	0	Can f 2	0	Gly m 5	0	V Pru du 6.01	0
Api m 4	0	Can f 3	0	Gly m 6	0	V Pru du 6.02	0
V Api m 4	0	V Can f 4	0	Hev b 1	0	Sal k 1	0
Ara h 1	0	Can f 5	0	Hev b 3	0	Ses i 1	0
V Ara h 1	0	V Can f 5	0	Hev b 5	0	Tri a 14	0
Ara h 2	0	V Can f 6	0	Hev b 6.01	0	Tri a 19.0101	0
Ara h 3	0	Che a 1	0	Hev b 8	0	Tri a aA_TI	0
V Ara h 3	0	Cla h 8	0	Jug r 1	0	V Tri a 36 191	0
Ara h 6	0	Cor a 1.0401	0	Jug r 2	0	V Tri a 36	0
V Ara h 6	0	Cor a 8	0	Jug r 3	0	V m43	0
Ara h 8	0	Cor a 9	0	Lep d 2	0	V m82	0
Ara h 9	0	Cry j 1	0	Mal d 1	0	Serine	0
Art v 1	0	Cyn d 1	0	Mer a 1	0	Thioredoxin	0
Art v 3	0	Cup a 1	0	Mus m 1	0	Glutathione	0
Asp f 1	0	Der f 1	5.58	MUXF3	0	peroxiredoxin	0
Asp f 3	0	Der f 2	25.64	Ole e 1	0.36	Profilin	0
Asp f 6	0	Der p 1	9.15	Ole e 7	0	Dehydrin	0
Ber e 1	0	Der p 2	16.61	Ole e 9	0	purothionin	0
Bet v 1	0	V Der p 4	0	Par j 2	0	LTP	0
Bet v 2	0	V Der p 5	0.22	Pen m 1	0	V Ves v 1	0
Bet v 4	0	V Der p 7	0.08	Pen m 2	0	Ves v 5	0
Bla g 1	0	Der p 10	0.27	Pen m 4	0	V Ves v 5	0
Bla g 2	0	V Der p 11	0	Phl p 1	0	Blank	0

Patient no. PA6							
Act d 1	0	Bla g 5	0	V Der p 14	0	Phl p 2	0
Act d 2	0	Bla g 7	0	V Der p 15	0	Phl p 4	0
Act d 5	0	Blo t 5	0.57	V Der p 18	0	Phl p 5	0
Act d 8	0	Bos d 4	0	V Der p 21	1.44	Phl p 6	0
Aln g 1	0	V Bos d 4	0	V Der p 23	2.84	Phl p 7	0
Alt a 1	0	Bos d 5	0	V clone 16	0	Phl p 11	0
Alt a 6	0	V Bos d 5	0	Equ c 1	0	Phl p 12	0
Amb a 1	0	Bos d Lactoferrin	0	Equ c 3	0	V Pis v 3	0
V Ana o 1	0	Bos d 8	0	Fag e 2	0	Pla a 1	0
Ana o 2	0	V Bos d 8	0	Fel d 1	0	Pla a 2	0
V Ana o 2	0	aS1-casein	0	Fel d 2	0	Pla a 3	0
V nAna o 2	0	aS2-casein	0	Fel d 4	0	Pla l 1	0
V Ana o 3	0	b-casein	0	Gad c 1	0	Pol d 5	0
Ani s 1	0	K-casein	0	Gal d 1	0	Pru p 1	0
Ani s 3	0	Transferrin	0	Gal d 2	0	Pru p 3	0
Api g 1	0	Bos d 6	0	Gal d 3	0	V Pru du 3	0
Api m 1	0	V BSA	0	Gal d 5	0	V Pru du 4	0
V Api m 1	0	Can f 1	0	Gly m 4	0	V Pru du 6	0
V Api m 2	0	Can f 2	0	Gly m 5	0	V Pru du 6.01	0
Api m 4	0	Can f 3	0	Gly m 6	0	V Pru du 6.02	0
V Api m 4	0	V Can f 4	0	Hev b 1	0	Sal k 1	0
Ara h 1	0	Can f 5	0	Hev b 3	0	Ses i 1	0
V Ara h 1	0	V Can f 5	0	Hev b 5	0	Tri a 14	0
Ara h 2	0	V Can f 6	0	Hev b 6.01	0	Tri a 19.0101	0
Ara h 3	0	Che a 1	0	Hev b 8	0	Tri a aA_TI	0
V Ara h 3	0	Cla h 8	0	Jug r 1	0	V Tri a 36 191	0
Ara h 6	0	Cor a 1.0401	0	Jug r 2	0	V Tri a 36	0
V Ara h 6	0	Cor a 8	0	Jug r 3	0	V m43	0
Ara h 8	0	Cor a 9	0	Lep d 2	0	V m82	0
Ara h 9	0	Cry j 1	0	Mal d 1	0	Serine	0
Art v 1	0	Cyn d 1	0	Mer a 1	0	Thioredoxin	0
Art v 3	0	Cup a 1	0	Mus m 1	0	Glutathione	0
Asp f 1	0	Der f 1	1	MUXF3	0	peroxiredoxin	0
Asp f 3	0	Der f 2	3.99	Ole e 1	0	Profilin	0
Asp f 6	0	Der p 1	0.71	Ole e 7	0	Dehydrin	0
Ber e 1	0	Der p 2	3.25	Ole e 9	0	purothionin	0
Bet v 1	0	V Der p 4	0	Par j 2	0	LTP	0
Bet v 2	0	V Der p 5	1.73	Pen m 1	0	V Ves v 1	0
Bet v 4	0	V Der p 7	0.07	Pen m 2	0	Ves v 5	0
Bla g 1	0	Der p 10	0.09	Pen m 4	0	V Ves v 5	0
Bla g 2	0	V Der p 11	0	Phl p 1	0	Blank	0

Patient no. PA7							
Act d 1	0	Bla g 5	0	V Der p 14	0	Phl p 2	1.23
Act d 2	0	Bla g 7	0	V Der p 15	0	Phl p 4	0.78
Act d 5	0	Blo t 5	0	V Der p 18	0	Phl p 5	17.3
Act d 8	0	Bos d 4	0	V Der p 21	0	Phl p 6	1.42
Aln g 1	1.06	V Bos d 4	0	V Der p 23	7.86	Phl p 7	0
Alt a 1	0	Bos d 5	0	V clone 16	0	Phl p 11	1.92
Alt a 6	0	V Bos d 5	0	Equ c 1	17.9	Phl p 12	0
Amb a 1	0	Bos d Lactoferrin	0	Equ c 3	0.17	V Pis v 3	0
V Ana o 1	0	Bos d 8	0	Fag e 2	0	Pla a 1	0
Ana o 2	0	V Bos d 8	0	Fel d 1	9.68	Pla a 2	0.2
V Ana o 2	0	aS1-casein	0	Fel d 2	0	Pla a 3	0
V nAna o 2	0	aS2-casein	0	Fel d 4	9.16	Pla l 1	0
V Ana o 3	0	b-casein	0	Gad c 1	0	Pol d 5	0
Ani s 1	0	K-casein	0	Gal d 1	0	Pru p 1	0.96
Ani s 3	0	Transferrin	0	Gal d 2	0	Pru p 3	0.1
Api g 1	0.07	Bos d 6	0	Gal d 3	0	V Pru du 3	0
Api m 1	0	V BSA	0	Gal d 5	0	V Pru du 4	0
V Api m 1	0	Can f 1	25.82	Gly m 4	0.08	V Pru du 6	0
V Api m 2	0	Can f 2	3.79	Gly m 5	0	V Pru du 6.01	0
Api m 4	0	Can f 3	0	Gly m 6	0	V Pru du 6.02	0
V Api m 4	0	V Can f 4	0	Hev b 1	0	Sal k 1	0
Ara h 1	0	Can f 5	0	Hev b 3	0	Ses i 1	0
V Ara h 1	0.07	V Can f 5	0	Hev b 5	0	Tri a 14	0
Ara h 2	0	V Can f 6	0	Hev b 6.01	0	Tri a 19.0101	0
Ara h 3	0	Che a 1	0.12	Hev b 8	0	Tri a aA_TI	0
V Ara h 3	0	Cla h 8	0	Jug r 1	0	V Tri a 36 191	0
Ara h 6	0	Cor a 1.0401	0.17	Jug r 2	0.33	V Tri a 36	0
V Ara h 6	0	Cor a 8	0.08	Jug r 3	0	V m43	0
Ara h 8	0.71	Cor a 9	0	Lep d 2	0	V m82	0
Ara h 9	0.09	Cry j 1	0.17	Mal d 1	1.41	Serine	0
Art v 1	0	Cyn d 1	3.02	Mer a 1	0	Thioredoxin	0
Art v 3	0.13	Cup a 1	0	Mus m 1	3.26	Glutathione	0
Asp f 1	0	Der f 1	3.87	MUXF3	0	peroxiredoxin	0
Asp f 3	0	Der f 2	25.44	Ole e 1	0.14	Profilin	0
Asp f 6	0	Der p 1	6.76	Ole e 7	0	Dehydrin	0
Ber e 1	0	Der p 2	25.73	Ole e 9	0	purothionin	0
Bet v 1	11.67	V Der p 4	0	Par j 2	0	LTP	0
Bet v 2	0	V Der p 5	0.14	Pen m 1	0	V Ves v 1	0
Bet v 4	0	V Der p 7	0.35	Pen m 2	0.29	Ves v 5	0
Bla g 1	0	Der p 10	0.19	Pen m 4	0	V Ves v 5	0
Bla g 2	0	V Der p 11	0	Phl p 1	0.5	Blank	0

Patient no. PA8							
Act d 1	0	Bla g 5	0	V Der p 14	0	Phl p 2	0
Act d 2	0	Bla g 7	0	V Der p 15	0	Phl p 4	0
Act d 5	0	Blo t 5	0	V Der p 18	1.34	Phl p 5	0
Act d 8	0	Bos d 4	0	V Der p 21	0	Phl p 6	0
Aln g 1	0	V Bos d 4	0	V Der p 23	0	Phl p 7	0
Alt a 1	0	Bos d 5	0	V clone 16	0	Phl p 11	0
Alt a 6	0	V Bos d 5	0	Equ c 1	0	Phl p 12	0
Amb a 1	0	Bos d Lactoferrin	0	Equ c 3	0	V Pis v 3	0
V Ana o 1	0	Bos d 8	0	Fag e 2	0	Pla a 1	0
Ana o 2	0	V Bos d 8	0	Fel d 1	0	Pla a 2	0
V Ana o 2	0	aS1-casein	0	Fel d 2	0	Pla a 3	0
V nAna o 2	0	aS2-casein	0	Fel d 4	0	Pla l 1	0
V Ana o 3	0	b-casein	0	Gad c 1	0	Pol d 5	1.63
Ani s 1	0	K-casein	0	Gal d 1	0	Pru p 1	0
Ani s 3	0	Transferrin	0	Gal d 2	0	Pru p 3	0
Api g 1	0	Bos d 6	0	Gal d 3	0	V Pru du 3	0
Api m 1	0	V BSA	0	Gal d 5	0	V Pru du 4	0
V Api m 1	0	Can f 1	0	Gly m 4	0	V Pru du 6	0
V Api m 2	0	Can f 2	0	Gly m 5	0	V Pru du 6.01	0
Api m 4	0	Can f 3	0	Gly m 6	0	V Pru du 6.02	0
V Api m 4	0	V Can f 4	0	Hev b 1	0	Sal k 1	0
Ara h 1	0	Can f 5	0	Hev b 3	0	Ses i 1	0
V Ara h 1	0	V Can f 5	0	Hev b 5	0	Tri a 14	0
Ara h 2	0	V Can f 6	0	Hev b 6.01	0	Tri a 19.0101	0
Ara h 3	0	Che a 1	0	Hev b 8	0	Tri a aA_TI	0
V Ara h 3	0	Cla h 8	0	Jug r 1	0	V Tri a 36 191	0
Ara h 6	0	Cor a 1.0401	0	Jug r 2	0	V Tri a 36	0
V Ara h 6	0	Cor a 8	0	Jug r 3	0	V m43	0
Ara h 8	0	Cor a 9	0	Lep d 2	0	V m82	0
Ara h 9	0	Cry j 1	0	Mal d 1	0	Serine	0
Art v 1	0	Cyn d 1	0	Mer a 1	0	Thioredoxin	0
Art v 3	0	Cup a 1	0	Mus m 1	0	Glutathione	0
Asp f 1	0	Der f 1	92.36	MUXF3	0	peroxiredoxin	0
Asp f 3	0	Der f 2	38.58	Ole e 1	1.97	Profilin	0
Asp f 6	0	Der p 1	26.18	Ole e 7	0	Dehydrin	0
Ber e 1	0	Der p 2	33.66	Ole e 9	0	purothionin	0
Bet v 1	0	V Der p 4	0.5	Par j 2	0	LTP	0
Bet v 2	0	V Der p 5	0.24	Pen m 1	0	V Ves v 1	0
Bet v 4	0	V Der p 7	0	Pen m 2	0	Ves v 5	2.36
Bla g 1	0	Der p 10	0.11	Pen m 4	0	V Ves v 5	0
Bla g 2	0	V Der p 11	0.13	Phl p 1	0	Blank	0

Patient no. PA9							
Act d 1	0	Bla g 5	0	V Der p 14	0	Phl p 2	0
Act d 2	0	Bla g 7	0	V Der p 15	0	Phl p 4	3.47
Act d 5	0	Blo t 5	20.35	V Der p 18	0.79	Phl p 5	0
Act d 8	0	Bos d 4	0.41	V Der p 21	11.13	Phl p 6	0
Aln g 1	0	V Bos d 4	0	V Der p 23	0	Phl p 7	0
Alt a 1	0	Bos d 5	0	V clone 16	7.46	Phl p 11	0
Alt a 6	0	V Bos d 5	0	Equ c 1	0	Phl p 12	0
Amb a 1	0	Bos d Lactoferrin	0	Equ c 3	0	V Pis v 3	0
V Ana o 1	0	Bos d 8	0	Fag e 2	0	Pla a 1	0
Ana o 2	0	V Bos d 8	0	Fel d 1	0	Pla a 2	1.25
V Ana o 2	0	aS1-casein	0	Fel d 2	0	Pla a 3	0
V nAna o 2	0	aS2-casein	0	Fel d 4	0	Pla l 1	0
V Ana o 3	0	b-casein	0	Gad c 1	0	Pol d 5	0
Ani s 1	0	K-casein	0	Gal d 1	0	Pru p 1	0
Ani s 3	0	Transferrin	0	Gal d 2	0	Pru p 3	0
Api g 1	0	Bos d 6	0	Gal d 3	0	V Pru du 3	0
Api m 1	0	V BSA	0	Gal d 5	0	V Pru du 4	0
V Api m 1	0.48	Can f 1	0	Gly m 4	0	V Pru du 6	0
V Api m 2	0	Can f 2	0	Gly m 5	0	V Pru du 6.01	0
Api m 4	0	Can f 3	0	Gly m 6	0	V Pru du 6.02	0
V Api m 4	0	V Can f 4	0	Hev b 1	0	Sal k 1	0
Ara h 1	0	Can f 5	0	Hev b 3	0	Ses i 1	0
V Ara h 1	0	V Can f 5	0	Hev b 5	0	Tri a 14	0
Ara h 2	0	V Can f 6	0	Hev b 6.01	0	Tri a 19.0101	0
Ara h 3	0	Che a 1	0	Hev b 8	0	Tri a aA_TI	0
V Ara h 3	0	Cla h 8	0	Jug r 1	0	V Tri a 36 191	0
Ara h 6	0	Cor a 1.0401	0	Jug r 2	1.9	V Tri a 36	0
V Ara h 6	0	Cor a 8	0	Jug r 3	0	V m43	0
Ara h 8	0	Cor a 9	0	Lep d 2	3.72	V m82	0
Ara h 9	0	Cry j 1	5.51	Mal d 1	0.76	Serine	0
Art v 1	0	Cyn d 1	1.93	Mer a 1	0	Thioredoxin	0
Art v 3	0	Cup a 1	6.04	Mus m 1	0	Glutathione	0
Asp f 1	0	Der f 1	0	MUXF3	0.19	peroxiredoxin	0
Asp f 3	0	Der f 2	2.84	Ole e 1	114.27	Profilin	0
Asp f 6	0	Der p 1	0	Ole e 7	0.26	Dehydrin	0
Ber e 1	0	Der p 2	2.67	Ole e 9	0	purothionin	0
Bet v 1	3.21	V Der p 4	0	Par j 2	0	LTP	0
Bet v 2	0	V Der p 5	0	Pen m 1	0	V Ves v 1	0
Bet v 4	0	V Der p 7	0	Pen m 2	0	Ves v 5	0
Bla g 1	0	Der p 10	0	Pen m 4	0	V Ves v 5	0
Bla g 2	0	V Der p 11	0	Phl p 1	21.08	Blank	0

Patient no. PA10							
Act d 1	0	Bla g 5	0	V Der p 14	0	Phl p 2	5.76
Act d 2	0	Bla g 7	0	V Der p 15	0	Phl p 4	0
Act d 5	0	Blo t 5	14.49	V Der p 18	0	Phl p 5	1.04
Act d 8	0	Bos d 4	0	V Der p 21	6.96	Phl p 6	0
Aln g 1	0.4	V Bos d 4	0	V Der p 23	5.2	Phl p 7	0
Alt a 1	0	Bos d 5	0	V clone 16	0	Phl p 11	2.53
Alt a 6	0	V Bos d 5	0	Equ c 1	5.86	Phl p 12	0
Amb a 1	0	Bos d Lactoferrin	0	Equ c 3	0	V Pis v 3	0
V Ana o 1	0	Bos d 8	0	Fag e 2	0	Pla a 1	0
Ana o 2	0	V Bos d 8	0	Fel d 1	8.01	Pla a 2	0
V Ana o 2	0	aS1-casein	0	Fel d 2	0	Pla a 3	0
V nAna o 2	0	aS2-casein	0	Fel d 4	3.79	Pla l 1	15.21
V Ana o 3	0	b-casein	0	Gad c 1	0	Pol d 5	0
Ani s 1	0	K-casein	0	Gal d 1	0	Pru p 1	0
Ani s 3	0	Transferrin	0	Gal d 2	0	Pru p 3	0
Api g 1	0	Bos d 6	0	Gal d 3	0	V Pru du 3	0
Api m 1	0	V BSA	0	Gal d 5	0	V Pru du 4	0
V Api m 1	0	Can f 1	1.45	Gly m 4	0	V Pru du 6	0
V Api m 2	0	Can f 2	1.49	Gly m 5	0	V Pru du 6.01	0
Api m 4	0	Can f 3	0	Gly m 6	0	V Pru du 6.02	0
V Api m 4	0	V Can f 4	4.63	Hev b 1	0	Sal k 1	0
Ara h 1	0	Can f 5	0.44	Hev b 3	0	Ses i 1	0
V Ara h 1	3.89	V Can f 5	0	Hev b 5	0	Tri a 14	0
Ara h 2	0	V Can f 6	1.37	Hev b 6.01	0	Tri a 19.0101	0
Ara h 3	0	Che a 1	0	Hev b 8	0	Tri a aA_TI	0
V Ara h 3	1.62	Cla h 8	0	Jug r 1	0.14	V Tri a 36 191	0
Ara h 6	0.55	Cor a 1.0401	0	Jug r 2	0	V Tri a 36	0
V Ara h 6	1.23	Cor a 8	0	Jug r 3	0	V m43	0
Ara h 8	0	Cor a 9	0	Lep d 2	0	V m82	0
Ara h 9	0	Cry j 1	0.22	Mal d 1	0.45	Serine	0
Art v 1	0	Cyn d 1	19.38	Mer a 1	0	Thioredoxin	0
Art v 3	0	Cup a 1	7.89	Mus m 1	1.24	Glutathione	0
Asp f 1	0	Der f 1	5.01	MUXF3	0	peroxiredoxin	0
Asp f 3	0	Der f 2	44.87	Ole e 1	0	Profilin	0
Asp f 6	0.43	Der p 1	1.04	Ole e 7	0	Dehydrin	0
Ber e 1	0	Der p 2	34.69	Ole e 9	0	purothionin	0
Bet v 1	3.31	V Der p 4	0	Par j 2	0	LTP	0
Bet v 2	0	V Der p 5	0	Pen m 1	0	V Ves v 1	0
Bet v 4	0	V Der p 7	0	Pen m 2	0	Ves v 5	0
Bla g 1	0	Der p 10	0	Pen m 4	0	V Ves v 5	0
Bla g 2	0	V Der p 11	0	Phl p 1	0.93	Blank	0

Patient no. PA11							
Act d 1	0	Bla g 5	0	V Der p 14	0	Phl p 2	0
Act d 2	0	Bla g 7	0	V Der p 15	0	Phl p 4	0.31
Act d 5	0	Blo t 5	0.1	V Der p 18	0	Phl p 5	0
Act d 8	0	Bos d 4	0	V Der p 21	4.22	Phl p 6	0
Aln g 1	0	V Bos d 4	0	V Der p 23	2.63	Phl p 7	0
Alt a 1	0	Bos d 5	0	V clone 16	0	Phl p 11	0
Alt a 6	0	V Bos d 5	0	Equ c 1	0	Phl p 12	0
Amb a 1	0	Bos d Lactoferrin	0	Equ c 3	0	V Pis v 3	0
V Ana o 1	0	Bos d 8	0	Fag e 2	0	Pla a 1	0
Ana o 2	0	V Bos d 8	0	Fel d 1	0	Pla a 2	0
V Ana o 2	0	aS1-casein	0	Fel d 2	0	Pla a 3	0
V nAna o 2	0	aS2-casein	0	Fel d 4	0	Pla l 1	0
V Ana o 3	0	b-casein	0	Gad c 1	0	Pol d 5	0
Ani s 1	0	K-casein	0	Gal d 1	0	Pru p 1	0
Ani s 3	0	Transferrin	0	Gal d 2	0	Pru p 3	0
Api g 1	0	Bos d 6	0	Gal d 3	0	V Pru du 3	0
Api m 1	0	V BSA	0	Gal d 5	0	V Pru du 4	0
V Api m 1	0	Can f 1	31.55	Gly m 4	0	V Pru du 6	0
V Api m 2	0	Can f 2	0.15	Gly m 5	0	V Pru du 6.01	0
Api m 4	0	Can f 3	0	Gly m 6	0	V Pru du 6.02	0
V Api m 4	0	V Can f 4	0	Hev b 1	0	Sal k 1	0
Ara h 1	0	Can f 5	0	Hev b 3	0	Ses i 1	0
V Ara h 1	0	V Can f 5	0	Hev b 5	0	Tri a 14	0
Ara h 2	0	V Can f 6	0	Hev b 6.01	0	Tri a 19.0101	0
Ara h 3	0	Che a 1	0	Hev b 8	0	Tri a aA_TI	0
V Ara h 3	0	Cla h 8	0	Jug r 1	0	V Tri a 36 191	0
Ara h 6	0	Cor a 1.0401	0	Jug r 2	0	V Tri a 36	0
V Ara h 6	0	Cor a 8	0	Jug r 3	0	V m43	0
Ara h 8	0	Cor a 9	0	Lep d 2	0.31	V m82	0
Ara h 9	0	Cry j 1	0	Mal d 1	0	Serine	0
Art v 1	0	Cyn d 1	11.81	Mer a 1	0	Thioredoxin	0
Art v 3	0	Cup a 1	0	Mus m 1	0	Glutathione	0
Asp f 1	0	Der f 1	15.21	MUXF3	0	peroxiredoxin	0
Asp f 3	0	Der f 2	78.88	Ole e 1	14.49	Profilin	0
Asp f 6	0	Der p 1	20.52	Ole e 7	0	Dehydrin	0
Ber e 1	0	Der p 2	78.98	Ole e 9	0	purothionin	0
Bet v 1	0	V Der p 4	0.35	Par j 2	0	LTP	0
Bet v 2	0	V Der p 5	0	Pen m 1	0	V Ves v 1	0
Bet v 4	0	V Der p 7	0	Pen m 2	0	Ves v 5	0
Bla g 1	0	Der p 10	0	Pen m 4	0	V Ves v 5	0
Bla g 2	0	V Der p 11	0	Phl p 1	10.05	Blank	0

Patient no. PA12							
Act d 1	13.72	Bla g 5	0	V Der p 14	0	Phl p 2	0
Act d 2	0	Bla g 7	0	V Der p 15	0	Phl p 4	0
Act d 5	0	Blo t 5	3.06	V Der p 18	0	Phl p 5	5.86
Act d 8	0	Bos d 4	0	V Der p 21	0	Phl p 6	0.4
Aln g 1	0	V Bos d 4	0	V Der p 23	0	Phl p 7	0
Alt a 1	0	Bos d 5	0	V clone 16	0	Phl p 11	0
Alt a 6	0	V Bos d 5	0	Equ c 1	2.16	Phl p 12	0
Amb a 1	0	Bos d Lactoferrin	0.26	Equ c 3	0	V Pis v 3	0
V Ana o 1	0	Bos d 8	0	Fag e 2	0	Pla a 1	0
Ana o 2	0	V Bos d 8	0	Fel d 1	27.74	Pla a 2	0
V Ana o 2	0	aS1-casein	0	Fel d 2	1.98	Pla a 3	0
V nAna o 2	0	aS2-casein	0	Fel d 4	5.15	Pla l 1	0
V Ana o 3	0	b-casein	0	Gad c 1	0	Pol d 5	0
Ani s 1	0.2	K-casein	0	Gal d 1	0	Pru p 1	0
Ani s 3	0	Transferrin	0	Gal d 2	0	Pru p 3	0
Api g 1	1.34	Bos d 6	0.12	Gal d 3	0	V Pru du 3	0.42
Api m 1	0.54	V BSA	0	Gal d 5	0	V Pru du 4	0
V Api m 1	0	Can f 1	38.77	Gly m 4	0	V Pru du 6	0
V Api m 2	0.32	Can f 2	4.08	Gly m 5	0	V Pru du 6.01	0
Api m 4	0	Can f 3	0.98	Gly m 6	0	V Pru du 6.02	0
V Api m 4	0	V Can f 4	0	Hev b 1	0	Sal k 1	0
Ara h 1	0.37	Can f 5	3.68	Hev b 3	0	Ses i 1	0
V Ara h 1	0.18	V Can f 5	0.33	Hev b 5	0	Tri a 14	0
Ara h 2	0	V Can f 6	8.45	Hev b 6.01	0	Tri a 19.0101	0
Ara h 3	0	Che a 1	0	Hev b 8	0	Tri a aA_TI	0
V Ara h 3	0.15	Cla h 8	0	Jug r 1	0	V Tri a 36 191	0
Ara h 6	0	Cor a 1.0401	0	Jug r 2	0	V Tri a 36	0
V Ara h 6	0	Cor a 8	0	Jug r 3	0	V m43	0
Ara h 8	0	Cor a 9	0	Lep d 2	0	V m82	0
Ara h 9	0	Cry j 1	0	Mal d 1	0.13	Serine	0
Art v 1	3.35	Cyn d 1	3.59	Mer a 1	0	Thioredoxin	0
Art v 3	0	Cup a 1	0	Mus m 1	0.95	Glutathione	0
Asp f 1	0	Der f 1	4.3	MUXF3	0	peroxiredoxin	0
Asp f 3	0	Der f 2	0.39	Ole e 1	0	Profilin	0
Asp f 6	0	Der p 1	1.34	Ole e 7	0	Dehydrin	0
Ber e 1	0	Der p 2	0.18	Ole e 9	0	purothionin	0
Bet v 1	0	V Der p 4	0	Par j 2	0	LTP	0
Bet v 2	0	V Der p 5	9.74	Pen m 1	0	V Ves v 1	0
Bet v 4	0	V Der p 7	0.85	Pen m 2	0	Ves v 5	0.11
Bla g 1	0	Der p 10	0	Pen m 4	0	V Ves v 5	0
Bla g 2	0	V Der p 11	0	Phl p 1	10.36	Blank	0

Patient no. PA13							
Act d 1	0	Bla g 5	0	V Der p 14	0.2	Phl p 2	0.14
Act d 2	0.22	Bla g 7	0	V Der p 15	0.24	Phl p 4	1.25
Act d 5	0	Blo t 5	3.17	V Der p 18	6.37	Phl p 5	22.01
Act d 8	0	Bos d 4	0.34	V Der p 21	1.55	Phl p 6	0.8
Aln g 1	0	V Bos d 4	0	V Der p 23	5.8	Phl p 7	0
Alt a 1	1.87	Bos d 5	0	V clone 16	0	Phl p 11	0
Alt a 6	0	V Bos d 5	0	Equ c 1	0	Phl p 12	0
Amb a 1	0	Bos d Lactoferrin	0	Equ c 3	0	V Pis v 3	0
V Ana o 1	0	Bos d 8	0	Fag e 2	0	Pla a 1	0
Ana o 2	0	V Bos d 8	0	Fel d 1	0	Pla a 2	0
V Ana o 2	0	aS1-casein	0	Fel d 2	0	Pla a 3	0
V nAna o 2	0	aS2-casein	0	Fel d 4	0	Pla l 1	0
V Ana o 3	0	b-casein	0	Gad c 1	0	Pol d 5	0.12
Ani s 1	0.21	K-casein	0	Gal d 1	0	Pru p 1	0
Ani s 3	0	Transferrin	0	Gal d 2	0	Pru p 3	0
Api g 1	0.92	Bos d 6	0	Gal d 3	0	V Pru du 3	0
Api m 1	0.38	V BSA	0	Gal d 5	0	V Pru du 4	0
V Api m 1	0.17	Can f 1	0	Gly m 4	0	V Pru du 6	0
V Api m 2	0.28	Can f 2	0	Gly m 5	0	V Pru du 6.01	0
Api m 4	0	Can f 3	0	Gly m 6	0	V Pru du 6.02	0
V Api m 4	0	V Can f 4	0	Hev b 1	0	Sal k 1	0
Ara h 1	0.31	Can f 5	0	Hev b 3	0.37	Ses i 1	0
V Ara h 1	0.19	V Can f 5	0	Hev b 5	0	Tri a 14	0
Ara h 2	0	V Can f 6	0	Hev b 6.01	0.12	Tri a 19.0101	0.24
Ara h 3	0	Che a 1	0	Hev b 8	0.13	Tri a aA_TI	0
V Ara h 3	0.14	Cla h 8	0	Jug r 1	0	V Tri a 36 191	0
Ara h 6	0	Cor a 1.0401	0	Jug r 2	0	V Tri a 36	0
V Ara h 6	0	Cor a 8	0	Jug r 3	0	V m43	0
Ara h 8	0	Cor a 9	0	Lep d 2	0	V m82	0
Ara h 9	0	Cry j 1	0	Mal d 1	0.22	Serine	0
Art v 1	0	Cyn d 1	4.64	Mer a 1	0	Thioredoxin	0
Art v 3	0	Cup a 1	0	Mus m 1	0	Glutathione	0
Asp f 1	0	Der f 1	12.39	MUXF3	0	peroxiredoxin	0
Asp f 3	0	Der f 2	17	Ole e 1	0	Profilin	0
Asp f 6	0	Der p 1	2.87	Ole e 7	0	Dehydrin	0
Ber e 1	0	Der p 2	14.18	Ole e 9	0	purothionin	0
Bet v 1	0	V Der p 4	0	Par j 2	0	LTP	0
Bet v 2	0	V Der p 5	14.24	Pen m 1	0	V Ves v 1	0
Bet v 4	0	V Der p 7	0.96	Pen m 2	0	Ves v 5	0.12
Bla g 1	0	Der p 10	0.32	Pen m 4	0	V Ves v 5	0
Bla g 2	0	V Der p 11	0.21	Phl p 1	12.91	Blank	0

Patient no. PA14

Act d 1	0	Bla g 5	0	V Der p 14	0	Phl p 2	0
Act d 2	0	Bla g 7	0	V Der p 15	0	Phl p 4	0
Act d 5	0	Blo t 5	0.46	V Der p 18	0	Phl p 5	0
Act d 8	0	Bos d 4	0	V Der p 21	4.52	Phl p 6	0
Aln g 1	0	V Bos d 4	0	V Der p 23	7.11	Phl p 7	0
Alt a 1	0	Bos d 5	0	V clone 16	0	Phl p 11	0
Alt a 6	0	V Bos d 5	0	Equ c 1	0	Phl p 12	0
Amb a 1	0	Bos d Lactoferrin	0.25	Equ c 3	0	V Pis v 3	0
V Ana o 1	0	Bos d 8	0	Fag e 2	0	Pla a 1	0
Ana o 2	0	V Bos d 8	0	Fel d 1	0	Pla a 2	0
V Ana o 2	0	aS1-casein	0	Fel d 2	0	Pla a 3	0
V nAna o 2	0	aS2-casein	0	Fel d 4	0	Pla l 1	0
V Ana o 3	0	b-casein	0	Gad c 1	0	Pol d 5	0
Ani s 1	0.12	K-casein	0	Gal d 1	0	Pru p 1	0
Ani s 3	0	Transferrin	0	Gal d 2	0	Pru p 3	0
Api g 1	0.81	Bos d 6	0	Gal d 3	0	V Pru du 3	0
Api m 1	0.31	V BSA	0	Gal d 5	0	V Pru du 4	0
V Api m 1	0.14	Can f 1	0	Gly m 4	0	V Pru du 6	0
V Api m 2	0.19	Can f 2	0	Gly m 5	0	V Pru du 6.01	0
Api m 4	0	Can f 3	0	Gly m 6	0	V Pru du 6.02	0
V Api m 4	0	V Can f 4	0	Hev b 1	0	Sal k 1	0
Ara h 1	0.27	Can f 5	0	Hev b 3	0	Ses i 1	0
V Ara h 1	0.13	V Can f 5	0	Hev b 5	0	Tri a 14	0
Ara h 2	0	V Can f 6	0	Hev b 6.01	0	Tri a 19.0101	0
Ara h 3	0	Che a 1	0	Hev b 8	0	Tri a aA_TI	0
V Ara h 3	0.11	Cla h 8	0	Jug r 1	0	V Tri a 36 191	0
Ara h 6	0	Cor a 1.0401	0	Jug r 2	0	V Tri a 36	0
V Ara h 6	0	Cor a 8	0	Jug r 3	0	V m43	0
Ara h 8	0	Cor a 9	0	Lep d 2	0	V m82	0
Ara h 9	0	Cry j 1	0	Mal d 1	0	Serine	0
Art v 1	0	Cyn d 1	0	Mer a 1	0	Thioredoxin	0
Art v 3	0	Cup a 1	0	Mus m 1	0	Glutathione	0
Asp f 1	0	Der f 1	1.15	MUXF3	0	peroxiredoxin	0
Asp f 3	0	Der f 2	0.11	Ole e 1	0	Profilin	0
Asp f 6	0	Der p 1	0.64	Ole e 7	0	Dehydrin	0
Ber e 1	0	Der p 2	0.13	Ole e 9	0	purothionin	0
Bet v 1	0	V Der p 4	0	Par j 2	0	LTP	0
Bet v 2	0	V Der p 5	9.63	Pen m 1	0	V Ves v 1	0
Bet v 4	0	V Der p 7	0	Pen m 2	0	Ves v 5	0
Bla g 1	0	Der p 10	0	Pen m 4	0	V Ves v 5	0
Bla g 2	0	V Der p 11	0	Phl p 1	0	Blank	0

Patient no. PA15							
Act d 1	2.35	Bla g 5	0	V Der p 14	0	Phl p 2	0.24
Act d 2	0	Bla g 7	0	V Der p 15	0	Phl p 4	0.82
Act d 5	0	Blo t 5	0.3	V Der p 18	0	Phl p 5	0
Act d 8	0	Bos d 4	0	V Der p 21	0.33	Phl p 6	0
Aln g 1	0	V Bos d 4	0	V Der p 23	9.47	Phl p 7	0
Alt a 1	0	Bos d 5	0	V clone 16	0.72	Phl p 11	0
Alt a 6	0	V Bos d 5	0	Equ c 1	0	Phl p 12	0
Amb a 1	0	Bos d Lactoferrin	0.36	Equ c 3	0	V Pis v 3	0
V Ana o 1	0	Bos d 8	0	Fag e 2	0	Pla a 1	0
Ana o 2	0	V Bos d 8	0	Fel d 1	7.47	Pla a 2	0
V Ana o 2	0	aS1-casein	0	Fel d 2	1.52	Pla a 3	0
V nAna o 2	0	aS2-casein	0.18	Fel d 4	0	Pla l 1	0
V Ana o 3	0	b-casein	0.3	Gad c 1	0	Pol d 5	0
Ani s 1	0.32	K-casein	0	Gal d 1	0	Pru p 1	0
Ani s 3	0	Transferrin	0	Gal d 2	0	Pru p 3	0
Api g 1	2.08	Bos d 6	0	Gal d 3	0	V Pru du 3	0
Api m 1	0.63	V BSA	0	Gal d 5	0	V Pru du 4	0
V Api m 1	0	Can f 1	7.91	Gly m 4	0	V Pru du 6	0
V Api m 2	0.41	Can f 2	1.18	Gly m 5	0	V Pru du 6.01	0
Api m 4	0	Can f 3	0	Gly m 6	0	V Pru du 6.02	0
V Api m 4	0	V Can f 4	0	Hev b 1	0.12	Sal k 1	0.15
Ara h 1	0.48	Can f 5	0	Hev b 3	0.2	Ses i 1	0
V Ara h 1	0.33	V Can f 5	0	Hev b 5	0	Tri a 14	0
Ara h 2	0	V Can f 6	0	Hev b 6.01	0	Tri a 19.0101	0
Ara h 3	0.22	Che a 1	0	Hev b 8	0.18	Tri a aA_TI	0
V Ara h 3	0.18	Cla h 8	0	Jug r 1	0	V Tri a 36 191	0
Ara h 6	0	Cor a 1.0401	0.64	Jug r 2	0	V Tri a 36	0.21
V Ara h 6	0	Cor a 8	0	Jug r 3	0	V m43	0
Ara h 8	0	Cor a 9	0	Lep d 2	0	V m82	0
Ara h 9	0	Cry j 1	0	Mal d 1	0.87	Serine	0
Art v 1	0	Cyn d 1	8.63	Mer a 1	0	Thioredoxin	0
Art v 3	0	Cup a 1	1.77	Mus m 1	0	Glutathione	0
Asp f 1	0	Der f 1	2.17	MUXF3	0	peroxiredoxin	0
Asp f 3	0	Der f 2	17.64	Ole e 1	0	Profilin	0
Asp f 6	0	Der p 1	1.2	Ole e 7	0	Dehydrin	0
Ber e 1	0	Der p 2	27.51	Ole e 9	0.14	purothionin	0
Bet v 1	6.99	V Der p 4	0	Par j 2	0	LTP	0
Bet v 2	0	V Der p 5	2.37	Pen m 1	0	V Ves v 1	0
Bet v 4	0	V Der p 7	0.43	Pen m 2	0	Ves v 5	1.37
Bla g 1	0	Der p 10	0	Pen m 4	0	V Ves v 5	0
Bla g 2	0	V Der p 11	0	Phl p 1	1.57	Blank	0

Patient no. PA16							
Act d 1	0	Bla g 5	0	V Der p 14	0	Phl p 2	0
Act d 2	0	Bla g 7	0	V Der p 15	0	Phl p 4	2.33
Act d 5	0	Blo t 5	0	V Der p 18	1.03	Phl p 5	0.55
Act d 8	0	Bos d 4	0	V Der p 21	0.33	Phl p 6	0.15
Aln g 1	3.24	V Bos d 4	0	V Der p 23	0	Phl p 7	0
Alt a 1	0	Bos d 5	0	V clone 16	0.56	Phl p 11	0
Alt a 6	0	V Bos d 5	0	Equ c 1	0	Phl p 12	0.11
Amb a 1	0	Bos d Lactoferrin	0	Equ c 3	0	V Pis v 3	0
V Ana o 1	0	Bos d 8	0	Fag e 2	0	Pla a 1	0
Ana o 2	0	V Bos d 8	0	Fel d 1	0.61	Pla a 2	0
V Ana o 2	0	aS1-casein	0	Fel d 2	0	Pla a 3	0
V nAna o 2	0	aS2-casein	0	Fel d 4	0	Pla l 1	0
V Ana o 3	0	b-casein	0	Gad c 1	0	Pol d 5	0
Ani s 1	0	K-casein	0	Gal d 1	0	Pru p 1	0.43
Ani s 3	0	Transferrin	0	Gal d 2	0	Pru p 3	0
Api g 1	0.14	Bos d 6	0	Gal d 3	0	V Pru du 3	0
Api m 1	0	V BSA	0	Gal d 5	0	V Pru du 4	0.19
V Api m 1	0	Can f 1	0	Gly m 4	0	V Pru du 6	0
V Api m 2	0	Can f 2	0	Gly m 5	0	V Pru du 6.01	0
Api m 4	0	Can f 3	0	Gly m 6	0	V Pru du 6.02	0
V Api m 4	0	V Can f 4	0	Hev b 1	0	Sal k 1	0
Ara h 1	0	Can f 5	0	Hev b 3	0	Ses i 1	0
V Ara h 1	0	V Can f 5	0	Hev b 5	0	Tri a 14	0
Ara h 2	0	V Can f 6	0	Hev b 6.01	0	Tri a 19.0101	0
Ara h 3	0	Che a 1	0	Hev b 8	0.43	Tri a aA_TI	0
V Ara h 3	0	Cla h 8	0	Jug r 1	0	V Tri a 36 191	0
Ara h 6	0	Cor a 1.0401	3.04	Jug r 2	0	V Tri a 36	0
V Ara h 6	0	Cor a 8	0	Jug r 3	0	V m43	0
Ara h 8	0.18	Cor a 9	0	Lep d 2	0	V m82	0
Ara h 9	0	Cry j 1	0	Mal d 1	0.49	Serine	0
Art v 1	0	Cyn d 1	2.09	Mer a 1	0.2	Thioredoxin	0
Art v 3	0	Cup a 1	0	Mus m 1	0	Glutathione	0
Asp f 1	0	Der f 1	8.1	MUXF3	0	peroxiredoxin	0
Asp f 3	0	Der f 2	7.01	Ole e 1	0.23	Profilin	0.13
Asp f 6	0	Der p 1	4.29	Ole e 7	0	Dehydrin	0
Ber e 1	0	Der p 2	2.89	Ole e 9	0	purothionin	0
Bet v 1	5.57	V Der p 4	0	Par j 2	0	LTP	0
Bet v 2	0.32	V Der p 5	0	Pen m 1	0	V Ves v 1	0
Bet v 4	0	V Der p 7	0	Pen m 2	0	Ves v 5	0
Bla g 1	0	Der p 10	0	Pen m 4	0	V Ves v 5	0
Bla g 2	0	V Der p 11	0	Phl p 1	13.43	Blank	0

Patient no. PA17							
Act d 1	0	Bla g 5	0	V Der p 14	0	Phl p 2	0
Act d 2	0	Bla g 7	0	V Der p 15	0	Phl p 4	0
Act d 5	0	Blo t 5	0	V Der p 18	0	Phl p 5	0
Act d 8	0	Bos d 4	0	V Der p 21	0	Phl p 6	0
Aln g 1	0	V Bos d 4	0	V Der p 23	0	Phl p 7	0
Alt a 1	0	Bos d 5	0	V clone 16	0	Phl p 11	0
Alt a 6	0	V Bos d 5	0	Equ c 1	0	Phl p 12	0
Amb a 1	0	Bos d Lactoferrin	0	Equ c 3	0	V Pis v 3	0
V Ana o 1	0	Bos d 8	0	Fag e 2	0	Pla a 1	0
Ana o 2	0	V Bos d 8	0	Fel d 1	0.37	Pla a 2	0
V Ana o 2	0	aS1-casein	0	Fel d 2	0	Pla a 3	0
V nAna o 2	0	aS2-casein	0	Fel d 4	0	Pla l 1	0
V Ana o 3	0	b-casein	0	Gad c 1	0	Pol d 5	0
Ani s 1	0	K-casein	0	Gal d 1	0	Pru p 1	0
Ani s 3	0	Transferrin	0	Gal d 2	0	Pru p 3	0
Api g 1	0	Bos d 6	0	Gal d 3	0	V Pru du 3	0
Api m 1	0	V BSA	0	Gal d 5	0	V Pru du 4	0
V Api m 1	0	Can f 1	0	Gly m 4	0	V Pru du 6	0
V Api m 2	0	Can f 2	0	Gly m 5	0	V Pru du 6.01	0
Api m 4	0	Can f 3	0	Gly m 6	0	V Pru du 6.02	0
V Api m 4	0	V Can f 4	0	Hev b 1	0	Sal k 1	0
Ara h 1	0	Can f 5	0	Hev b 3	0	Ses i 1	0
V Ara h 1	0	V Can f 5	0	Hev b 5	0	Tri a 14	0
Ara h 2	0	V Can f 6	0	Hev b 6.01	0	Tri a 19.0101	0
Ara h 3	0	Che a 1	0	Hev b 8	0	Tri a aA_TI	0
V Ara h 3	0	Cla h 8	0	Jug r 1	0	V Tri a 36 191	0
Ara h 6	0	Cor a 1.0401	0	Jug r 2	0	V Tri a 36	0
V Ara h 6	0	Cor a 8	0	Jug r 3	0	V m43	0
Ara h 8	0	Cor a 9	0	Lep d 2	0	V m82	0
Ara h 9	0	Cry j 1	0	Mal d 1	0	Serine	0
Art v 1	0	Cyn d 1	0	Mer a 1	0	Thioredoxin	0
Art v 3	0	Cup a 1	0	Mus m 1	0	Glutathione	0
Asp f 1	0	Der f 1	0.59	MUXF3	0	peroxiredoxin	0
Asp f 3	0	Der f 2	0.89	Ole e 1	0	Profilin	0
Asp f 6	0	Der p 1	0.31	Ole e 7	0	Dehydrin	0
Ber e 1	0	Der p 2	0.59	Ole e 9	0	purothionin	0
Bet v 1	0	V Der p 4	0	Par j 2	0	LTP	0
Bet v 2	0	V Der p 5	0	Pen m 1	0	V Ves v 1	0
Bet v 4	0	V Der p 7	0	Pen m 2	0.29	Ves v 5	0
Bla g 1	0	Der p 10	0	Pen m 4	0	V Ves v 5	0
Bla g 2	0	V Der p 11	0	Phl p 1	0.5	Blank	0

Patient no. PA18							
Act d 1	0	Bla g 5	0	V Der p 14	0	Phl p 2	0
Act d 2	0	Bla g 7	0	V Der p 15	0	Phl p 4	0
Act d 5	0	Blo t 5	0	V Der p 18	0	Phl p 5	0
Act d 8	0	Bos d 4	0	V Der p 21	0	Phl p 6	0
Aln g 1	4.75	V Bos d 4	0	V Der p 23	4.57	Phl p 7	0
Alt a 1	0	Bos d 5	0	V clone 16	0	Phl p 11	0
Alt a 6	0	V Bos d 5	0	Equ c 1	0	Phl p 12	0
Amb a 1	0	Bos d Lactoferrin	0	Equ c 3	0	V Pis v 3	0
V Ana o 1	0	Bos d 8	0	Fag e 2	0	Pla a 1	0
Ana o 2	0	V Bos d 8	0	Fel d 1	2.65	Pla a 2	0
V Ana o 2	0	aS1-casein	0	Fel d 2	0	Pla a 3	0
V nAna o 2	0	aS2-casein	0	Fel d 4	0	Pla l 1	0
V Ana o 3	0	b-casein	0	Gad c 1	0	Pol d 5	0
Ani s 1	0	K-casein	0	Gal d 1	0	Pru p 1	0.25
Ani s 3	0	Transferrin	0	Gal d 2	0	Pru p 3	0
Api g 1	0	Bos d 6	0	Gal d 3	0	V Pru du 3	0
Api m 1	0	V BSA	0	Gal d 5	0	V Pru du 4	0
V Api m 1	0	Can f 1	8.23	Gly m 4	0	V Pru du 6	0
V Api m 2	0	Can f 2	0	Gly m 5	0	V Pru du 6.01	0
Api m 4	0	Can f 3	0	Gly m 6	0	V Pru du 6.02	0
V Api m 4	0	V Can f 4	0	Hev b 1	0	Sal k 1	0
Ara h 1	0	Can f 5	2.83	Hev b 3	0	Ses i 1	0
V Ara h 1	0	V Can f 5	0	Hev b 5	0	Tri a 14	0
Ara h 2	0	V Can f 6	0	Hev b 6.01	0	Tri a 19.0101	0
Ara h 3	0	Che a 1	0	Hev b 8	0	Tri a aA_TI	0
V Ara h 3	0	Cla h 8	0	Jug r 1	0	V Tri a 36 191	0
Ara h 6	0	Cor a 1.0401	0.42	Jug r 2	0	V Tri a 36	0
V Ara h 6	0	Cor a 8	0	Jug r 3	0	V m43	0
Ara h 8	0	Cor a 9	0	Lep d 2	0	V m82	0
Ara h 9	0	Cry j 1	0	Mal d 1	5.04	Serine	0
Art v 1	0	Cyn d 1	0.25	Mer a 1	0	Thioredoxin	0
Art v 3	0	Cup a 1	0	Mus m 1	0	Glutathione	0
Asp f 1	0	Der f 1	3.51	MUXF3	0	peroxiredoxin	0
Asp f 3	0	Der f 2	0	Ole e 1	0	Profilin	0
Asp f 6	0	Der p 1	0.61	Ole e 7	0	Dehydrin	0
Ber e 1	0	Der p 2	0	Ole e 9	0	purothionin	0
Bet v 1	64.91	V Der p 4	0	Par j 2	0	LTP	0
Bet v 2	0	V Der p 5	0	Pen m 1	0	V Ves v 1	0
Bet v 4	0	V Der p 7	0	Pen m 2	0	Ves v 5	0
Bla g 1	0	Der p 10	0.26	Pen m 4	0	V Ves v 5	0
Bla g 2	0	V Der p 11	0	Phl p 1	0.55	Blank	0

Patient no. PA19							
Act d 1	0	Bla g 5	0	V Der p 14	0	Phl p 2	13.96
Act d 2	0	Bla g 7	0	V Der p 15	0	Phl p 4	0.19
Act d 5	0	Blo t 5	0	V Der p 18	0	Phl p 5	54.98
Act d 8	0.78	Bos d 4	0	V Der p 21	0	Phl p 6	14.14
Aln g 1	4.19	V Bos d 4	0	V Der p 23	24.75	Phl p 7	0.07
Alt a 1	2.62	Bos d 5	0	V clone 16	0	Phl p 11	0
Alt a 6	0	V Bos d 5	0	Equ c 1	0	Phl p 12	1.13
Amb a 1	3.1	Bos d Lactoferrin	0	Equ c 3	0	V Pis v 3	0
V Ana o 1	0	Bos d 8	0	Fag e 2	0	Pla a 1	0
Ana o 2	0	V Bos d 8	0	Fel d 1	0	Pla a 2	0.23
V Ana o 2	0	aS1-casein	0	Fel d 2	0	Pla a 3	0
V nAna o 2	0	aS2-casein	0	Fel d 4	0	Pla l 1	15.15
V Ana o 3	0	b-casein	0	Gad c 1	0	Pol d 5	0
Ani s 1	0	K-casein	0	Gal d 1	0	Pru p 1	11.34
Ani s 3	0	Transferrin	0	Gal d 2	0	Pru p 3	0
Api g 1	2.76	Bos d 6	0	Gal d 3	0	V Pru du 3	0
Api m 1	0	V BSA	0	Gal d 5	0	V Pru du 4	6.2
V Api m 1	0	Can f 1	0	Gly m 4	1.6	V Pru du 6	0
V Api m 2	0	Can f 2	0	Gly m 5	0	V Pru du 6.01	0
Api m 4	0	Can f 3	0	Gly m 6	0	V Pru du 6.02	0
V Api m 4	0	V Can f 4	0	Hev b 1	0	Sal k 1	0
Ara h 1	0	Can f 5	0	Hev b 3	0	Ses i 1	0
V Ara h 1	0	V Can f 5	0	Hev b 5	0	Tri a 14	0
Ara h 2	0	V Can f 6	0	Hev b 6.01	0	Tri a 19.0101	0
Ara h 3	0	Che a 1	0	Hev b 8	9.58	Tri a aA_TI	0
V Ara h 3	0	Cla h 8	0	Jug r 1	0.8	V Tri a 36 191	0
Ara h 6	0	Cor a 1.0401	2.32	Jug r 2	0.27	V Tri a 36	0
V Ara h 6	0	Cor a 8	0	Jug r 3	0	V m43	0
Ara h 8	5.75	Cor a 9	0	Lep d 2	0	V m82	0
Ara h 9	0	Cry j 1	0.26	Mal d 1	9.36	Serine	0
Art v 1	0	Cyn d 1	12.75	Mer a 1	8.34	Thioredoxin	0
Art v 3	0	Cup a 1	0.43	Mus m 1	0	Glutathione	0
Asp f 1	0	Der f 1	0	MUXF3	0	peroxiredoxin	0
Asp f 3	0	Der f 2	56.05	Ole e 1	12.86	Profilin	2.08
Asp f 6	0	Der p 1	0	Ole e 7	0	Dehydrin	0
Ber e 1	0	Der p 2	37.16	Ole e 9	0	purothionin	0
Bet v 1	45.19	V Der p 4	0	Par j 2	0	LTP	0
Bet v 2	4.46	V Der p 5	0	Pen m 1	0	V Ves v 1	0
Bet v 4	0	V Der p 7	0	Pen m 2	0	Ves v 5	0.11
Bla g 1	0	Der p 10	0	Pen m 4	0	V Ves v 5	0
Bla g 2	0	V Der p 11	0	Phl p 1	3.62	Blank	0

Patient no. PA20							
Act d 1	0	Bla g 5	0	V Der p 14	0	Phl p 2	0
Act d 2	0	Bla g 7	0	V Der p 15	0	Phl p 4	0
Act d 5	0	Blo t 5	0	V Der p 18	0	Phl p 5	0
Act d 8	0	Bos d 4	0	V Der p 21	0.52	Phl p 6	0
Aln g 1	0	V Bos d 4	0	V Der p 23	2.07	Phl p 7	0
Alt a 1	0	Bos d 5	0	V clone 16	0	Phl p 11	0
Alt a 6	0	V Bos d 5	0	Equ c 1	0	Phl p 12	0
Amb a 1	0	Bos d Lactoferrin	0	Equ c 3	0	V Pis v 3	0
V Ana o 1	0	Bos d 8	0	Fag e 2	0	Pla a 1	0
Ana o 2	0	V Bos d 8	0	Fel d 1	0	Pla a 2	0
V Ana o 2	0	aS1-casein	0	Fel d 2	0	Pla a 3	0
V nAna o 2	0	aS2-casein	0	Fel d 4	0	Pla l 1	0
V Ana o 3	0	b-casein	0	Gad c 1	0	Pol d 5	0
Ani s 1	0	K-casein	0	Gal d 1	0	Pru p 1	0
Ani s 3	0	Transferrin	0	Gal d 2	0	Pru p 3	0
Api g 1	0	Bos d 6	0	Gal d 3	0	V Pru du 3	0
Api m 1	0	V BSA	0	Gal d 5	0	V Pru du 4	0
V Api m 1	0	Can f 1	0	Gly m 4	0	V Pru du 6	0
V Api m 2	0	Can f 2	0	Gly m 5	0	V Pru du 6.01	0
Api m 4	0	Can f 3	0	Gly m 6	0	V Pru du 6.02	0
V Api m 4	0	V Can f 4	0	Hev b 1	0	Sal k 1	0
Ara h 1	0	Can f 5	0	Hev b 3	0	Ses i 1	0
V Ara h 1	0	V Can f 5	0	Hev b 5	0	Tri a 14	0
Ara h 2	0	V Can f 6	0	Hev b 6.01	0	Tri a 19.0101	0
Ara h 3	0	Che a 1	0	Hev b 8	0	Tri a aA_TI	0
V Ara h 3	0	Cla h 8	0	Jug r 1	0	V Tri a 36 191	0
Ara h 6	0	Cor a 1.0401	0	Jug r 2	0	V Tri a 36	0
V Ara h 6	0	Cor a 8	0	Jug r 3	0	V m43	0
Ara h 8	0	Cor a 9	0	Lep d 2	0	V m82	0
Ara h 9	0	Cry j 1	0	Mal d 1	0	Serine	0
Art v 1	0	Cyn d 1	0	Mer a 1	0	Thioredoxin	0
Art v 3	0	Cup a 1	0	Mus m 1	0	Glutathione	0
Asp f 1	0	Der f 1	1.44	MUXF3	0	peroxiredoxin	0
Asp f 3	0	Der f 2	3.96	Ole e 1	0	Profilin	0
Asp f 6	0	Der p 1	2.02	Ole e 7	0	Dehydrin	0
Ber e 1	0	Der p 2	3.65	Ole e 9	0	purothionin	0
Bet v 1	0	V Der p 4	0	Par j 2	0	LTP	0
Bet v 2	0	V Der p 5	0	Pen m 1	0	V Ves v 1	0
Bet v 4	0	V Der p 7	0	Pen m 2	0	Ves v 5	0
Bla g 1	0	Der p 10	0.33	Pen m 4	0	V Ves v 5	0
Bla g 2	0	V Der p 11	0	Phl p 1	0	Blank	0

Patient no. PA21							
Act d 1	0	Bla g 5	0	V Der p 14	0	Phl p 2	0
Act d 2	0	Bla g 7	0	V Der p 15	0	Phl p 4	0
Act d 5	0	Blo t 5	0	V Der p 18	0	Phl p 5	0
Act d 8	0	Bos d 4	0	V Der p 21	0	Phl p 6	0
Aln g 1	0	V Bos d 4	0	V Der p 23	2.57	Phl p 7	0
Alt a 1	0	Bos d 5	0	V clone 16	0	Phl p 11	0
Alt a 6	0	V Bos d 5	0	Equ c 1	0	Phl p 12	0
Amb a 1	0	Bos d Lactoferrin	0	Equ c 3	0	V Pis v 3	0
V Ana o 1	0	Bos d 8	0	Fag e 2	0	Pla a 1	0
Ana o 2	0	V Bos d 8	0	Fel d 1	0	Pla a 2	0
V Ana o 2	0	aS1-casein	0	Fel d 2	0	Pla a 3	0
V nAna o 2	0	aS2-casein	0	Fel d 4	0	Pla l 1	0
V Ana o 3	0	b-casein	0	Gad c 1	0	Pol d 5	0
Ani s 1	0	K-casein	0	Gal d 1	0	Pru p 1	0
Ani s 3	0	Transferrin	0	Gal d 2	0	Pru p 3	0
Api g 1	0	Bos d 6	0	Gal d 3	0	V Pru du 3	0
Api m 1	0	V BSA	0	Gal d 5	0	V Pru du 4	0
V Api m 1	0	Can f 1	0	Gly m 4	0	V Pru du 6	0
V Api m 2	0	Can f 2	0	Gly m 5	0	V Pru du 6.01	0
Api m 4	0	Can f 3	0	Gly m 6	0	V Pru du 6.02	0
V Api m 4	0	V Can f 4	0	Hev b 1	0	Sal k 1	0
Ara h 1	0	Can f 5	0	Hev b 3	0	Ses i 1	0
V Ara h 1	0	V Can f 5	0	Hev b 5	0	Tri a 14	0
Ara h 2	0	V Can f 6	0	Hev b 6.01	0	Tri a 19.0101	0
Ara h 3	0	Che a 1	0	Hev b 8	0	Tri a aA_TI	0
V Ara h 3	0	Cla h 8	0	Jug r 1	0	V Tri a 36 191	0
Ara h 6	0	Cor a 1.0401	0	Jug r 2	0.14	V Tri a 36	0
V Ara h 6	0	Cor a 8	0	Jug r 3	0	V m43	0
Ara h 8	0	Cor a 9	0	Lep d 2	0	V m82	0
Ara h 9	0	Cry j 1	0	Mal d 1	0	Serine	0
Art v 1	0	Cyn d 1	0.13	Mer a 1	0	Thioredoxin	0
Art v 3	0	Cup a 1	0	Mus m 1	0	Glutathione	0
Asp f 1	0	Der f 1	0.74	MUXF3	0	peroxiredoxin	0
Asp f 3	0	Der f 2	10.84	Ole e 1	0	Profilin	0
Asp f 6	0	Der p 1	0.96	Ole e 7	0	Dehydrin	0
Ber e 1	0	Der p 2	11.11	Ole e 9	0	purothionin	0
Bet v 1	0	V Der p 4	0	Par j 2	0	LTP	0
Bet v 2	0	V Der p 5	0	Pen m 1	0	V Ves v 1	0
Bet v 4	0	V Der p 7	0	Pen m 2	0	Ves v 5	0
Bla g 1	0	Der p 10	0.16	Pen m 4	0	V Ves v 5	0
Bla g 2	0	V Der p 11	0	Phl p 1	0	Blank	0

Patient no. PA22							
Act d 1	0	Bla g 5	0	V Der p 14	0	Phl p 2	6.05
Act d 2	0.11	Bla g 7	0	V Der p 15	0	Phl p 4	0.36
Act d 5	0	Blo t 5	0	V Der p 18	0	Phl p 5	10.6
Act d 8	0	Bos d 4	0	V Der p 21	0	Phl p 6	0.75
Aln g 1	1.29	V Bos d 4	0	V Der p 23	1.05	Phl p 7	0
Alt a 1	0.62	Bos d 5	0	V clone 16	0	Phl p 11	0
Alt a 6	0	V Bos d 5	0	Equ c 1	0	Phl p 12	0
Amb a 1	0.48	Bos d Lactoferrin	0	Equ c 3	0	V Pis v 3	0
V Ana o 1	0	Bos d 8	0	Fag e 2	0	Pla a 1	0
Ana o 2	0.79	V Bos d 8	0	Fel d 1	0.52	Pla a 2	0
V Ana o 2	0	aS1-casein	0	Fel d 2	0	Pla a 3	0
V nAna o 2	0	aS2-casein	0	Fel d 4	0	Pla l 1	0
V Ana o 3	0	b-casein	0	Gad c 1	0	Pol d 5	0
Ani s 1	0	K-casein	0	Gal d 1	0	Pru p 1	0.09
Ani s 3	0	Transferrin	0	Gal d 2	0	Pru p 3	0
Api g 1	0	Bos d 6	0	Gal d 3	0	V Pru du 3	0
Api m 1	0	V BSA	0	Gal d 5	0	V Pru du 4	0.45
V Api m 1	0.34	Can f 1	0	Gly m 4	0.09	V Pru du 6	0
V Api m 2	0	Can f 2	0	Gly m 5	0	V Pru du 6.01	0
Api m 4	0	Can f 3	0	Gly m 6	0	V Pru du 6.02	0
V Api m 4	0	V Can f 4	0	Hev b 1	0	Sal k 1	0
Ara h 1	0	Can f 5	0	Hev b 3	0	Ses i 1	0
V Ara h 1	0	V Can f 5	0	Hev b 5	0	Tri a 14	0
Ara h 2	0	V Can f 6	0	Hev b 6.01	0	Tri a 19.0101	0
Ara h 3	0	Che a 1	0	Hev b 8	0.63	Tri a aA_TI	0
V Ara h 3	0	Cla h 8	0	Jug r 1	0	V Tri a 36 191	0
Ara h 6	0	Cor a 1.0401	2.71	Jug r 2	0.17	V Tri a 36	0
V Ara h 6	0	Cor a 8	0	Jug r 3	0	V m43	0
Ara h 8	0.07	Cor a 9	0	Lep d 2	0	V m82	0
Ara h 9	0	Cry j 1	0	Mal d 1	1.95	Serine	0
Art v 1	0.57	Cyn d 1	2.33	Mer a 1	0.69	Thioredoxin	0
Art v 3	0	Cup a 1	0	Mus m 1	0	Glutathione	0
Asp f 1	0	Der f 1	0	MUXF3	0	peroxiredoxin	0
Asp f 3	0	Der f 2	0	Ole e 1	0.26	Profilin	0
Asp f 6	0	Der p 1	0	Ole e 7	0	Dehydrin	0
Ber e 1	0	Der p 2	0	Ole e 9	0	purothionin	0
Bet v 1	17.86	V Der p 4	0	Par j 2	0	LTP	0
Bet v 2	0.21	V Der p 5	0	Pen m 1	0	V Ves v 1	0
Bet v 4	0	V Der p 7	0	Pen m 2	0	Ves v 5	0
Bla g 1	0	Der p 10	0	Pen m 4	0	V Ves v 5	0
Bla g 2	0	V Der p 11	0	Phl p 1	0	Blank	0

Patient no. PA23							
Act d 1	0	Bla g 5	0	V Der p 14	0	Phl p 2	0
Act d 2	0	Bla g 7	0	V Der p 15	0.2	Phl p 4	0
Act d 5	0	Blo t 5	0.42	V Der p 18	1.07	Phl p 5	0
Act d 8	0	Bos d 4	0	V Der p 21	8.7	Phl p 6	0
Aln g 1	0	V Bos d 4	0	V Der p 23	17.93	Phl p 7	0
Alt a 1	0	Bos d 5	0	V clone 16	1.78	Phl p 11	0
Alt a 6	0	V Bos d 5	0	Equ c 1	0	Phl p 12	0
Amb a 1	0	Bos d Lactoferrin	0.42	Equ c 3	0	V Pis v 3	0
V Ana o 1	0	Bos d 8	0	Fag e 2	0	Pla a 1	0
Ana o 2	0	V Bos d 8	0	Fel d 1	0	Pla a 2	0
V Ana o 2	0	aS1-casein	0	Fel d 2	0.09	Pla a 3	0
V nAna o 2	0	aS2-casein	0.39	Fel d 4	0	Pla l 1	0
V Ana o 3	0	b-casein	0	Gad c 1	0	Pol d 5	0.74
Ani s 1	0.19	K-casein	0	Gal d 1	0	Pru p 1	0
Ani s 3	0	Transferrin	0.24	Gal d 2	0	Pru p 3	0
Api g 1	0.91	Bos d 6	0.36	Gal d 3	0	V Pru du 3	0
Api m 1	0.45	V BSA	0.34	Gal d 5	0	V Pru du 4	0
V Api m 1	0.13	Can f 1	0	Gly m 4	0	V Pru du 6	0
V Api m 2	0.28	Can f 2	0	Gly m 5	0	V Pru du 6.01	0
Api m 4	0.17	Can f 3	0.1	Gly m 6	0	V Pru du 6.02	0
V Api m 4	0	V Can f 4	0	Hev b 1	0	Sal k 1	0.12
Ara h 1	0.37	Can f 5	0	Hev b 3	0.36	Ses i 1	0
V Ara h 1	0.23	V Can f 5	0	Hev b 5	0.3	Tri a 14	0
Ara h 2	0	V Can f 6	0	Hev b 6.01	0.11	Tri a 19.0101	0.15
Ara h 3	0	Che a 1	0	Hev b 8	0.21	Tri a aA_TI	0
V Ara h 3	0	Cla h 8	0	Jug r 1	0	V Tri a 36 191	0
Ara h 6	0	Cor a 1.0401	0	Jug r 2	0	V Tri a 36	0
V Ara h 6	0	Cor a 8	0	Jug r 3	0	V m43	0
Ara h 8	0	Cor a 9	0	Lep d 2	0	V m82	0
Ara h 9	0	Cry j 1	0.13	Mal d 1	0	Serine	0
Art v 1	0	Cyn d 1	0.15	Mer a 1	0	Thioredoxin	0
Art v 3	0	Cup a 1	0	Mus m 1	0	Glutathione	0
Asp f 1	0.15	Der f 1	2.66	MUXF3	0	peroxiredoxin	0
Asp f 3	0	Der f 2	16.85	Ole e 1	0	Profilin	0
Asp f 6	0	Der p 1	1.76	Ole e 7	0	Dehydrin	0
Ber e 1	0	Der p 2	43.63	Ole e 9	0.3	purothionin	0
Bet v 1	0	V Der p 4	0	Par j 2	0	LTP	0
Bet v 2	0	V Der p 5	29.95	Pen m 1	0	V Ves v 1	0
Bet v 4	0	V Der p 7	39.06	Pen m 2	0	Ves v 5	0.11
Bla g 1	0	Der p 10	0	Pen m 4	0	V Ves v 5	0
Bla g 2	0	V Der p 11	0	Phl p 1	0	Blank	0

Patient no. PA24							
Act d 1	0	Bla g 5	0	V Der p 14	0	Phl p 2	0
Act d 2	0.08	Bla g 7	0	V Der p 15	0	Phl p 4	0.33
Act d 5	0	Blo t 5	0	V Der p 18	0	Phl p 5	2.69
Act d 8	0.15	Bos d 4	0	V Der p 21	2.62	Phl p 6	0.43
Aln g 1	0.6	V Bos d 4	0	V Der p 23	3.51	Phl p 7	0
Alt a 1	0.27	Bos d 5	0	V clone 16	0	Phl p 11	0
Alt a 6	0.42	V Bos d 5	0	Equ c 1	0	Phl p 12	0
Amb a 1	0.5	Bos d Lactoferrin	0.34	Equ c 3	0	V Pis v 3	0
V Ana o 1	0	Bos d 8	0	Fag e 2	0	Pla a 1	0
Ana o 2	0	V Bos d 8	0	Fel d 1	0.68	Pla a 2	0
V Ana o 2	0	aS1-casein	0	Fel d 2	0.31	Pla a 3	0
V nAna o 2	0	aS2-casein	0	Fel d 4	0	Pla l 1	0
V Ana o 3	0	b-casein	0	Gad c 1	0	Pol d 5	0
Ani s 1	0	K-casein	0	Gal d 1	0	Pru p 1	0.28
Ani s 3	0	Transferrin	0.45	Gal d 2	0	Pru p 3	0
Api g 1	0	Bos d 6	0	Gal d 3	0	V Pru du 3	0
Api m 1	0	V BSA	0	Gal d 5	0	V Pru du 4	0
V Api m 1	0.07	Can f 1	0.17	Gly m 4	0.2	V Pru du 6	0
V Api m 2	0	Can f 2	0	Gly m 5	0	V Pru du 6.01	0
Api m 4	0	Can f 3	0	Gly m 6	0	V Pru du 6.02	0
V Api m 4	0	V Can f 4	0	Hev b 1	0	Sal k 1	0
Ara h 1	0	Can f 5	0.17	Hev b 3	0	Ses i 1	0
V Ara h 1	0	V Can f 5	0	Hev b 5	0	Tri a 14	0
Ara h 2	0	V Can f 6	0	Hev b 6.01	0	Tri a 19.0101	0
Ara h 3	0	Che a 1	0	Hev b 8	0	Tri a aA_TI	0
V Ara h 3	0	Cla h 8	0	Jug r 1	0	V Tri a 36 191	0
Ara h 6	0	Cor a 1.0401	1.08	Jug r 2	0.32	V Tri a 36	0
V Ara h 6	0	Cor a 8	0	Jug r 3	0	V m43	0
Ara h 8	0.55	Cor a 9	0	Lep d 2	0	V m82	0
Ara h 9	0	Cry j 1	0	Mal d 1	2.48	Serine	0
Art v 1	3.06	Cyn d 1	0.72	Mer a 1	0	Thioredoxin	0
Art v 3	0	Cup a 1	0	Mus m 1	0	Glutathione	0
Asp f 1	0	Der f 1	0.11	MUXF3	0	peroxiredoxin	0
Asp f 3	0	Der f 2	6.5	Ole e 1	7.69	Profilin	0
Asp f 6	0	Der p 1	0.41	Ole e 7	0	Dehydrin	0
Ber e 1	0	Der p 2	5.34	Ole e 9	0	purothionin	0
Bet v 1	8.94	V Der p 4	0	Par j 2	0	LTP	0
Bet v 2	0	V Der p 5	3.84	Pen m 1	0	V Ves v 1	0
Bet v 4	0	V Der p 7	0.6	Pen m 2	0	Ves v 5	0.42
Bla g 1	0	Der p 10	0	Pen m 4	0	V Ves v 5	0
Bla g 2	0	V Der p 11	0	Phl p 1	0.75	Blank	0

Patient no. PA25							
Act d 1	0	Bla g 5	0	V Der p 14	0	Phl p 2	0
Act d 2	0	Bla g 7	0	V Der p 15	0	Phl p 4	0
Act d 5	0	Blo t 5	0	V Der p 18	0	Phl p 5	0
Act d 8	0	Bos d 4	0	V Der p 21	0.31	Phl p 6	0
Aln g 1	0	V Bos d 4	0	V Der p 23	0.32	Phl p 7	0
Alt a 1	0	Bos d 5	0	V clone 16	0.11	Phl p 11	0
Alt a 6	0	V Bos d 5	0	Equ c 1	0	Phl p 12	0
Amb a 1	0	Bos d Lactoferrin	0	Equ c 3	0	V Pis v 3	0
V Ana o 1	0	Bos d 8	0	Fag e 2	0	Pla a 1	0
Ana o 2	0	V Bos d 8	0	Fel d 1	0	Pla a 2	0
V Ana o 2	0	aS1-casein	0	Fel d 2	0	Pla a 3	0
V nAna o 2	0	aS2-casein	0	Fel d 4	0	Pla l 1	0
V Ana o 3	0	b-casein	0	Gad c 1	0	Pol d 5	0
Ani s 1	0	K-casein	0	Gal d 1	0	Pru p 1	0
Ani s 3	0	Transferrin	0	Gal d 2	0	Pru p 3	0
Api g 1	0	Bos d 6	0	Gal d 3	0	V Pru du 3	0
Api m 1	0	V BSA	0	Gal d 5	0	V Pru du 4	0
V Api m 1	0	Can f 1	0	Gly m 4	0	V Pru du 6	0
V Api m 2	0	Can f 2	0	Gly m 5	0	V Pru du 6.01	0
Api m 4	0	Can f 3	0	Gly m 6	0	V Pru du 6.02	0
V Api m 4	0	V Can f 4	0	Hev b 1	0	Sal k 1	0
Ara h 1	0	Can f 5	0	Hev b 3	0	Ses i 1	0
V Ara h 1	0	V Can f 5	0	Hev b 5	0	Tri a 14	0
Ara h 2	0	V Can f 6	0	Hev b 6.01	0	Tri a 19.0101	0
Ara h 3	0	Che a 1	0	Hev b 8	0	Tri a aA_TI	0
V Ara h 3	0	Cla h 8	0	Jug r 1	0	V Tri a 36 191	0
Ara h 6	0	Cor a 1.0401	0	Jug r 2	0	V Tri a 36	0
V Ara h 6	0	Cor a 8	0	Jug r 3	0	V m43	0
Ara h 8	0	Cor a 9	0	Lep d 2	0	V m82	0
Ara h 9	0	Cry j 1	0	Mal d 1	0	Serine	0
Art v 1	0	Cyn d 1	0	Mer a 1	0	Thioredoxin	0
Art v 3	0	Cup a 1	0	Mus m 1	0	Glutathione	0
Asp f 1	0	Der f 1	0	MUXF3	0	peroxiredoxin	0
Asp f 3	0	Der f 2	0.42	Ole e 1	0	Profilin	0
Asp f 6	0	Der p 1	0.25	Ole e 7	0	Dehydrin	0
Ber e 1	0	Der p 2	0.41	Ole e 9	0	purothionin	0
Bet v 1	0	V Der p 4	0	Par j 2	0	LTP	0
Bet v 2	0	V Der p 5	0.46	Pen m 1	0	V Ves v 1	0
Bet v 4	0	V Der p 7	0.52	Pen m 2	0	Ves v 5	0
Bla g 1	0	Der p 10	0	Pen m 4	0	V Ves v 5	0
Bla g 2	0	V Der p 11	0	Phl p 1	0	Blank	0

Patient no. PA26							
Act d 1	0	Bla g 5	0	V Der p 14	0	Phl p 2	0
Act d 2	0	Bla g 7	0	V Der p 15	0	Phl p 4	0.56
Act d 5	0	Blo t 5	0.81	V Der p 18	0	Phl p 5	5.97
Act d 8	0	Bos d 4	0	V Der p 21	0.67	Phl p 6	0
Aln g 1	0	V Bos d 4	0	V Der p 23	0.21	Phl p 7	0
Alt a 1	0	Bos d 5	0	V clone 16	0	Phl p 11	0
Alt a 6	0	V Bos d 5	0	Equ c 1	0	Phl p 12	0
Amb a 1	0	Bos d Lactoferrin	0	Equ c 3	0	V Pis v 3	0
V Ana o 1	0	Bos d 8	0	Fag e 2	0	Pla a 1	0
Ana o 2	0	V Bos d 8	0	Fel d 1	1.33	Pla a 2	0
V Ana o 2	0	aS1-casein	0	Fel d 2	0	Pla a 3	0
V nAna o 2	0	aS2-casein	0	Fel d 4	0	Pla l 1	0
V Ana o 3	0	b-casein	0	Gad c 1	0	Pol d 5	0
Ani s 1	0	K-casein	0	Gal d 1	0	Pru p 1	0
Ani s 3	0	Transferrin	0	Gal d 2	0	Pru p 3	0
Api g 1	0	Bos d 6	0	Gal d 3	0	V Pru du 3	0
Api m 1	0	V BSA	0	Gal d 5	0	V Pru du 4	0
V Api m 1	0	Can f 1	0	Gly m 4	0	V Pru du 6	0
V Api m 2	0	Can f 2	0	Gly m 5	0	V Pru du 6.01	0
Api m 4	0	Can f 3	0	Gly m 6	0	V Pru du 6.02	0
V Api m 4	0	V Can f 4	0	Hev b 1	0	Sal k 1	0
Ara h 1	0	Can f 5	0	Hev b 3	0	Ses i 1	0
V Ara h 1	0	V Can f 5	0	Hev b 5	0	Tri a 14	0
Ara h 2	0	V Can f 6	0	Hev b 6.01	0	Tri a 19.0101	0
Ara h 3	0	Che a 1	0	Hev b 8	0	Tri a aA_TI	0
V Ara h 3	0	Cla h 8	0	Jug r 1	0	V Tri a 36 191	0
Ara h 6	0	Cor a 1.0401	0	Jug r 2	0	V Tri a 36	0
V Ara h 6	0	Cor a 8	0	Jug r 3	0	V m43	0
Ara h 8	0	Cor a 9	0	Lep d 2	0	V m82	0
Ara h 9	0	Cry j 1	0	Mal d 1	0	Serine	0
Art v 1	0	Cyn d 1	0	Mer a 1	0	Thioredoxin	0
Art v 3	0	Cup a 1	0	Mus m 1	0	Glutathione	0
Asp f 1	0	Der f 1	0.33	MUXF3	0	peroxiredoxin	0
Asp f 3	0	Der f 2	1.84	Ole e 1	0	Profilin	0
Asp f 6	0	Der p 1	0.57	Ole e 7	0	Dehydrin	0
Ber e 1	0	Der p 2	2.1	Ole e 9	0	purothionin	0
Bet v 1	0	V Der p 4	0	Par j 2	0	LTP	0
Bet v 2	0	V Der p 5	2.14	Pen m 1	0	V Ves v 1	0
Bet v 4	0	V Der p 7	0.16	Pen m 2	0	Ves v 5	0
Bla g 1	0	Der p 10	0	Pen m 4	0	V Ves v 5	0
Bla g 2	0	V Der p 11	0	Phl p 1	0	Blank	0

Patient no. PA27							
Act d 1	0	Bla g 5	0	V Der p 14	0	Phl p 2	0
Act d 2	0	Bla g 7	0	V Der p 15	0	Phl p 4	0
Act d 5	0	Blo t 5	0	V Der p 18	0	Phl p 5	0
Act d 8	0	Bos d 4	0	V Der p 21	0	Phl p 6	0
Aln g 1	0	V Bos d 4	0	V Der p 23	0.81	Phl p 7	0
Alt a 1	0	Bos d 5	0	V clone 16	0	Phl p 11	0
Alt a 6	0	V Bos d 5	0	Equ c 1	0	Phl p 12	0
Amb a 1	0	Bos d Lactoferrin	0	Equ c 3	0	V Pis v 3	0
V Ana o 1	0	Bos d 8	0	Fag e 2	0	Pla a 1	0
Ana o 2	0	V Bos d 8	0	Fel d 1	0	Pla a 2	0
V Ana o 2	0	aS1-casein	0	Fel d 2	0	Pla a 3	0
V nAna o 2	0	aS2-casein	0	Fel d 4	0	Pla l 1	0
V Ana o 3	0	b-casein	0	Gad c 1	0	Pol d 5	0
Ani s 1	0	K-casein	0	Gal d 1	0	Pru p 1	0
Ani s 3	0	Transferrin	0	Gal d 2	0	Pru p 3	0
Api g 1	0	Bos d 6	0	Gal d 3	0	V Pru du 3	0
Api m 1	0	V BSA	0	Gal d 5	0	V Pru du 4	0
V Api m 1	0	Can f 1	0	Gly m 4	0	V Pru du 6	0
V Api m 2	0	Can f 2	0	Gly m 5	0	V Pru du 6.01	0
Api m 4	0	Can f 3	0	Gly m 6	0	V Pru du 6.02	0
V Api m 4	0	V Can f 4	0	Hev b 1	0	Sal k 1	0
Ara h 1	0	Can f 5	0	Hev b 3	0	Ses i 1	0
V Ara h 1	0	V Can f 5	0	Hev b 5	0	Tri a 14	0
Ara h 2	0	V Can f 6	0	Hev b 6.01	0	Tri a 19.0101	0
Ara h 3	0	Che a 1	0	Hev b 8	0	Tri a aA_TI	0
V Ara h 3	0	Cla h 8	0	Jug r 1	0	V Tri a 36 191	0
Ara h 6	0	Cor a 1.0401	0.2	Jug r 2	0	V Tri a 36	0
V Ara h 6	0	Cor a 8	0	Jug r 3	0	V m43	0
Ara h 8	0	Cor a 9	0	Lep d 2	0	V m82	0
Ara h 9	0.12	Cry j 1	0	Mal d 1	0.66	Serine	0
Art v 1	0	Cyn d 1	0	Mer a 1	0	Thioredoxin	0
Art v 3	0	Cup a 1	0	Mus m 1	0	Glutathione	0
Asp f 1	0	Der f 1	0.09	MUXF3	0	peroxiredoxin	0
Asp f 3	0	Der f 2	0	Ole e 1	2.18	Profilin	0
Asp f 6	0	Der p 1	0.31	Ole e 7	0	Dehydrin	0
Ber e 1	0	Der p 2	0	Ole e 9	0	purothionin	0
Bet v 1	6.54	V Der p 4	0	Par j 2	0	LTP	0
Bet v 2	0	V Der p 5	0	Pen m 1	0	V Ves v 1	0
Bet v 4	0	V Der p 7	0	Pen m 2	0	Ves v 5	0
Bla g 1	0	Der p 10	0	Pen m 4	0	V Ves v 5	0
Bla g 2	0	V Der p 11	0	Phl p 1	0	Blank	0

Donor no. NDP1							
Act d 1	0	Bla g 5	0	V Der p 14	0	Phl p 2	0
Act d 2	0	Bla g 7	0	V Der p 15	0	Phl p 4	0
Act d 5	0	Blo t 5	0	V Der p 18	0	Phl p 5	0
Act d 8	0	Bos d 4	0	V Der p 21	0	Phl p 6	0
Aln g 1	0	V Bos d 4	0	V Der p 23	0	Phl p 7	0
Alt a 1	0	Bos d 5	0	V clone 16	0	Phl p 11	0
Alt a 6	0.25	V Bos d 5	0	Equ c 1	0	Phl p 12	0
Amb a 1	0	Bos d Lactoferrin	0	Equ c 3	0	V Pis v 3	0
V Ana o 1	0	Bos d 8	0	Fag e 2	0	Pla a 1	0.56
Ana o 2	1.17	V Bos d 8	0	Fel d 1	0	Pla a 2	0
V Ana o 2	0	aS1-casein	0	Fel d 2	0	Pla a 3	0
V nAna o 2	0	aS2-casein	0	Fel d 4	0	Pla l 1	0
V Ana o 3	0	b-casein	0	Gad c 1	0	Pol d 5	0
Ani s 1	0	K-casein	0	Gal d 1	0	Pru p 1	0
Ani s 3	0	Transferrin	0	Gal d 2	0	Pru p 3	0
Api g 1	0	Bos d 6	0	Gal d 3	0	V Pru du 3	0
Api m 1	0	V BSA	0	Gal d 5	0.77	V Pru du 4	0
V Api m 1	0.48	Can f 1	0	Gly m 4	0	V Pru du 6	0
V Api m 2	0	Can f 2	0	Gly m 5	8.09	V Pru du 6.01	0
Api m 4	0	Can f 3	0	Gly m 6	0	V Pru du 6.02	0
V Api m 4	0	V Can f 4	0	Hev b 1	2.09	Sal k 1	0
Ara h 1	0.38	Can f 5	0	Hev b 3	1.08	Ses i 1	0
V Ara h 1	0	V Can f 5	0	Hev b 5	0	Tri a 14	0
Ara h 2	0	V Can f 6	0	Hev b 6.01	0.17	Tri a 19.0101	0.86
Ara h 3	0	Che a 1	0	Hev b 8	0	Tri a aA_TI	0
V Ara h 3	0	Cla h 8	0	Jug r 1	0	V Tri a 36 191	0
Ara h 6	0	Cor a 1.0401	0	Jug r 2	0	V Tri a 36	0
V Ara h 6	0	Cor a 8	0	Jug r 3	0	V m43	0
Ara h 8	0	Cor a 9	0	Lep d 2	0	V m82	0
Ara h 9	0	Cry j 1	0	Mal d 1	0	Serine	0
Art v 1	0	Cyn d 1	0	Mer a 1	0	Thioredoxin	0
Art v 3	0	Cup a 1	0	Mus m 1	0	Glutathione	0
Asp f 1	0	Der f 1	0	MUXF3	0	peroxiredoxin	0
Asp f 3	0	Der f 2	0	Ole e 1	0	Profilin	0
Asp f 6	0	Der p 1	0	Ole e 7	0	Dehydrin	0
Ber e 1	0	Der p 2	0	Ole e 9	0	purothionin	0
Bet v 1	0	V Der p 4	0	Par j 2	0	LTP	0
Bet v 2	0	V Der p 5	0	Pen m 1	0	V Ves v 1	0
Bet v 4	0	V Der p 7	0	Pen m 2	0	Ves v 5	0.14
Bla g 1	0	Der p 10	0	Pen m 4	0.17	V Ves v 5	0
Bla g 2	0	V Der p 11	0	Phl p 1	0.81	Blank	0

Donor no. NDP2							
Act d 1	0	Bla g 5	0	V Der p 14	0	Phl p 2	20.39
Act d 2	1.55	Bla g 7	0	V Der p 15	0	Phl p 4	0.33
Act d 5	0	Blo t 5	0	V Der p 18	0	Phl p 5	12.91
Act d 8	0	Bos d 4	0	V Der p 21	0	Phl p 6	9.73
Aln g 1	16.73	V Bos d 4	0	V Der p 23	0	Phl p 7	0
Alt a 1	0	Bos d 5	0	V clone 16	0	Phl p 11	0
Alt a 6	0	V Bos d 5	0	Equ c 1	62.03	Phl p 12	0
Amb a 1	0	Bos d Lactoferrin	0	Equ c 3	0.38	V Pis v 3	0
V Ana o 1	0	Bos d 8	0	Fag e 2	0	Pla a 1	0
Ana o 2	0	V Bos d 8	0	Fel d 1	36.75	Pla a 2	0
V Ana o 2	0	aS1-casein	0	Fel d 2	0.33	Pla a 3	0
V nAna o 2	0	aS2-casein	0	Fel d 4	27.24	Pla l 1	0
V Ana o 3	0	b-casein	0	Gad c 1	0	Pol d 5	0
Ani s 1	0	K-casein	0	Gal d 1	0	Pru p 1	1.29
Ani s 3	0	Transferrin	0	Gal d 2	0	Pru p 3	0
Api g 1	3.27	Bos d 6	0	Gal d 3	0	V Pru du 3	0
Api m 1	0	V BSA	0	Gal d 5	0.18	V Pru du 4	0
V Api m 1	0	Can f 1	63.36	Gly m 4	0.24	V Pru du 6	0
V Api m 2	0	Can f 2	66.45	Gly m 5	0	V Pru du 6.01	0
Api m 4	0	Can f 3	0.47	Gly m 6	0	V Pru du 6.02	0
V Api m 4	0	V Can f 4	45.75	Hev b 1	0	Sal k 1	0
Ara h 1	0	Can f 5	2.78	Hev b 3	0	Ses i 1	0
V Ara h 1	0	V Can f 5	0.67	Hev b 5	0	Tri a 14	0
Ara h 2	0	V Can f 6	32.3	Hev b 6.01	0	Tri a 19.0101	0
Ara h 3	0	Che a 1	0.27	Hev b 8	0.21	Tri a aA_TI	0
V Ara h 3	0	Cla h 8	0	Jug r 1	0	V Tri a 36 191	0
Ara h 6	0	Cor a 1.0401	9.18	Jug r 2	0.16	V Tri a 36	0
V Ara h 6	0	Cor a 8	0	Jug r 3	0	V m43	0
Ara h 8	7.78	Cor a 9	0	Lep d 2	0	V m82	0
Ara h 9	0	Cry j 1	0.5	Mal d 1	7.25	Serine	0
Art v 1	0	Cyn d 1	9.64	Mer a 1	0.21	Thioredoxin	0
Art v 3	0	Cup a 1	1.31	Mus m 1	11.67	Glutathione	0
Asp f 1	0	Der f 1	0	MUXF3	0	peroxiredoxin	0
Asp f 3	0	Der f 2	0	Ole e 1	0.32	Profilin	0
Asp f 6	0	Der p 1	0	Ole e 7	0	Dehydrin	0
Ber e 1	0	Der p 2	0	Ole e 9	0	purothionin	0
Bet v 1	45.02	V Der p 4	0	Par j 2	0	LTP	0
Bet v 2	0.24	V Der p 5	0	Pen m 1	0	V Ves v 1	0
Bet v 4	0	V Der p 7	0	Pen m 2	0	Ves v 5	0
Bla g 1	0	Der p 10	0	Pen m 4	0	V Ves v 5	0
Bla g 2	0.17	V Der p 11	0	Phl p 1	25.86	Blank	0

Donor no. NDP3							
Act d 1	0	Bla g 5	0	V Der p 14	0	Phl p 2	0
Act d 2	0	Bla g 7	0	V Der p 15	0	Phl p 4	0
Act d 5	0	Blo t 5	0	V Der p 18	0	Phl p 5	0
Act d 8	0	Bos d 4	0	V Der p 21	0	Phl p 6	0
Aln g 1	0	V Bos d 4	0	V Der p 23	0	Phl p 7	0
Alt a 1	0	Bos d 5	0	V clone 16	0	Phl p 11	0
Alt a 6	0	V Bos d 5	0	Equ c 1	0	Phl p 12	0
Amb a 1	0	Bos d Lactoferrin	0	Equ c 3	0	V Pis v 3	0
V Ana o 1	0	Bos d 8	0	Fag e 2	0	Pla a 1	0
Ana o 2	0	V Bos d 8	0	Fel d 1	0.3	Pla a 2	0
V Ana o 2	0	aS1-casein	0	Fel d 2	0	Pla a 3	0
V nAna o 2	0	aS2-casein	0	Fel d 4	0.15	Pla l 1	0
V Ana o 3	0	b-casein	0	Gad c 1	0	Pol d 5	0
Ani s 1	0	K-casein	0	Gal d 1	0	Pru p 1	0
Ani s 3	0	Transferrin	0.13	Gal d 2	0	Pru p 3	0
Api g 1	0	Bos d 6	0	Gal d 3	0	V Pru du 3	0
Api m 1	0	V BSA	0	Gal d 5	0	V Pru du 4	0
V Api m 1	0	Can f 1	0.3	Gly m 4	0	V Pru du 6	0
V Api m 2	0	Can f 2	0	Gly m 5	0	V Pru du 6.01	0
Api m 4	0	Can f 3	0	Gly m 6	0	V Pru du 6.02	0
V Api m 4	0	V Can f 4	0	Hev b 1	0	Sal k 1	0
Ara h 1	0	Can f 5	0	Hev b 3	0	Ses i 1	0
V Ara h 1	0	V Can f 5	0	Hev b 5	0	Tri a 14	0
Ara h 2	0	V Can f 6	0	Hev b 6.01	0	Tri a 19.0101	0
Ara h 3	0	Che a 1	0	Hev b 8	0	Tri a aA_TI	0
V Ara h 3	0	Cla h 8	0	Jug r 1	0	V Tri a 36 191	0
Ara h 6	0	Cor a 1.0401	0	Jug r 2	0	V Tri a 36	0
V Ara h 6	0	Cor a 8	0	Jug r 3	0	V m43	0
Ara h 8	0	Cor a 9	0	Lep d 2	0	V m82	0
Ara h 9	0	Cry j 1	0	Mal d 1	0	Serine	0
Art v 1	0	Cyn d 1	0	Mer a 1	0	Thioredoxin	0
Art v 3	0	Cup a 1	0	Mus m 1	0	Glutathione	0
Asp f 1	0	Der f 1	0	MUXF3	0	peroxiredoxin	0
Asp f 3	0	Der f 2	0	Ole e 1	0	Profilin	0
Asp f 6	0	Der p 1	0	Ole e 7	0	Dehydrin	0
Ber e 1	0	Der p 2	0	Ole e 9	0	purothionin	0
Bet v 1	0	V Der p 4	0	Par j 2	0	LTP	0
Bet v 2	0	V Der p 5	0	Pen m 1	0	V Ves v 1	0
Bet v 4	0	V Der p 7	0	Pen m 2	0	Ves v 5	0
Bla g 1	0	Der p 10	0	Pen m 4	0	V Ves v 5	0
Bla g 2	0	V Der p 11	0	Phl p 1	0	Blank	0

Donor no. NDP4							
Act d 1	0	Bla g 5	0	V Der p 14	0	Phl p 2	0
Act d 2	0	Bla g 7	0	V Der p 15	0	Phl p 4	0
Act d 5	0	Blo t 5	0	V Der p 18	0	Phl p 5	0
Act d 8	0	Bos d 4	0	V Der p 21	0	Phl p 6	0
Aln g 1	0	V Bos d 4	0	V Der p 23	0	Phl p 7	0
Alt a 1	0.79	Bos d 5	0	V clone 16	0	Phl p 11	0
Alt a 6	0	V Bos d 5	0	Equ c 1	0	Phl p 12	0
Amb a 1	0	Bos d Lactoferrin	0	Equ c 3	0	V Pis v 3	0
V Ana o 1	0	Bos d 8	0	Fag e 2	0	Pla a 1	0
Ana o 2	0	V Bos d 8	0	Fel d 1	2.27	Pla a 2	0
V Ana o 2	0	aS1-casein	0	Fel d 2	0	Pla a 3	0
V nAna o 2	0	aS2-casein	0	Fel d 4	0	Pla l 1	0
V Ana o 3	0	b-casein	0	Gad c 1	0	Pol d 5	0
Ani s 1	0	K-casein	0	Gal d 1	0	Pru p 1	0
Ani s 3	0	Transferrin	0	Gal d 2	0	Pru p 3	0
Api g 1	0	Bos d 6	0	Gal d 3	0	V Pru du 3	0
Api m 1	0	V BSA	0	Gal d 5	0	V Pru du 4	0
V Api m 1	0	Can f 1	0	Gly m 4	0	V Pru du 6	0
V Api m 2	0	Can f 2	0	Gly m 5	0	V Pru du 6.01	0
Api m 4	0	Can f 3	0	Gly m 6	0	V Pru du 6.02	0
V Api m 4	0	V Can f 4	0	Hev b 1	0	Sal k 1	0
Ara h 1	0	Can f 5	0	Hev b 3	0	Ses i 1	0
V Ara h 1	0	V Can f 5	0	Hev b 5	0	Tri a 14	0
Ara h 2	0	V Can f 6	0	Hev b 6.01	0	Tri a 19.0101	0
Ara h 3	0	Che a 1	0	Hev b 8	0	Tri a aA_TI	0
V Ara h 3	0	Cla h 8	0	Jug r 1	0	V Tri a 36 191	0
Ara h 6	0	Cor a 1.0401	0	Jug r 2	0	V Tri a 36	0
V Ara h 6	0	Cor a 8	0	Jug r 3	0	V m43	0
Ara h 8	0	Cor a 9	0	Lep d 2	0	V m82	0
Ara h 9	0	Cry j 1	0	Mal d 1	0	Serine	0
Art v 1	0	Cyn d 1	0	Mer a 1	0	Thioredoxin	0
Art v 3	0	Cup a 1	0	Mus m 1	0	Glutathione	0
Asp f 1	0	Der f 1	0	MUXF3	0	peroxiredoxin	0
Asp f 3	0	Der f 2	0	Ole e 1	0.25	Profilin	0
Asp f 6	0	Der p 1	0	Ole e 7	0	Dehydrin	0
Ber e 1	0	Der p 2	0	Ole e 9	0	purothionin	0
Bet v 1	0	V Der p 4	0	Par j 2	0	LTP	0
Bet v 2	0	V Der p 5	0	Pen m 1	0	V Ves v 1	0
Bet v 4	0	V Der p 7	0	Pen m 2	0	Ves v 5	0
Bla g 1	0	Der p 10	0	Pen m 4	0	V Ves v 5	0
Bla g 2	0	V Der p 11	0	Phl p 1	0	Blank	0

Donor no. NDP5							
Act d 1	0	Bla g 5	0	V Der p 14	0	Phl p 2	0
Act d 2	0	Bla g 7	0	V Der p 15	0	Phl p 4	0
Act d 5	0	Blo t 5	0	V Der p 18	0	Phl p 5	1.56
Act d 8	0	Bos d 4	0	V Der p 21	0	Phl p 6	0
Aln g 1	5.8	V Bos d 4	0	V Der p 23	0	Phl p 7	0
Alt a 1	0	Bos d 5	0	V clone 16	0	Phl p 11	0
Alt a 6	0	V Bos d 5	0	Equ c 1	0	Phl p 12	0
Amb a 1	1.67	Bos d Lactoferrin	0	Equ c 3	0	V Pis v 3	0.35
V Ana o 1	0	Bos d 8	0	Fag e 2	0	Pla a 1	0
Ana o 2	12.79	V Bos d 8	0	Fel d 1	0	Pla a 2	0
V Ana o 2	0	aS1-casein	0	Fel d 2	0	Pla a 3	0
V nAna o 2	0	aS2-casein	0	Fel d 4	0	Pla l 1	0
V Ana o 3	0	b-casein	0	Gad c 1	0	Pol d 5	0
Ani s 1	0	K-casein	0	Gal d 1	0	Pru p 1	0.16
Ani s 3	0	Transferrin	0	Gal d 2	0	Pru p 3	0
Api g 1	0.95	Bos d 6	0	Gal d 3	0	V Pru du 3	0
Api m 1	0	V BSA	0	Gal d 5	0	V Pru du 4	3.7
V Api m 1	0	Can f 1	0	Gly m 4	0.95	V Pru du 6	0
V Api m 2	0	Can f 2	0	Gly m 5	0	V Pru du 6.01	0
Api m 4	0	Can f 3	0	Gly m 6	0	V Pru du 6.02	0
V Api m 4	0	V Can f 4	0	Hev b 1	0	Sal k 1	0
Ara h 1	0	Can f 5	0	Hev b 3	0	Ses i 1	0
V Ara h 1	0	V Can f 5	0	Hev b 5	0	Tri a 14	0
Ara h 2	0	V Can f 6	0	Hev b 6.01	0	Tri a 19.0101	0
Ara h 3	0	Che a 1	0	Hev b 8	3.21	Tri a aA_TI	0
V Ara h 3	0	Cla h 8	0	Jug r 1	0	V Tri a 36 191	0
Ara h 6	0	Cor a 1.0401	3.27	Jug r 2	0	V Tri a 36	0
V Ara h 6	0	Cor a 8	0	Jug r 3	0	V m43	0
Ara h 8	0.34	Cor a 9	0	Lep d 2	0	V m82	0
Ara h 9	0	Cry j 1	0.14	Mal d 1	3.46	Serine	0
Art v 1	0	Cyn d 1	0.4	Mer a 1	0.42	Thioredoxin	0
Art v 3	0	Cup a 1	0	Mus m 1	0	Glutathione	0
Asp f 1	0	Der f 1	0	MUXF3	0	peroxiredoxin	0
Asp f 3	0	Der f 2	0	Ole e 1	0	Profilin	0.2
Asp f 6	0	Der p 1	0	Ole e 7	0	Dehydrin	0
Ber e 1	0	Der p 2	0	Ole e 9	0	purothionin	0
Bet v 1	22.63	V Der p 4	0	Par j 2	0	LTP	0
Bet v 2	0.41	V Der p 5	0	Pen m 1	0	V Ves v 1	0
Bet v 4	0	V Der p 7	0	Pen m 2	0	Ves v 5	0.45
Bla g 1	0	Der p 10	0	Pen m 4	0	V Ves v 5	0
Bla g 2	0	V Der p 11	0	Phl p 1	0	Blank	0

Donor no. NA1							
Act d 1	0	Bla g 5	0	V Der p 14	0	Phl p 2	0
Act d 2	0	Bla g 7	0	V Der p 15	0	Phl p 4	0
Act d 5	0	Blo t 5	0	V Der p 18	0	Phl p 5	0
Act d 8	0	Bos d 4	0	V Der p 21	0	Phl p 6	0
Aln g 1	0	V Bos d 4	0	V Der p 23	0	Phl p 7	0
Alt a 1	0	Bos d 5	0	V clone 16	0	Phl p 11	0
Alt a 6	0	V Bos d 5	0	Equ c 1	0	Phl p 12	0
Amb a 1	0	Bos d Lactoferrin	0	Equ c 3	0	V Pis v 3	0
V Ana o 1	0	Bos d 8	0	Fag e 2	0	Pla a 1	0.21
Ana o 2	0.25	V Bos d 8	0	Fel d 1	0	Pla a 2	0
V Ana o 2	0	aS1-casein	0	Fel d 2	0	Pla a 3	0
V nAna o 2	0	aS2-casein	0	Fel d 4	0.02	Pla l 1	0
V Ana o 3	0	b-casein	0	Gad c 1	0.24	Pol d 5	0
Ani s 1	0	K-casein	0	Gal d 1	0	Pru p 1	0
Ani s 3	0	Transferrin	0	Gal d 2	0	Pru p 3	0
Api g 1	0	Bos d 6	0	Gal d 3	0	V Pru du 3	0
Api m 1	0	V BSA	0	Gal d 5	0	V Pru du 4	0
V Api m 1	0	Can f 1	0	Gly m 4	0	V Pru du 6	0
V Api m 2	0	Can f 2	0	Gly m 5	0	V Pru du 6.01	0
Api m 4	0	Can f 3	0	Gly m 6	0	V Pru du 6.02	0
V Api m 4	0	V Can f 4	0	Hev b 1	0	Sal k 1	0
Ara h 1	0	Can f 5	0	Hev b 3	0	Ses i 1	0
V Ara h 1	0	V Can f 5	0	Hev b 5	0	Tri a 14	0
Ara h 2	0	V Can f 6	0	Hev b 6.01	0	Tri a 19.0101	0
Ara h 3	0	Che a 1	0	Hev b 8	0	Tri a aA_TI	0
V Ara h 3	0	Cla h 8	0	Jug r 1	0	V Tri a 36 191	0
Ara h 6	0	Cor a 1.0401	0	Jug r 2	0	V Tri a 36	0
V Ara h 6	0	Cor a 8	0	Jug r 3	0	V m43	0
Ara h 8	0	Cor a 9	0	Lep d 2	0	V m82	0
Ara h 9	0	Cry j 1	0	Mal d 1	0	Serine	0
Art v 1	0	Cyn d 1	0	Mer a 1	0	Thioredoxin	0
Art v 3	0	Cup a 1	0	Mus m 1	0	Glutathione	0
Asp f 1	0	Der f 1	0	MUXF3	0	peroxiredoxin	0
Asp f 3	0	Der f 2	0	Ole e 1	0	Profilin	0
Asp f 6	0	Der p 1	0	Ole e 7	0	Dehydrin	0
Ber e 1	0	Der p 2	0	Ole e 9	0	purothionin	0
Bet v 1	0	V Der p 4	0	Par j 2	0	LTP	0
Bet v 2	0	V Der p 5	0	Pen m 1	0	V Ves v 1	0
Bet v 4	0	V Der p 7	0	Pen m 2	0	Ves v 5	0
Bla g 1	0	Der p 10	0	Pen m 4	0	V Ves v 5	0
Bla g 2	0	V Der p 11	0	Phl p 1	0.16	Blank	0

Donor no. NA2							
Act d 1	0	Bla g 5	0	V Der p 14	0	Phl p 2	0
Act d 2	0	Bla g 7	0	V Der p 15	0	Phl p 4	0
Act d 5	0	Blo t 5	0	V Der p 18	0	Phl p 5	0
Act d 8	0	Bos d 4	0	V Der p 21	0	Phl p 6	0
Aln g 1	0	V Bos d 4	0	V Der p 23	0	Phl p 7	0
Alt a 1	0	Bos d 5	0	V clone 16	0	Phl p 11	0
Alt a 6	0	V Bos d 5	0	Equ c 1	0	Phl p 12	0
Amb a 1	0	Bos d Lactoferrin	0	Equ c 3	0	V Pis v 3	0
V Ana o 1	0	Bos d 8	0	Fag e 2	0	Pla a 1	0
Ana o 2	0	V Bos d 8	0	Fel d 1	0	Pla a 2	0
V Ana o 2	0	aS1-casein	0	Fel d 2	0	Pla a 3	0
V nAna o 2	0	aS2-casein	0	Fel d 4	0	Pla l 1	0
V Ana o 3	0	b-casein	0	Gad c 1	0	Pol d 5	0
Ani s 1	0	K-casein	0	Gal d 1	0	Pru p 1	0
Ani s 3	0	Transferrin	0	Gal d 2	0	Pru p 3	0
Api g 1	0	Bos d 6	0	Gal d 3	0	V Pru du 3	0
Api m 1	0.44	V BSA	0	Gal d 5	0	V Pru du 4	0
V Api m 1	0	Can f 1	0	Gly m 4	0	V Pru du 6	0
V Api m 2	0	Can f 2	0	Gly m 5	0.39	V Pru du 6.01	0
Api m 4	0	Can f 3	0	Gly m 6	0	V Pru du 6.02	0
V Api m 4	0	V Can f 4	0	Hev b 1	0.24	Sal k 1	0
Ara h 1	0	Can f 5	0	Hev b 3	0	Ses i 1	0
V Ara h 1	0	V Can f 5	0	Hev b 5	0	Tri a 14	0
Ara h 2	0	V Can f 6	0	Hev b 6.01	0	Tri a 19.0101	0.43
Ara h 3	0	Che a 1	0	Hev b 8	0	Tri a aA_TI	0
V Ara h 3	0	Cla h 8	0	Jug r 1	0	V Tri a 36 191	0
Ara h 6	0	Cor a 1.0401	0	Jug r 2	0	V Tri a 36	0
V Ara h 6	0	Cor a 8	0	Jug r 3	0	V m43	0
Ara h 8	0	Cor a 9	0	Lep d 2	0	V m82	0
Ara h 9	0	Cry j 1	0	Mal d 1	0	Serine	0
Art v 1	0	Cyn d 1	0	Mer a 1	0	Thioredoxin	0
Art v 3	0	Cup a 1	0	Mus m 1	0	Glutathione	0
Asp f 1	0	Der f 1	0	MUXF3	0	peroxiredoxin	0
Asp f 3	0	Der f 2	0	Ole e 1	0	Profilin	0.27
Asp f 6	0	Der p 1	0	Ole e 7	0	Dehydrin	0
Ber e 1	0	Der p 2	0	Ole e 9	0	purothionin	0
Bet v 1	0	V Der p 4	0	Par j 2	0	LTP	0
Bet v 2	0	V Der p 5	0	Pen m 1	0	V Ves v 1	0
Bet v 4	0	V Der p 7	0	Pen m 2	0	Ves v 5	0
Bla g 1	0	Der p 10	0	Pen m 4	0	V Ves v 5	0
Bla g 2	0	V Der p 11	0	Phl p 1	0.25	Blank	0

Donor no. NA3							
Act d 1	0	Bla g 5	0	V Der p 14	0	Phl p 2	0
Act d 2	0	Bla g 7	0	V Der p 15	0	Phl p 4	0
Act d 5	0	Blo t 5	0	V Der p 18	0	Phl p 5	0
Act d 8	0	Bos d 4	0	V Der p 21	0	Phl p 6	0
Aln g 1	0	V Bos d 4	0	V Der p 23	0	Phl p 7	0
Alt a 1	0	Bos d 5	0	V clone 16	0	Phl p 11	0
Alt a 6	0	V Bos d 5	0	Equ c 1	0	Phl p 12	0
Amb a 1	0	Bos d Lactoferrin	0	Equ c 3	0	V Pis v 3	0
V Ana o 1	0	Bos d 8	0	Fag e 2	0	Pla a 1	0
Ana o 2	0	V Bos d 8	0	Fel d 1	0	Pla a 2	0
V Ana o 2	0	aS1-casein	0	Fel d 2	0	Pla a 3	0
V nAna o 2	0	aS2-casein	0	Fel d 4	0	Pla l 1	0
V Ana o 3	0	b-casein	0	Gad c 1	0	Pol d 5	0
Ani s 1	0	K-casein	0	Gal d 1	0	Pru p 1	0
Ani s 3	0	Transferrin	0	Gal d 2	0	Pru p 3	0
Api g 1	0	Bos d 6	0	Gal d 3	0	V Pru du 3	0
Api m 1	0	V BSA	0	Gal d 5	0	V Pru du 4	0
V Api m 1	0	Can f 1	0	Gly m 4	0	V Pru du 6	0
V Api m 2	0	Can f 2	0	Gly m 5	0	V Pru du 6.01	0
Api m 4	0	Can f 3	0	Gly m 6	0	V Pru du 6.02	0
V Api m 4	0	V Can f 4	0	Hev b 1	0	Sal k 1	0
Ara h 1	0	Can f 5	0	Hev b 3	0	Ses i 1	0
V Ara h 1	0	V Can f 5	0	Hev b 5	0	Tri a 14	0
Ara h 2	0	V Can f 6	0	Hev b 6.01	0	Tri a 19.0101	0
Ara h 3	0	Che a 1	0	Hev b 8	0	Tri a aA_TI	0
V Ara h 3	0	Cla h 8	0	Jug r 1	0	V Tri a 36 191	0
Ara h 6	0	Cor a 1.0401	0	Jug r 2	0	V Tri a 36	0
V Ara h 6	0	Cor a 8	0	Jug r 3	0	V m43	0
Ara h 8	0	Cor a 9	0	Lep d 2	0	V m82	0
Ara h 9	0	Cry j 1	0	Mal d 1	0	Serine	0
Art v 1	0	Cyn d 1	0	Mer a 1	0	Thioredoxin	0
Art v 3	0	Cup a 1	0	Mus m 1	0	Glutathione	0
Asp f 1	0	Der f 1	0	MUXF3	0	peroxiredoxin	0
Asp f 3	0	Der f 2	0	Ole e 1	0	Profilin	0
Asp f 6	0	Der p 1	0	Ole e 7	0	Dehydrin	0
Ber e 1	0	Der p 2	0	Ole e 9	0	purothionin	0
Bet v 1	0	V Der p 4	0	Par j 2	0	LTP	0
Bet v 2	0	V Der p 5	0	Pen m 1	0	V Ves v 1	0
Bet v 4	0	V Der p 7	0	Pen m 2	0	Ves v 5	0
Bla g 1	0	Der p 10	0	Pen m 4	0	V Ves v 5	0
Bla g 2	0	V Der p 11	0	Phl p 1	0	Blank	0

Donor no. NA4							
Act d 1	0	Bla g 5	0	V Der p 14	0	Phl p 2	0
Act d 2	0	Bla g 7	0	V Der p 15	0	Phl p 4	0
Act d 5	0	Blo t 5	0	V Der p 18	0	Phl p 5	0
Act d 8	0	Bos d 4	0	V Der p 21	0	Phl p 6	0
Aln g 1	0	V Bos d 4	0	V Der p 23	0	Phl p 7	0
Alt a 1	0	Bos d 5	0	V clone 16	0	Phl p 11	0
Alt a 6	0	V Bos d 5	0	Equ c 1	0	Phl p 12	0
Amb a 1	0	Bos d Lactoferrin	0	Equ c 3	0	V Pis v 3	0
V Ana o 1	0	Bos d 8	0	Fag e 2	0	Pla a 1	0
Ana o 2	0	V Bos d 8	0	Fel d 1	0	Pla a 2	0
V Ana o 2	0	aS1-casein	0	Fel d 2	0	Pla a 3	0
V nAna o 2	0	aS2-casein	0	Fel d 4	0	Pla l 1	0
V Ana o 3	0	b-casein	0	Gad c 1	0	Pol d 5	0.34
Ani s 1	0	K-casein	0	Gal d 1	0	Pru p 1	0
Ani s 3	0	Transferrin	0	Gal d 2	0	Pru p 3	0
Api g 1	0	Bos d 6	0	Gal d 3	0	V Pru du 3	0
Api m 1	0	V BSA	0	Gal d 5	0	V Pru du 4	0
V Api m 1	0	Can f 1	0	Gly m 4	0	V Pru du 6	0
V Api m 2	0	Can f 2	0	Gly m 5	0	V Pru du 6.01	0
Api m 4	0	Can f 3	0	Gly m 6	0	V Pru du 6.02	0
V Api m 4	0	V Can f 4	0	Hev b 1	0	Sal k 1	0
Ara h 1	0	Can f 5	0	Hev b 3	0	Ses i 1	0
V Ara h 1	0	V Can f 5	0	Hev b 5	0	Tri a 14	0
Ara h 2	0	V Can f 6	0	Hev b 6.01	0	Tri a 19.0101	0
Ara h 3	0	Che a 1	0	Hev b 8	0	Tri a aA_TI	0
V Ara h 3	0	Cla h 8	0	Jug r 1	0	V Tri a 36 191	0
Ara h 6	0	Cor a 1.0401	0	Jug r 2	0	V Tri a 36	0
V Ara h 6	0	Cor a 8	0	Jug r 3	0	V m43	0
Ara h 8	0	Cor a 9	0	Lep d 2	0	V m82	0
Ara h 9	0	Cry j 1	0	Mal d 1	0	Serine	0
Art v 1	0	Cyn d 1	0	Mer a 1	0	Thioredoxin	0
Art v 3	0	Cup a 1	0	Mus m 1	0	Glutathione	0
Asp f 1	0	Der f 1	0	MUXF3	0	peroxiredoxin	0
Asp f 3	0	Der f 2	0	Ole e 1	0	Profilin	0
Asp f 6	0	Der p 1	0	Ole e 7	0	Dehydrin	0
Ber e 1	0	Der p 2	0	Ole e 9	0	purothionin	0
Bet v 1	0	V Der p 4	0	Par j 2	0	LTP	0
Bet v 2	0	V Der p 5	0	Pen m 1	0	V Ves v 1	0
Bet v 4	0	V Der p 7	0	Pen m 2	0	Ves v 5	0.8
Bla g 1	0	Der p 10	0	Pen m 4	0	V Ves v 5	0
Bla g 2	0	V Der p 11	0	Phl p 1	0	Blank	0

Donor no. NA5							
Act d 1	0	Bla g 5	0	V Der p 14	0	Phl p 2	0
Act d 2	0	Bla g 7	0	V Der p 15	0	Phl p 4	0.27
Act d 5	0	Blo t 5	0	V Der p 18	0	Phl p 5	0
Act d 8	0	Bos d 4	0	V Der p 21	0	Phl p 6	0
Aln g 1	0	V Bos d 4	0	V Der p 23	0	Phl p 7	0
Alt a 1	0	Bos d 5	0	V clone 16	0	Phl p 11	0
Alt a 6	0	V Bos d 5	0	Equ c 1	0	Phl p 12	0
Amb a 1	0	Bos d Lactoferrin	0	Equ c 3	0	V Pis v 3	0
V Ana o 1	0	Bos d 8	0	Fag e 2	0	Pla a 1	0
Ana o 2	0	V Bos d 8	0	Fel d 1	0	Pla a 2	0
V Ana o 2	0	aS1-casein	0	Fel d 2	0	Pla a 3	0
V nAna o 2	0	aS2-casein	0	Fel d 4	0	Pla l 1	0
V Ana o 3	0	b-casein	0	Gad c 1	0	Pol d 5	0
Ani s 1	0	K-casein	0	Gal d 1	0	Pru p 1	0
Ani s 3	0	Transferrin	0	Gal d 2	0	Pru p 3	0
Api g 1	0	Bos d 6	0	Gal d 3	0	V Pru du 3	0
Api m 1	0	V BSA	0	Gal d 5	0	V Pru du 4	0
V Api m 1	0	Can f 1	0	Gly m 4	0	V Pru du 6	0
V Api m 2	0	Can f 2	0	Gly m 5	0	V Pru du 6.01	0
Api m 4	0	Can f 3	0	Gly m 6	0	V Pru du 6.02	0
V Api m 4	0	V Can f 4	0	Hev b 1	0	Sal k 1	0
Ara h 1	0	Can f 5	0	Hev b 3	0	Ses i 1	0
V Ara h 1	0	V Can f 5	0	Hev b 5	0	Tri a 14	0
Ara h 2	0	V Can f 6	0	Hev b 6.01	0	Tri a 19.0101	0
Ara h 3	0	Che a 1	0	Hev b 8	0	Tri a aA_TI	0
V Ara h 3	0	Cla h 8	0	Jug r 1	0	V Tri a 36 191	0
Ara h 6	0	Cor a 1.0401	0	Jug r 2	0	V Tri a 36	0
V Ara h 6	0	Cor a 8	0	Jug r 3	0	V m43	0
Ara h 8	0	Cor a 9	0	Lep d 2	0	V m82	0
Ara h 9	0	Cry j 1	0	Mal d 1	0	Serine	0
Art v 1	0	Cyn d 1	0	Mer a 1	0	Thioredoxin	0
Art v 3	0	Cup a 1	0	Mus m 1	0	Glutathione	0
Asp f 1	0	Der f 1	0	MUXF3	0	peroxiredoxin	0
Asp f 3	0	Der f 2	0	Ole e 1	0	Profilin	0
Asp f 6	0	Der p 1	0	Ole e 7	0	Dehydrin	0
Ber e 1	0	Der p 2	0	Ole e 9	0	purothionin	0
Bet v 1	0	V Der p 4	0	Par j 2	0	LTP	0
Bet v 2	0	V Der p 5	0	Pen m 1	0	V Ves v 1	0
Bet v 4	0	V Der p 7	0	Pen m 2	0	Ves v 5	0
Bla g 1	0	Der p 10	0	Pen m 4	0	V Ves v 5	0
Bla g 2	0	V Der p 11	0	Phl p 1	0	Blank	0
