# Supplementary material for: A hypoallergenic peptide mix containing T cell epitopes of the clinically relevant house dust mite allergens
Source: Allergy. 2019 Oct 3;74(12):2461–78. doi: 10.1111/all.13956 (PMC7078969; doi:10.1111/all.13956)
Supplement: Supplementary file 6 [file ALL-74-2461-s006.pdf]

Table S4. Concentrations of peptides equimolar to 100 ng/ml of each allergen in basophil activation tests.

	No.	No. of aa	Molecular weight (Da)	Ratio ($Mw_{peptide}/Mw_{protein}$)	ng/ml (when 100 ng/ml allergen)
Der p 1	1	41	4284.8	0.17	17.14
	2	43	4767.1	0.19	19.07
	3	31	3686.0	0.15	14.74
	4	37	4359.9	0.17	17.44
	5	31	3690.0	0.15	14.76
	6	33	3783.1	0.15	15.13
	7	34	3874.1	0.15	15.50
	8	35	4130.6	0.17	16.52
Der p 2	1	33	3626.1	0.26	25.90
	2	31	3440.9	0.25	24.58
	3	32	3354.7	0.24	23.96
	4	42	4789.5	0.34	34.21
	5	32	3307.8	0.24	23.63
Der p 5	1	30	3735.2	0.27	26.68
	2	37	4399.1	0.31	31.42
	3	32	3729.3	0.27	26.64
Der p 7	1	31	3494.8	0.16	15.89
	2	32	3642.1	0.17	16.56
	3	32	3589.1	0.16	16.31
	4	37	4230.7	0.19	19.23
	5	27	3032.3	0.14	13.78
	5-6	29	3072.5	0.14	13.97
	6	30	3452.9	0.16	15.70
Der p 21	1	35	4233.9	0.30	30.24
	2	38	4479.2	0.32	31.99
	3	30	3697.3	0.26	26.41
	4	34	4052.7	0.29	28.95
Der p 23	1	32	3582.7	0.45	44.78
	2	34	3982.3	0.50	49.78
	4	29	3340.6	0.42	41.76
	5	29	3476.7	0.43	43.46
