# Supplementary material for: A hypoallergenic peptide mix containing T cell epitopes of the clinically relevant house dust mite allergens
Source: Allergy. 2019 Oct 3;74(12):2461–78. doi: 10.1111/all.13956 (PMC7078969; doi:10.1111/all.13956)
Supplement: Supplementary file 7 [file ALL-74-2461-s007.pdf]

Table S5. Percentages of HDM-sensitized and non-sensitized subjects showing positive IgG binding to allergens or peptides

	Sensitized	Non-HDM sensitized
**Der p 1**	100.0	90.0
P1	0.0	0.0
P2	30.4	20.0
P3	4.3	10.0
P4	4.3	20.0
P5	17.4	10.0
P6	0.0	0.0
P7	0.0	0.0
P8	21.7	0.0
**Der p 2**	61.9	10.0
P1	14.3	20.0
P2	9.5	0.0
P3	9.5	0.0
P4	0.0	0.0
P5	4.8	0.0
**Der p 5**	92.9	40.0
P1	42.9	20.0
P2	0.0	0.0
P3	0.0	0.0
P4	28.6	10.0
**Der p 7**	100.0	60.0
P1	42.9	20.0
P2	57.1	20.0
P3	57.1	50.0
P4	28.6	20.0
P5	0.0	0.0
P5-6	0.0	0.0
P6	0.0	10.0
**Der p 21**	100.0	60.0
P1	15.4	20.0
P2	15.4	30.0
P3	7.7	10.0
P4	15.4	10.0
**Der p 23**	100.0	70.0
P1	11.1	30.0
P2	27.8	20.0
P3	66.7	30.0
P4	22.2	10.0
P5	22.2	20.0
