# Supplementary material for: A hypoallergenic peptide mix containing T cell epitopes of the clinically relevant house dust mite allergens
Source: Allergy. 2019 Oct 3;74(12):2461–78. doi: 10.1111/all.13956 (PMC7078969; doi:10.1111/all.13956)
Supplement: Supplementary file 8 [file ALL-74-2461-s008.docx]

**Online Repository:**

**A hypoallergenic peptide mix containing T cell epitopes of the clinically relevant house dust mite allergens**

**Running title: T cell epitopes of the most relevant house dust mite allergens**

Huey-Jy Huang, MSc,^a^ Mirela Curin, PhD,^a^ Srinita Banerjee, PhD,^a^ Kuan-Wei Chen, PhD,^a^ Tetiana Garmatiuk, MD,^a^ Yvonne Resch-Marat, PhD,^a^ Claudia Carvalho-Queiroz, PhD,^b^ Katharina Blatt, PhD,^c^ Guro Gafvelin, PhD,^b^ Hans Grönlund, PhD,^b^ Peter Valent, MD,^c^ Raffaela Campana, PhD,^a^ Margarete Focke-Tejkl, PhD,^a^ Rudolf Valenta, MD,^a,d,e^ Susanne Vrtala, PhD,^a^

^a^Division of Immunopathology, Department of Pathophysiology and Allergy Research, Center for Pathophysiology, Infectiology and Immunology, Medical University of Vienna, Vienna, Austria

^b^Therapeutic Immune Design Unit, Department of Clinical Neuroscience, Karolinska Institutet, Stockholm, Sweden

^c^Department of Internal Medicine I, Division of Hematology&Hemostaseology, Medical University of Vienna, Austria

^d^NRC Institute of Immunology FMBA of Russia, Moscow, Russia

^e^Laboratory for Immunopathology, Department of Clinical Immunology and Allergy, Sechenov First Moscow State Medical University, Moscow, Russia

Corresponding Author: Dr. Susanne Vrtala

Division of Immunopathology, Department of Pathophysiology and Allergy Research, Center for Pathophysiology, Infectiology and Immunology, Medical University of Vienna

Waehringer Guertel 18-20, 1090 Vienna, Austria

Phone: +43 (0)1 40400 - 51320
FAX: +43 (0)1 40400 - 51300

E-mail: susanne.vrtala@meduniwien.ac.at

**SUPPLEMENTAL FIGURES AND TABLES**

**Figure S1.** T cell reactivity of Der p allergens and allergen-derived peptides. PBMCs from individuals sensitized to the respective allergen (red) and individuals without HDM sensitization (black) were incubated with allergens (Der p 1, Der p 2, Der p 5, Der p 7, Der p 21, Der p 23), and the allergen-derived peptides (x-axes). Shown are percentages of proliferated CD4+ T cells in box plots representing the first and third quartile. Horizontal bars denote medians and outliers are presented as circles. The number of allergic patients tested was: Der p 1: 23, Der p 2: 21, Der p 5: 14, Der p 7: 7, Der p 21: 13, Der p 23: 18, non-HDM allergic individuals: 10. Statistically significant differences (*P < 0.05) are indicated. ~~.~~

**Figure S2.** Cytokine and chemokine responses to Der p allergens and allergen-derived peptides. Cultured PBMCs from individuals sensitized to the respective allergen (red) and individuals without HDM sensitization (black: n=10) were incubated with allergens (Der p 1, Der p 2, Der p 5, Der p 7, Der p 21, Der p 23) and the corresponding allergen-derived peptides (x-axes) and cytokine and chemokine levels were measured in the supernatants (y-axis: pg/ml). ***A,*** IL-1 beta, ***B,*** IL-6, ***C,*** TNF-alpha, ***D,*** GM-CSF, ***E,*** MCP-1, ***F,*** MIP-1beta, and ***G,*** G-CSF. Shown are box plots representing the first and third quartile. Horizontal bars denote medians and outliers are presented as circles. Statistically significant differences (**P < 0.01; *P < 0.05) are indicated.

**Table S1.** Characteristics of allergen-derived peptides.

**Table S2.** Demographic and clinical characteristics of 27 HDM-allergic patients, 5 non-HDM-sensitized allergic patients and 5 non-allergic individuals.

**Table S3.** IgE reactivity of HDM-allergic patients (PA), non-HDM-sensitized allergic patients (NDP) and non-allergic individuals (NA) to micro-arrayed allergens determined by ImmunoCAP ISAC technology.

**Table S4.** Concentrations of peptides equimolar to 100 ng/ml of each allergen in basophil activation tests.

**Table S5.** Percentages of HDM-sensitized and non-sensitized subjects showing positive IgG binding over cut-off to allergens or peptides.
